# Products of Selenite/Thiols Interaction Have Reducing Properties, Cleave Plasmid DNA and Decrease Rat Blood Pressure and Tension of Rat Mesenteric Artery

**DOI:** 10.1007/s12011-024-04196-3

**Published:** 2024-04-27

**Authors:** Marian Grman, Peter Balis, Andrea Berenyiova, Helena Svajdlenkova, Lenka Tomasova, Sona Cacanyiova, Zuzana Rostakova, Iveta Waczulikova, Miroslav Chovanec, Enrique Domínguez-Álvarez, Karol Ondrias, Anton Misak

**Affiliations:** 1 Institute of Clinical and Translational Research, Biomedical Research Center, Slovak Academy of Sciences, Dubravska Cesta 9, 845 05 Bratislava, Slovak Republic; 2 Institute of Normal and Pathological Physiology, Centre of Experimental Medicine, Slovak Academy of Sciences, Sienkiewiczova 1, 813 71 Bratislava, Slovak Republic; 3 Polymer Institute, Slovak Academy of Sciences, Dubravska Cesta 9, 845 41 Bratislava, Slovak Republic; 4 Institute of Measurement Science, Slovak Academy of Sciences, Dubravska Cesta 9, 841 04 Bratislava, Slovak Republic; 5 Department of Nuclear Physics and Biophysics, Faculty of Mathematics, Physics and Informatics, Comenius University, Mlynska Dolina F1, 842 48 Bratislava, Slovak Republic; 6 Cancer Research Institute, Slovak Academy of Sciences, Dubravska Cesta 9, 845 05 Bratislava, Slovak Republic; 7 Instituto de Química Orgánica General (IQOG), CSIC, Juan de La Cierva 3, 28006 Madrid, Spain; 8 Department of Nuclear Chemistry, Faculty of Natural Sciences, Comenius University, Ilkovicova 6, 842 15 Bratislava, Slovak Republic

**Keywords:** Thiols, Selenite, Rat blood pressure, Vascular reactivity, •cPTIO reduction, Plasmid DNA cleavage

## Abstract

Selenium compounds exert their antioxidant activity mostly when the selenium atom is incorporated into selenoproteins. In our work, we tested the possibility that selenite itself interacts with thiols to form active species that have reducing properties. Therefore, we studied the reduction of 2-(4-carboxyphenyl)-4,5-dihydro-4,4,5,5-tetramethyl-1*H*-imidazol-1-yloxy-3-oxide radical (^•^cPTIO), damage of plasmid DNA (pDNA), modulation of rat hemodynamic parameters and tension of isolated arteries induced by products of interaction of selenite with thiols. We found that the products of selenite interaction with thiols had significant reducing properties that could be attributed mainly to the selenide and that selenite had catalytic properties in the access of thiols. The potency of thiols to reduce ^•^cPTIO in the interaction with selenite was cysteine > homocysteine > glutathione reduced > N-acetylcysteine. Thiol/selenite products cleaved pDNA, with superoxide dismutase enhancing these effects suggesting a positive involvement of superoxide anion in the process. The observed ^•^cPTIO reduction and pDNA cleavage were significantly lower when selenomethionine was used instead of selenite. The products of glutathione/selenite interaction affected several hemodynamic parameters including rat blood pressure decrease. Notably, the products relaxed isolated mesenteric artery, which may explain the observed decrease in rat blood pressure. In conclusion, we found that the thiol/selenite interaction products exhibited significant reducing properties which can be used in further studies of the treatment of pathological conditions caused by oxidative stress. The results of decreased rat blood pressure and the tension of mesenteric artery may be perspective in studies focused on cardiovascular disease and their prevention.

**Supplementary Information:**

The online version contains supplementary material available at 10.1007/s12011-024-04196-3.

## Introduction

Selenium (Se) plays a crucial role in human health. As an essential trace element, the levels of Se in the organism depend on the diet. When there is a lack of Se in diet, inorganic selenite (SeO_3_^2−^) and organic selenomethionine (SeMet) are mostly used as food supplements. Both beneficial and detrimental effects of Se consumption are well known. Accumulating evidence supports the protective role of Se and selenoproteins in several diseases, including cancer, diabetes, neurodegenerative and cardiovascular disorders, inflammation or infections [1–6]. However, chronic overconsumption of Se may lead to intoxication with compromised functions of the liver, kidney, immune and reproductive system. In this context, inadequate Se levels were associated with the development of cancer, cardiovascular and liver diseases [6–9]. Notably, inorganic forms of Se as SeO_3_^2−^ can exert toxic effects, that could be enhanced in Se overconsumption; whereas the organic forms (SeMet, methylselenocysteine) are much less toxic than inorganic forms [10].

On the cellular level, Se acts as an antioxidant that eliminates the harmful effects of the free radicals by regulating the synthesis of glutathione reductase and other selenoproteins and by increasing the total antioxidant capacity levels [6, 11, 12]. However, SeO_3_^2−^ acting as oxidant, oxidizes most thiols in vitro*,* thereby generating superoxide and other reactive oxygen species (ROS) in cells and cell membranes, which might be associated with the observed SeO_3_^2−^-mediated toxicity and free radical stress in vivo [5, 13–16].

Chemical reactions of SeO_3_^2−^ with thiols have already been studied and discussed. Upon oxidation of GSH by SeO_3_^2−^, selenodiglutathione (GSSeSG), selenopersulfide (RSSeH), selenopersulfide anion (RSSe^−^), selenide (Se^2–^) and a wide range of intermediate reactive sulfur-selenium species are formed including superoxide (^•^O_2_^–^) [2, 17–21].

In ex vivo or in vivo studies, depending on the doses used, mostly beneficial effects of SeO_3_^2−^ have been observed, e.g. it decreased the ROS levels, lipid peroxidation, oxidative damage and inflammation in atherosclerotic and ischemic stroke patients and in models of ischemia–reperfusion injury [22–27]. Furthermore, SeO_3_^2–^ inhibited endoplasmic reticulum stress, ferroptosis, neurotoxicity and cardiotoxicity in cells and in experimental animals [28–31], but activated endoplasmic reticulum stress by increasing the generation of ROS leading to cancer cells death [32–35].

It has been reported that Se compounds are not antioxidants by themselves: they exert their antioxidant activity mostly when its Se atom is incorporated into selenoproteins with oxidoreductase functions and when they are supplied at dietary dose levels corresponding to physiological optimal concentrations [5, 6, 15]. When the levels of Se compounds surpass the capacity of selenoproteins biosynthesis, any additional supply of Se leads to the formation of Se metabolites, which are capable to generate ROS upon reaction and/or oxidation of cellular thiols [2, 5, 13].

ROS are responsible for oxidative stress, which contributes negatively to many diseases and substances that have antioxidant properties may be suitable for reducing oxidative stress [3, 11, 12, 35–37]. Therefore in our work, we tested the hypotheses that SeO_3_^2−^ itself interacts with glutathione (GSH), cysteine (Cys) or homocysteine (HCys) to form active species that have reducing/antioxidant properties, in addition to the antioxidant effects of Se when it is incorporated into the selenoproteins. In our previous study, SeO_3_^2−^ alone neither reduced the ^•^cPTIO stable radical nor scavenged the ^•^BMPO-OOH/OH radical, but in the presence of H_2_S/HS^−^, it had significant antioxidant properties [38, 39]. Based on this, the first aim of the present work was to ascertain whether SeO_3_^2−^, when interacting with a panel of thiols, forms thiol/SeO_3_^2−^ active species, which would have reducing properties, as found for (H_2_S/HS^−^)/SeO_3_^2−^ species [38].

Se supplementation can inhibit the growth of various types of cancer cells, and therefore Se compounds are studied as novel potential anticancer agents [3, 4, 33, 34, 40–43]. A possible mechanism has been proposed that Se compounds can increase the level of ROS, which act as cell cycle inhibitors in cancer cells [33, 34, 42, 44]. SeO_3_^2−^ induced DNA single- and/or double-strands breaks in hepatocytes or L1210 cells [45–47]. However, SeO_3_^2−^ alone did not cleave plasmid DNA (pDNA) in vitro, but caused damage to pDNA in the presence of H_2_S/HS^−^ [38]. Based on this, the second aim of our work was to examine whether SeO_3_^2−^, when interacting with a panel of thiols, forms active species, which would cleave pDNA, as found for (H_2_S/HS^−^)/SeO_3_^2−^ species. We also investigated if ^•^O_2_^−^ is involved in the effects of thiol/SeO_3_^2−^ on pDNA.

ROS and antioxidants play an important role in the cardiovascular system [48–50]. Since thiol/SeO_3_^2−^ might have antioxidant properties, the third aim was to study the effects of thiol/SeO_3_^2−^ firstly on hemodynamic parameters of anesthetized rat in vivo and secondly on vascular reactivity of isolated arteries ex vivo.

The exact mechanisms of the beneficial and toxic effects of Se are not yet fully understood, giving rise to further uncertainty about its potential use in nutrition supplements and/or clinical treatment. Our work was aimed to understand numerous biological effects of Se and its derivatives by studying the effects of thiols/SeO_3_^2−^ products to reduce the ^•^cPTIO stable radical, damage plasmid DNA, affecting rat cardiovascular system in vivo and vascular reactivity of isolated arteries.

## Materials and Methods

### Chemicals and Solutions

The following chemicals were bought from Sigma-Aldrich (Schnelldorf, Germany): 2-(4-carboxyphenyl)-4,5-dihydro-4,4,5,5-tetramethyl-1*H*-imidazol-1-yloxy-3-oxide potassium salt (^•^cPTIO; C221), 2,2,6,6-tetramethylpiperidine 1-oxyl, 2,2,6,6-tetramethyl-1-piperidinyloxy (TEMPO, 214,000), sodium selenite (Na_2_SeO_3_, SeO_3_^2−^; 214,485), sodium selenate (Na_2_SeO_4_, SeO_4_^2−^; S0882), sodium selenide (Na_2_Se, Se^2−^; 796,948), selenium tetrachloride (SeCl_4_; 323,527), L-cysteine hydrochloride (Cys; C1276), DL-homocysteine (HCys; H4628), D-cysteine (DCys; 30,095), L-cystine dihydrochloride (cystine; C6727), L-glutathione reduced (GSH; G4251), glutathione oxidized (GSSG; G6654), N-acetyl-L-cysteine (NAC; A7250), L-methionine (MET; M9625), ( ±)-6-hydroxy-2,5,7,8-tetramethylchromane-2-carboxylic acid (Trolox; 238,813), diethylenetriaminepentaacetic acid (DTPA, D6518), silver nitrate (AgNO_3_, 85,228), potassium ferricyanide (K_3_Fe(CN)_6_, 702,587), hydrogen peroxide (H_2_O_2_, 85,321), superoxide dismutase (SOD, S7446), catalase (CAT, C9322), serotonin creatinine sulfate monohydrate (Ser, H7752), sodium phosphate monobasic (NaH_2_PO_4_, S5011) and sodium phosphate dibasic (Na_2_HPO_4_, S7907). Seleno-L-methionine (SeMet; 1,611,955) was acquired from United States Pharmacopeia. Noradrenalin Léčiva (NA, 2,010,223) was purchased from Zentiva (Prague, Czech Republic). Isoflurin (Isoflurane, 1000 mg g^−1^) was purchased from Vetpharma (Barcelona, Spain). Spin trap 5-*tert-*Butoxycarbonyl-5-methyl-1-pyrroline-*N*-oxide (BMPO, B568) was acquired from DoJindo Molecular Technologies (Kumamoto, Japan) and its 100 mM stock solution prepared in deionized water was stored at − 80 °C. Stock solutions (10 or 100 mmol L^−1^) of the studied compounds and SeO_3_^2−^ were prepared fresh every day in ultrapure deionized H_2_O, stored at 23°C and used within 8 h, unless it is stated otherwise. Cystine (10 mmol L^−1^) was prepared 5 s before application. ^•^cPTIO (10 mmol L^−1^) was stored at − 20 °C for several days. Trolox (10 mmol L^−1^) and H_2_O_2_ (2 mmol L^−1^) was prepared fresh every day in sodium phosphate buffer (100 mmol L^−1^ sodium phosphate, 100 µmol L^−1^ DTPA buffer, pH 7.4 at 37 °C). SOD and CAT were solubilised in 100 and 50 mmol L^−1^ sodium phosphate (pH 7.4), respectively, and stored at − 80 °C until use. TEMPO (100 mmol L^−1^) was prepared in 100 mmol L^−1^ phosphate buffer, pH 7.4. Na_2_Se was dissolved in deionized water to get 10 mmol L^−1^ solution or phosphate buffer (100 mmol L^−1^, 7.4 pH) containing 0.85 mmol L^−1 •^cPTIO was added directly to Na_2_Se powder (≤ 0.1 − 0.2 mg). Serotonin was prepared in a given aliquot in deionized water and kept at − 20 °C. Noradrenalin was prepared fresh daily in deionized water.

### UV–Vis of the ^•^cPTIO Radical

Reduction of ^•^cPTIO stable radical by thiol/SeO_3_^2−^ mixture was performed like in our previous study [38]. The stock solution (10–50 µL) of the studied compounds was added to 950–990 µL of sodium phosphate buffer (100 mmol L^−1^ sodium phosphate, 100 µmol L^−1^ DTPA, pH 7.4, 37°C) (unless otherwise stated) containing 100 µmol L^−1 •^cPTIO and UV–Vis absorption spectra (900–220 nm, optical pathway 10 mm) were recorded every 30 s (or 1 min) for 30 min (or 120 min) with a Shimadzu 1800 (Kyoto, Japan) spectrometer at 37°C. Reduction of the ^•^cPTIO radical was determined as the decrease of the absorbance (ABS) at 560 nm [51]. To compare the reduction rates, the time-dependent data of the time decrease of ABS at 560 nm were well fitted by the equation of exponential decay: $f={ABS}_{0}+a\times {e}^{-b\times t}$, where ABS_0_ represents ABS at initial time, *t* is the time in minutes. The parameters ‘*a*’ (arbitrary units) and ‘*b*’ (min^–1^) were evaluated from the fitted data, a higher ‘*b*’ means a higher rate of ^•^cPTIO reduction.

### EPR of the ^•^BMPO-adducts

To study an involvement of radicals in GSH/SeO_3_^2−^ interaction, EPR study of spin trap BMPO was used and conducted in accordance with previously reported protocols [52]. To the phosphate buffer (50 mmol L^−1^, pH 7.4, 37°C) with DTPA (50 μmol L^−1^) containing BMPO (final concentration 30 mmol L^−1^), aliquots of the compounds were added. After short mixing (5 s), the sample was incubated for 10 s at 24°C or 60–600 s at 37°C and transferred to a standard cavity aqueous EPR flat cell. EPR spectra of the ^•^BMPO spin-adducts were measured on a Varian E4 spectrometer, X-band ~ 9.52 GHz, 341 mT central field, 10 mT scan range, 1–10 mW microwave power, 0.2 mT modulation amplitude at 24°C.

### Plasmid DNA Cleavage

A pDNA cleavage assay with the use of pBR322 plasmid (New England BioLabs, Inc., N3033 L, Ipswich, MA, USA) was performed as previously published [39]. All the samples used in this assay contained 0.2 μg pDNA in the final volume of 20 µL of sodium phosphate buffer (25 mmol L^−1^ sodium phosphate, 50 μmol L^−1^ DTPA, pH 7.4 at 37°C). To prepare 20 µL reaction sample and to start the reaction at the same time, 5 µL of the SeO_3_^2−^ (0.04 mmol L^−1^) or SeMet (2.4 mmol L^−1^) aqueous stock solution were added to 15 µL of pDNA solution containing thiols GSH, Cys, NAC, HCys, MET, GSSG or cystine. The final concentration of thiols in the case of SeO_3_^2−^ (0.01 mmol L^−1^ final) was: 0.08, 0.16, 0.32, 0.64, 1.28, 2.56 and 5.12 mmol L^−1^. In the case of SeMet (0.6 mmol L^−1^ final), it was 0.12, 0.20, 0.30, 0.60, 1.20, 1.80 and 3.00 mmol L^−1^. To study the participation of ROS in pDNA cleavage that was induced by GSH (0.17, 0.72 and 3.00 in mmol L^−1^) and SeO_3_^2−^ (0.01 mmol L^−1^) mixture, the SOD (200 U mL^−1^), CAT (200 U mL^−1^) and H_2_O_2_ (0.5 mmol L^−1^) was applied. To the 15 µL of sodium phosphate solution containing pDNA, SeO_3_^2−^, eventually SOD/CAT/H_2_O_2_, 5 µL of GSH was added to start the reaction. All stock solutions were prepared in ultrapure deionized water. The resulting samples were incubated for 30 min at 37°C. After incubation, the reaction mixtures were subjected to 0.6% agarose gel electrophoresis. Integrated densities of two pBR322 forms (supercoiled and nicked circular) in each lane were quantified using Image Studio analysis software (LI-COR Biotechnology, Bad Homburg, Germany) to estimate pDNA cleavage efficiency as a relative intensity (I_R_) of nicked circular band.

### Measurement of Arterial Pulse Wave

Adult male normotensive Wistar rats (*n* = 16; 320 ± 30 g) were purchased from the Department of toxicology and laboratory animals breeding, Centre of Experimental Medicine in Dobra Voda and housed at the Central Animal Housing Facility of Pavilion of Medical Sciences (registration number SK UCH 04022, Bratislava, Slovak Republic), where they received veterinary nursing care essentially as in our previous study [53]. Isoflurane (ISO) was used for inhalational anesthesia. Rat was put in an induction chamber flushed with 5 vol % ISO in 100% oxygen until loss of the righting reflex. The rat was then placed on heated pad (37 °C) and 3 vol % ISO in 100% oxygen (0.9 L per min) was further administered using a nose cone during all experiment. To prepare GSH/SeO_3_^2−^ mixture, 180 µl of 200 mmol L^–1^ GSH in sodium phosphate buffer (100 mmol L^−1^ sodium phosphate, 100 µmol L^−1^ DTPA, pH 7.4) and 60 µl of 100 mmol L^–1^ SeO_3_^2−^ in 0.9% NaCl were mixed (final pH ~ 4) and the solution was incubated in an microtube for 30 s or 90 s at 23 ± 1°C. After the incubation, the mixture was intravenously (IV) administered into the cannulated right jugular vein (500 µL kg^−1^) over a 15 s. The administration was repeated with new prepared and incubated mixture after 10–12 min. Theoretical initial concentration of GSH/SeO_3_^2−^ was calculated according to the rat weight as 75/12.5 in µmol kg^−1^. The left common carotid artery (*arteria carotis communis*) was cannulated to insert microcatheter pressure transducer FISO LS 2F (Harvard Apparatus, Holliston, MA, USA) for recording arterial pulse waveform (APW). Measurement of APWs and data evaluation were described in our previous studies [53, 54] and are detailed in the “Time-Dependent Effect of GSH/SeO_3_^2−^ on Rat APW-Ps” section. The calculation of hemodynamic parameters including units used is presented in Table S1.

### Measurement of Vasoactive Effect of GSH/SeO_3_^2−^

Adult female normotensive Wistar-Kyoto rats (*n* = 5; 198 ± 3 g) were obtained from an animal facility of the Institute of Normal and Pathological Physiology, Centre of Experimental Medicine, Slovak Academy of Sciences (approved user device no. SK UCH 03018, Bratislava, Slovak Republic). Rats were housed at 22–24°C on a 12:12 h dark–light cycle (lights on from 06.00 h until 18.00 h) and maintained on a standard pellet diet and tap water ad libitum. The rats were killed by decapitation after a brief anesthesia and the vascular segments from rats *a. mesenterica*, *a. femoralis* and thoracic aorta were dissected. Functional studies of the rat isolated arteries were performed as described in previous studies [53] with some modifications. Thoracic aorta was precontracted by noradrenaline (1 µmol L^−1^) and serotonin (10 µmol L^−1^), the femoral artery was precontracted by serotonin (1 µmol L^−1^) and mesenteric artery by noradrenaline (10 µmol L^−1^). After reaching the steady state of the contraction, GSH immediately followed by SeO_3_^2–^ were applied on the rings in the following concentrations: 250 µmol L^−1^ GSH + 20 µmol L^−1^ SeO_3_^2−^ (thoracic aorta *n* = 5, femoral *n* = 6 and mesenteric arteries *n* = 6) or 500 µmol L^−1^ GSH + 40 µmol L^−1^ SeO_3_^2−^ (thoracic aorta *n* = 5, femoral *n* = 9 and mesenteric arteries *n* = 6). The vasoactive responses were evaluated within the 10 min and strictly in the 10th min after the GSH/SeO_3_^2−^ application as a relative change in tension (%) of precontracted steady-state arteries. Hence, the reactions of arteries to GSH/SeO_3_^2−^ products were more complex during first 10 min, we found the inflection points that determined transient changes in the tension. After a washing-out and stabilization period (20 min.), the addition of the compounds was repeated again to see the reproducibility of the responses.

### Data analysis

Date analysis was performed with SigmaPlot 12.5 (Systat Software, San Jose, CA, USA) or GraphPad Prism 9 (GraphPad, La Jolla, CA, USA) software. Data are shown as the means ± standard deviation (SD), with *n* indicating the number of independent experiments. For between-group comparisons, one-way ANOVA with Dunnett’s post hoc test was used. For two-group comparison, two-tailed paired Student’s *t*-test was used. Results with *P* < 0.05 were considered statistically significant.

## Results

### UV–Vis Spectra of GSH/SeO_3_^2−^ at Different Molar Ratios and SeO_3_^2−^ Concentrations

**Fig. 1:**
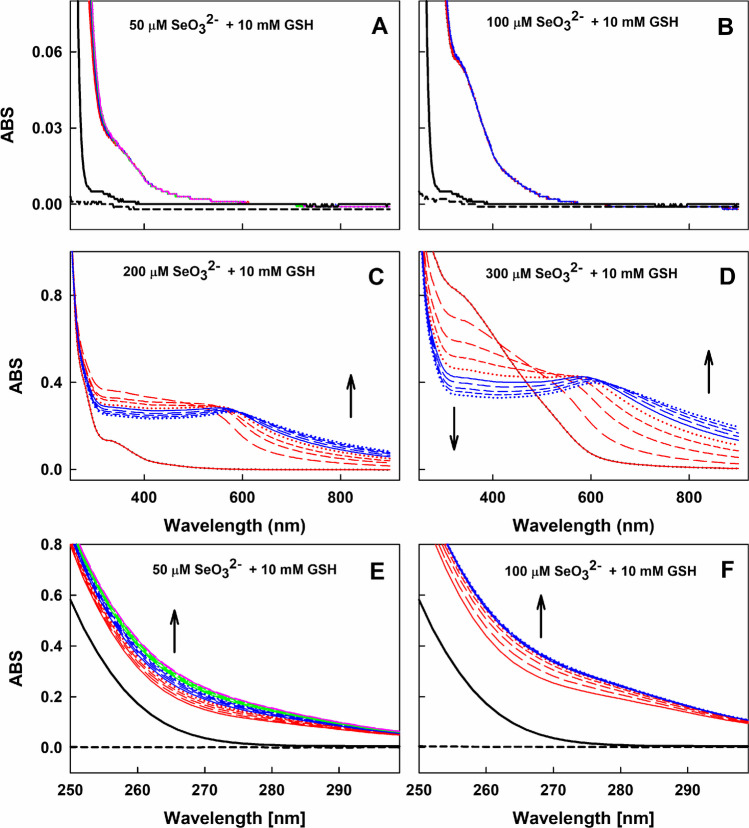
Time-resolved UV–Vis spectra of the interaction of GSH with SeO_3_^2−^ at different molar ratios. UV–Vis spectra of 50 (A) and 300 (B) µmol L^−1^ SeO_3_^2−^ (dash black). UV–Vis spectra of 10 mmol L^−1^ GSH (A and B, black). Time-resolved UV–Vis spectra of 10 mmol L^−1^ GSH with 50 (blue-green-pink, A), 100 (blue-red, B), 200 (C) and 300 (D) µmol L^−1^ SeO_3_^2−^. (E) Details of spectra (A) and (F) details of spectra (B). The spectra were measured every minute for 17 min (A and E) or 10 min (B, C, D and F) in 100 mmol L^−1^ sodium phosphate, 100 µmol L^−1^ DTPA (pH 7.4 at 37°C). The solid red line indicates the first spectrum measured 15 s after the addition of GSH/SeO_3_^2−^, which is followed each minute by: long dash red, medium dash red, short dash red, dotted red, solid blue line, long dash blue, medium dash blue, etc. (C, D, E and F). Arrows indicate the direction of ABS changes

It has been previously reported that thiol/selenite interactions have depended on many parameters [2, 17–21]. We also found that the time-resolved UV–Vis spectra of thiol/SeO_3_^2−^ mixture significantly depended on SeO_3_^2−^ concentration, thiol/SeO_3_^2−^ molar ratio, temperature or pH (Figs. 1, S1–S15). For example, when SeO_3_^2−^ concentration was low (50 or 100 µmol L^−1^) and the GSH/SeO_3_^2−^ molar ratios were high, 10,000/50 or 10,000/100 (in µmol L^−1^) (Fig. 1A,E and B,F respectively), UV–Vis spectrum (~ 300–900 nm) of the mixtures did not change for at least 10 min (Fig. 1A,B), but as shown in Fig. 1E and F, absorbance (ABS) increased at ~ 250–290 nm during the first ~ 15 or 6 min respectively, suggesting that observed reactions were completed until that time. ABS did not increase at the region > 400 nm indicating that colloidal elemental selenium Se^(0)^ was not formed [17, 19, 55, 56]. Therefore, it is supposed that the reactions at these conditions could be described by Eqs. (1), (2), and (4) (see “Discussion” section). At higher SeO_3_^2−^ concentrations and lower GSH/SeO_3_^2−^ molar ratios, 10,000/200 or 10,000/300 (in µmol L^−1^), UV–Vis spectra changed significantly in time (Fig. 1C and D, respectively), and ABS at > 600 nm increased in time indicating that colloidal Se^(0)^ was formed. Thus, it is supposed that reactions could be described by the above-mentioned Eqs. (1) and (2) including (3) and (5).

Significantly different time-resolved UV–Vis spectra observed at different GSH/SeO_3_^2−^ molar ratios and SeO_3_^2−^ concentrations (Figs. 1, S1–S5, [2, 19]) confirmed complex time-dependent GSH/SeO_3_^2−^ reactions leading to complicated explanation of our results. Therefore, properties of thiol/SeO_3_^2−^ mixtures to reduce the ^•^cPTIO stable radical were studied mostly at the molar ratios and SeO_3_^2−^ concentrations at which colloidal Se^(0)^ was not observed during the ^•^cPTIO reduction.

### Products of SeO_3_^2−^ Interacting with Excess of GSH or Cys Reduce the ^•^cPTIO Stable Radical

**Fig. 2:**
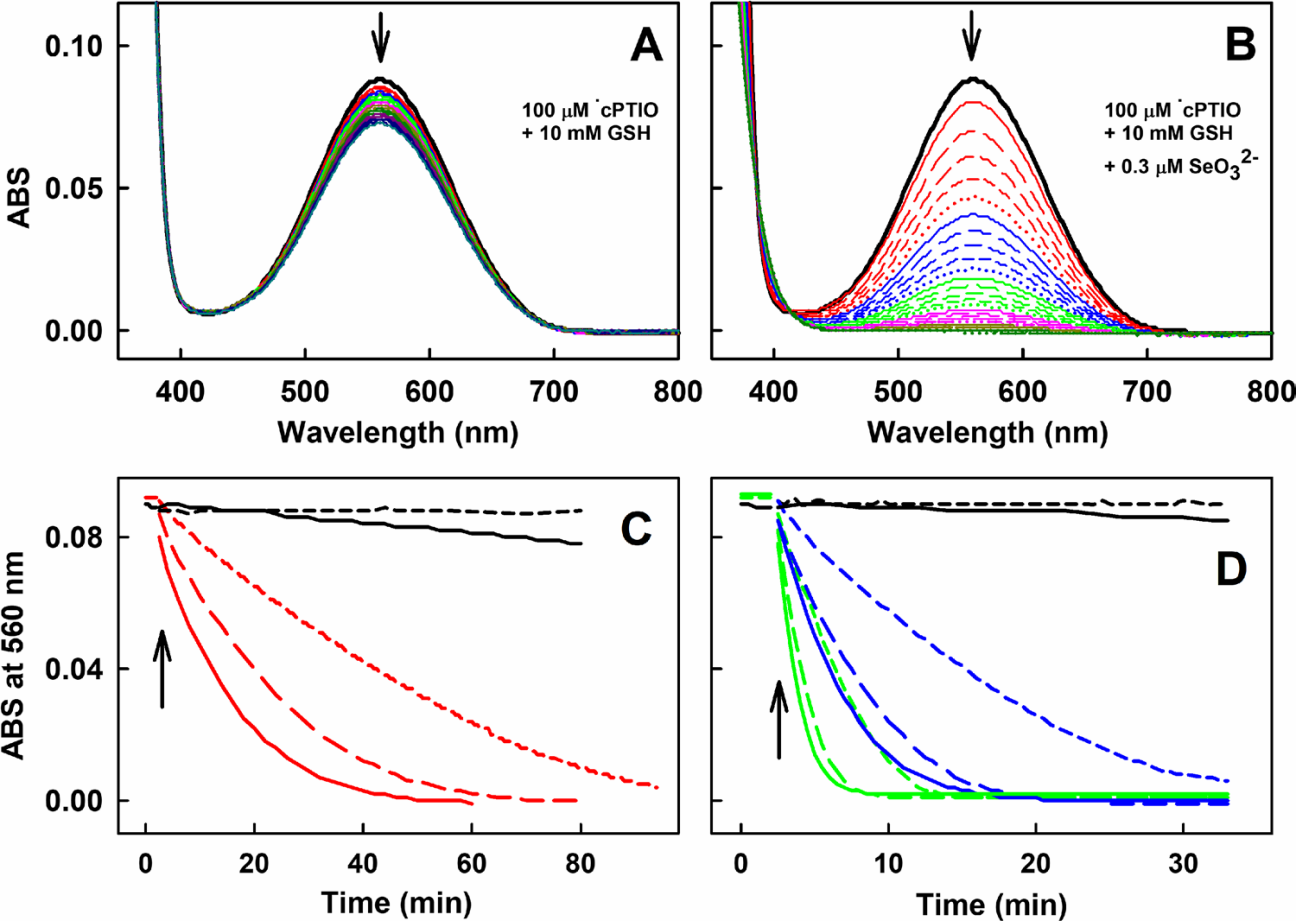
Representative time-resolved UV–Vis spectra of the interaction of GSH/SeO_3_^2–^/^•^cPTIO and kinetics of the reduction of ^•^cPTIO measured as the decrease of ABS at 560 nm during the interaction of SeO_3_^2−^ with GSH. (A) Spectra of ^•^cPTIO (100 µmol L^–1^) alone (black) and after the addition of GSH (10 mmol L^–1^). (B) Spectra of ^•^cPTIO (100 µmol L^–1^) alone (black) and after the addition of GSH (10 mmol L^–1^) and 300 nmol L^–1^ SeO_3_^2–^. The spectra were measured every 2 min for 60 min in sodium phosphate buffer (100 mmol L^–1^ sodium phosphate, 100 µmol L^–1^ DTPA, pH 7.4 at 37°C). The solid red line indicates the first spectrum measured 30 s after the addition of GSH (A) or GSH/SeO_3_^2−^ (B) into the ^•^cPTIO solution, which is followed each 2 min by: long dash red, medium dash red, short dash red, dotted red, solid blue line, long dash blue, medium dash blue, etc. (C) 100 µmol L^–1 •^cPTIO either with 100 µmol L^–1^ SeO_3_^2–^ (dash black) or 10 mmol L^–1^ GSH (black). Time-dependent effect of SeO_3_^2–^/^•^cPTIO (0.3/100 in µmol L^–1^) with 2 (short dash red), 5 (long dash red) and 10 mmol L^–1^ (red) GSH. (D) 100 µmol L^–1 •^cPTIO with 2 (dash black) and 10 mmol L^–1^ GSH (black). The spectra were measured at 1 min intervals. Time-dependent effect of SeO_3_^2–^/^•^cPTIO (1/100 in µmol L^–1^) with 2 (short dash blue), 5 (long dash blue) and 10 mmol L^–1^ (blue) GSH. Time-dependent effect of SeO_3_^2–^/^•^cPTIO (3/100 in µmol L^–1^) with 2 (short dash green), 5 (long dash green) and 10 mmol L^–1^ (green) GSH. The spectra were measured at 30 s intervals. Arrows mark the addition of SeO_3_^2−^ and GSH into ^•^cPTIO

Reduction of the ^•^cPTIO stable radical (ABS at 560 nm) was used as an in vitro model to compare the reducing properties of thiol/SeO_3_^2−^. At high thiol/SeO_3_^2−^ molar ratios, when Se^(0)^ was not formed, in control experiments, ^•^cPTIO alone was stable for at least 3 h. Besides, the presence of SeO_3_^2−^ up to a concentration of at least 100 µmol L^−1^ did not reduce this radical (Fig. 2). Since intracellular GSH concentration is in the range of 0.5–10 mmol L^–1^ [57], a similar range of GSH concentrations was used in this study. As detected by the time-resolved UV–Vis spectra (ABS at 560 nm), 10 mmol L^−1^ GSH had only a minor effect on reduction of 100 µmol L^−1 •^cPTIO (Fig. 2A). However, GSH in the presence of 300 nmol L^−1^ SeO_3_^2−^ time-dependently reduced this radical (Fig. 2B). No ABS at 410 nm or at > 700 nm was observed, indicating that Se^(0)^ was not formed. The rate of ^•^cPTIO reduction increased with the increasing concentration of GSH (Fig. 2C). Notably, the reduction increased when the GSH/SeO_3_^2−^ molar ratio of 6,666/1 increased to 16,666/1 and further to 33,333/1. The presence of SeO_3_^2−^ (0.3–3 µmol L^−1^) in the GSH solution significantly increased the ^•^cPTIO reduction in a concentration-dependent manner. It is shown that firstly SeO_3_^2−^/thiol reduced ^•^cPTIO and only then other interactions took place (Fig. S4). The reduction also increased in concentration-dependent manner of GSH (Fig. 2C and D). The reduction effect of GSH was similar when SeCl_4_ was used instead of SeO_3_^2−^ (Fig. S6), since selenium tetrachloride reacts with water to give selenous and hydrochloric acids. The reduction of ^•^cPTIO was negligible when GSH/SeO_4_^2−^ was used (Fig. S6).

When Cys was used instead of GSH, similar effects were observed. Cys (4 mmol L^−1^) had only a minor effect on reduction of 100 µmol L^−1 •^cPTIO. However, 2 or 4 mmol L^−1^ Cys in the presence of 200 nmol L^−1^ SeO_3_^2−^ time-dependently reduced ^•^cPTIO (Fig. S7A). To compare the potency of thiols to reduce ^•^cPTIO, the time-dependent effects were well-fitted by the equation of exponential decay: $f={ABS}_{0}+a\times {e}^{-b\times t}$, and calculated parameter ‘b’ was used for the comparison (Fig. S7B). Notably, the rate of the reduction increased when the Cys/SeO_3_^2−^ molar ratio of 10,000/1 increased to 20,000/1 (Fig. S7B, insert).

### Products of SeO_3_^2−^ Interacting with Excess of GSH, Cys or HCys Have Catalytic Properties to Reduce the ^•^cPTIO Stable Radical

**Fig. 3:**
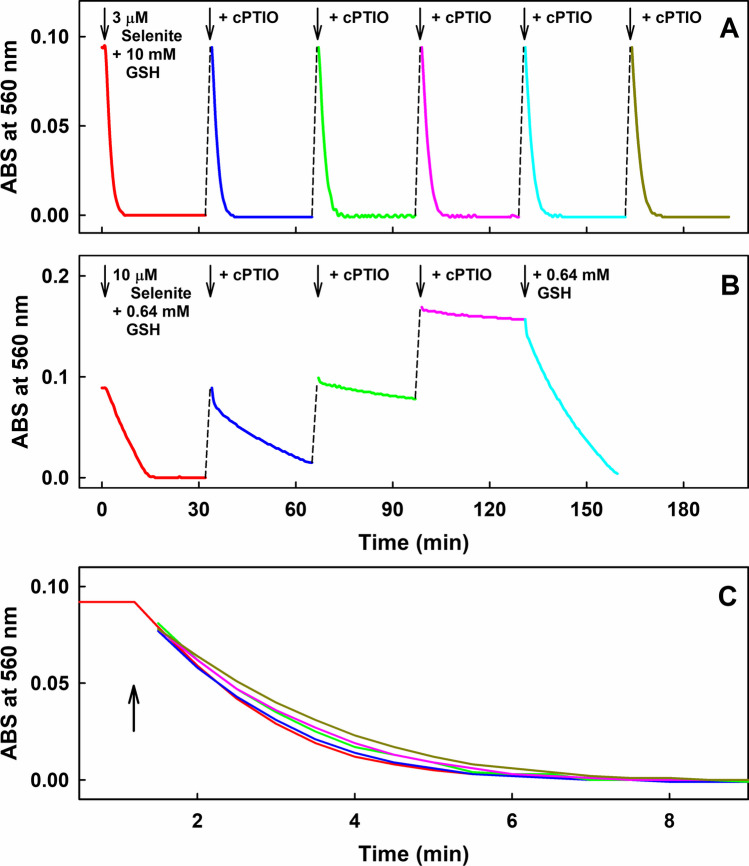
Time-dependent reduction of ^•^cPTIO measured as ABS at 560 nm of UV–Vis spectra during the interaction of SeO_3_^2−^ with GSH. (A) Reduction of 100 µmol L^−1 •^cPTIO with the mixture of GSH/SeO_3_^2−^ (10,000/3 in µmol L^–1^, red) and after adding of 100 µmol L^−1 •^cPTIO 5-times in a row (blue, green, pink, cyan and dark yellow). (B) Reduction of 100 µmol L^−1 •^cPTIO with the mixture of GSH/SeO_3_^2−^ (640/10 in µmol L^–1^) (red) and after adding of 100 µmol L^−1 •^cPTIO 3-times in a row (blue, green and pink), and after addition of 640 µmol L^–1^ GSH (cyan). (C) Details of the rate of decrease of ABS at 560 nm after 6-times addition of ^•^cPTIO into the GSH/SeO_3_^2−^ (10,000/3 in µmol L.^–1^) mixture. Data are from (A)

From the results that 300 or 200 nmol L^−1^ SeO_3_^2−^ in the presence of excess of GSH (Fig. 2) or Cys (Fig. S7), respectively, reduced 100 µmol L^−1 •^cPTIO, it follows that SeO_3_^2−^ participates in the catalytic reaction. To confirm catalytic properties of product(s) of GSH/SeO_3_^2−^ interaction and study its stability, an additional experiment was performed. We studied whether the GSH/SeO_3_^2−^ mixture at high molar ratio (10,000/3 in µmol L^–1^) was able to reduce ^•^cPTIO (Fig. 3A,C) repeatedly. After GSH/SeO_3_^2−^ had reduced ^•^cPTIO, new 100 µmol L^−1 •^cPTIO was added into the solution leading to reduction of the newly added ^•^cPTIO. Similar reduction rate and extend of ^•^cPTIO reduction was observed after adding of 100 µmol L^−1 •^cPTIO 6-times in a row (Fig. 3A and C). Similar catalytic properties were observed when L-Cys (Fig. S8), D-Cys (Fig. S9) or HCys (Fig. S10) were used instead of GSH at high thiol/SeO_3_^2−^ (10,000/3 in µmol L^−1^) molar ratios. However, when the molar ratio of GSH/SeO_3_^2−^ decreased to 64 (640/10 in µmol L^−1^) (Fig. 3B), the rate of ^•^cPTIO reduction was slower than in the case of 10,000/3 (in µmol L^−1^) molar ratio. After the second ^•^cPTIO addition, the rate significantly decreased and after the third and fourth addition, the rate of reduction was negligible. It is notable that subsequent addition of GSH (640 µmol L^−1^) reduced all ^•^cPTIO. Therefore, the results showed at Fig. 3B confirmed that a high excess of GSH is necessary for the catalytic properties and that the compound(s) involved in the reaction is (are) regenerating.

### Effects of AgNO_3_, Oxygen, SOD and Na_2_Se on Reduction of ^•^cPTIO

**Fig. 4:**
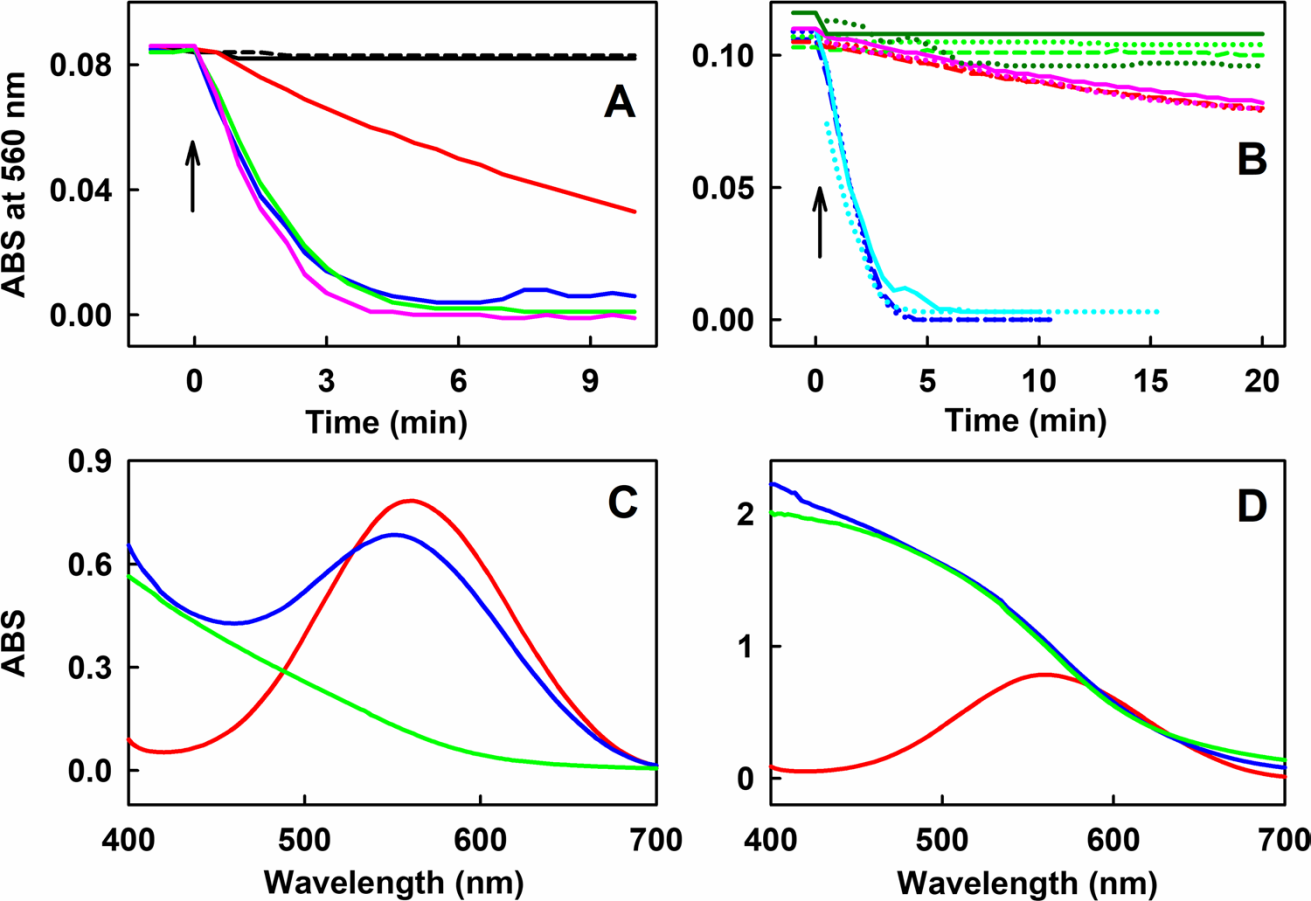
Time-dependent reduction of 100 µmol L^–1 •^cPTIO measured as ABS at 560 nm of UV–Vis spectra during the interaction of SeO_3_^2−^ with GSH in the absence and presence of AgNO_3_ (A) or bubbled by argon (B). (A) Stock solution containing 50 mmol L^–1^ GSH and 50 µmol L^–1^ SeO_3_^2−^ was prepared by dissolving powder of GSH in 100 µL of 50 µmol L^–1^ SeO_3_^2−^ in the phosphate buffer (pH 8.5 at 37°C; final pH ~ 7.2). Aliquot (100 µL) of the stock solution incubated at 37°C for 40–60 min was added into the 900 µL of sodium phosphate buffer (100 mmol L^−1^ sodium phosphate, 100 µmol L^−1^ DTPA, pH 7.4 at 37°C) and UV–Vis spectra were measured at 30 s intervals at 37°C for 10 min. Final sample contained (100 µmol L^–1 •^cPTIO, 5 µmol L^–1^ SeO_3_^2−^, 5 mmol L^−1^ GSH) and 0 (pink), 2.5 (green), 5 (blue), 7.5 (red), 10 (dash black) and 15 µmol L^–1^ AgNO_3_ (black). (B) Time-dependent reduction of 100 µmol L^–1 •^cPTIO measured as ABS at 560 nm of UV–Vis spectra during the interaction of 10 µmol L^–1^ SeO_3_^2−^ with 40, 200 and 2000 µmol L^–1^ GSH under standard air atmosphere (std. ATM) and when solutions were bubbled with argon for 10 min. Samples under std. ATM: 40/10 (in µmol L^–1^, *n* = 2, dotted and dash green), 200/10 (in µmol L^–1^, *n* = 2, dotted and dash red) and 2000/10 GSH/SeO_3_^2−^ (in µmol L^–1^, *n* = 2, dotted and dash blue). Samples bubbled with argon: 40/10 (in µmol L^–1^, *n* = 2, dotted and dash dark green), 200/10 (in µmol L^–1^, *n* = 2, dotted and dash pink) and 2000/10 GSH/SeO_3_^2−^ (in µmol L^–1^, *n* = 2, dotted and dash cyan). (C and D) UV–Vis spectrum of 850 µmol L^–1 •^cPTIO in the phosphate buffer (red) and the UV–Vis spectrum after the phosphate buffer containing ^•^cPTIO (850 µmol L^–1^) was added to Na_2_Se powder (≤ 0.1 mg, c) or (≤ 0.2 mg, D) and measured 4 s after the addition (blue). UV–Vis spectrum measured 4s after addition of the phosphate buffer to powder Na_2_Se (≤ 0.1 mg, C) or (≤ 0.2 mg, D) (green). Buffer: 100 mmol L^–1^ sodium phosphate, 100 µmol L^–1^ DTPA, pH 7.4, 37°C

One of the leading species after reaction of SeO_3_^2−^ with thiols is H_2_Se (HSe^–^, Se^2–^). It is a highly reactive ion that reacts with Ag forming Ag_2_Se precipitate [58, 59]. Therefore, to confirm the possible involvement of H_2_Se in the ^•^cPTIO reduction, the effect of AgNO_3_ on the reduction was studied, to determine whether Ag can stop ^**•**^cPTIO reduction mediated by the SeO_3_^2−^/thiols mixture. Since we observed that Ag formed precipitate with SeO_3_^2−^, the following approach was used. A stock solution of GSH/SeO_3_^2−^ (50 mmol L^−1^/50 µmol L^−1^) was incubated at 37°C for 40–60 min, and then an aliquot was added to 100 µmol L^−1**•**^cPTIO solution without or with AgNO_3_ (final ^**•**^cPTIO/GSH/SeO_3_^2−^ concentrations were 100/5000/5 in µmol L^−1^). The obtained results pointed that the highest concentrations of AgNO_3_ tested (10 or 15 µmol L^−1^) totally inhibited ^**•**^cPTIO reduction, whereas the lowest ones (2.5 and 5 µmol L^−1^) were not able to trigger the inhibition effect. Additionally, AgNO_3_ exerted a partial inhibition at 7.5 µmol L^−1^ concentration (Fig. 4A). Since AgNO_3_, at molar Ag/SeO_3_^2−^ ratios 0, 0.5 and 1 had no effect, at 1.5 decreased and at higher molar ratios (2 and 2.5) completely stopped the ^**•**^cPTIO reduction, we suggest that Ag interacted with H_2_Se forming Ag_2_Se. The results suggest that H_2_Se is involved in the reduction of ^**•**^cPTIO. However, other negatively charged species generated in the reaction cannot be excluded.

To study whether oxygen plays a role in the ^**•**^cPTIO reduction, stock solutions were vigorously bubbled with argon and compared with the solutions under standard atmosphere. Three different GSH/SeO_3_^2−^ molar ratios (40/10, 200/10 and 2000/10 in µmol L^−1^) were used. Notably, oxygen had not significant effect on ^**•**^cPTIO reduction (Fig. 4B).

We studied whether H_2_Se/HSe^–^ plays a role in the ^**•**^cPTIO reduction. A stock solution of 10 mmol L^–1^ Na_2_Se alone or with ^**•**^cPTIO in phosphate buffer changed color to orange-red in 2–5 s after dissolving due to oxidation of H_2_Se to Se^(0)^ (Fig. S11E). Therefore, we chose the procedure of adding the ^**•**^cPTIO solution to small amount of Na_2_Se powder and measured UV–Vis spectra in 4 s. When 850 µmol L^–1 •^cPTIO in the phosphate buffer was added to a small amount of powdered Na_2_Se (≤ 0.1 mg) in cuvette and UV–Vis spectra were measured promptly in 4 s, a decrease of ^**•**^cPTIO ABS at 560 nm was observed (Fig. 4C) indicating its reduction. The reduction of ^**•**^cPTIO was more pronounced when higher amount of powder Na_2_Se (≤ 0.2 mg) was used (Fig. 4D). The results confirmed that H_2_Se/HSe^–^ is involved in the ^**•**^cPTIO reduction.

**Fig. 5:**
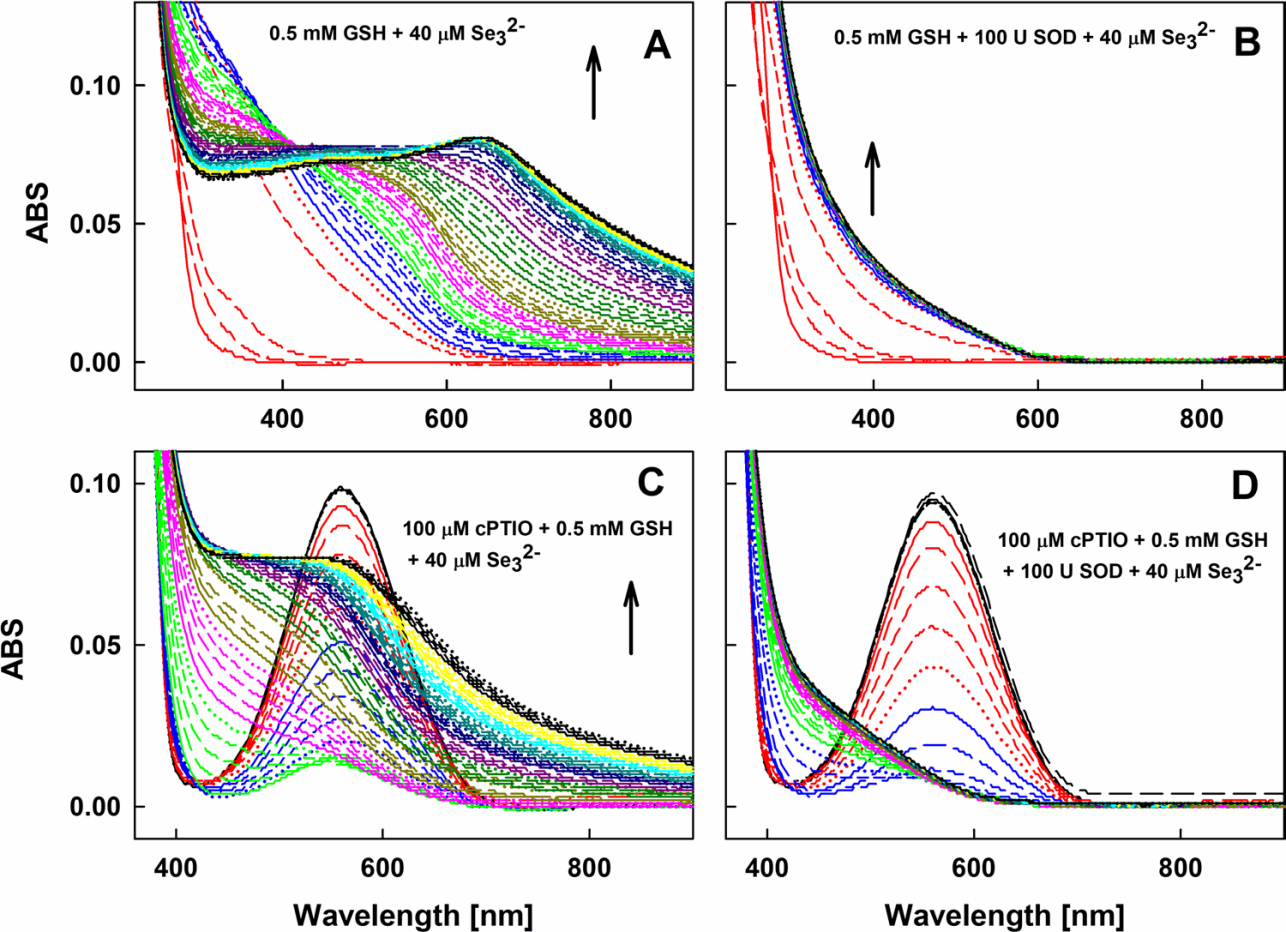
Time-resolved UV–Vis spectra of the interaction of GSH/SeO_3_^2–^/^•^cPTIO without and with SOD. The UV–Vis spectra of GSH/SeO_3_^2–^ (500/40 in µmol L^–1^) without (A) and with 100 Units of SOD (B). The UV–Vis spectra of^•^cPTIO/GSH/SeO_3_^2–^ (100/500/40 in µmol L^–1^) without (C) and with 100 Units of SOD (D). The spectra were measured every 30 s for 60 min in sodium phosphate buffer (100 mmol L^–1^ sodium phosphate, 100 µmol L^–1^ DTPA, pH 7.4 at 37°C). The solid red line indicates the first spectrum measured 30 s after the addition of SeO_3_^2−^ into GSH or GSH/SOD solution, which is followed each 30 s by: long dash red, medium dash red, short dash red, dotted red, solid blue line, long dash blue, medium dash blue, etc.

Since ^•^O_2_^−^ is produced during the interaction of SeO_3_^2−^ with GSH (Eq. (14)), we studied if ^•^O_2_^−^ is involved in the ^•^cPTIO reduction. The time-resolved spectra of GSH/SeO_3_^2–^ changed gradually for about 30 min with final ABS broad peak at ~ 660 nm (Fig. 5A). The results indicate that among several products, colloidal Se^(0)^ and/or unspecified Se-nanoparticles were formed [17, 19, 55, 56]. However, when GSH/SeO_3_^2–^ contained SOD, chemical reaction was taking place for 2.5 min only and after that UV–Vis spectra did not change. There was no increased ABS at ≥ 600 nm indicating that that the colloidal Se-containing particles were not formed (Figs. 5B and S11). The GSH/SeO_3_^2–^ mixture reduced ^•^cPTIO and after that the spectra changed gradually for about 30 min (Fig. 5C). In contrast to Fig. 5A, the final ABS had a broad 400–900 nm range (Fig. 5C), but the peak at 660 nm was not observed, indicating different end products of the reactions. However, in the presence of SOD, GSH/SeO_3_^2–^ time-dependently reduced ^•^cPTIO (Figs. 5D and S12) and ABS did not increase at ≥ 600 nm, similarly as shown in Fig. 5B. The results indicate that ^•^O_2_^−^ did not significantly influence reduction of ^•^cPTIO by GSH/SeO_3_^2–^, but it significantly reduced formation of colloidal Se-containing particles.

### Stability of Product(s) of GSH/SeO_3_^2−^ Mixture to Reduce the ^•^cPTIO

**Fig. 6:**
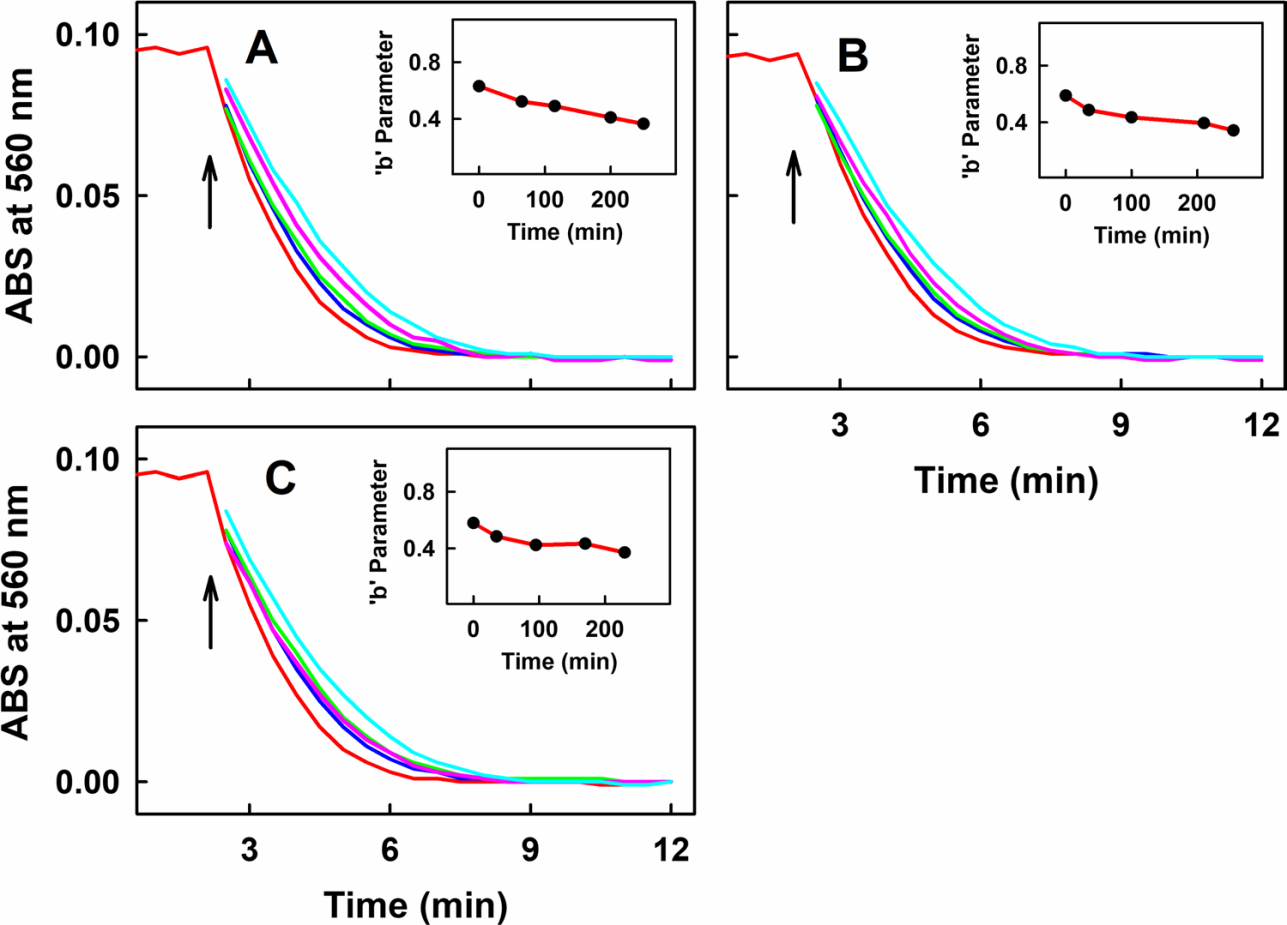
Time-dependent reduction of ^•^cPTIO measured as ABS at 560 nm of UV–Vis spectra during the interaction of SeO_3_^2–^ with GSH. Stock solution containing 50 mmol L^–1^ GSH and 50 µmol L^–1^ SeO_3_^2–^was prepared by dissolving GSH (1.54 mg) in 100 µL of 50 µmol L^–1^ SeO_3_^2–^ in the 100 mmol L^–1^ phosphate buffer (8.5 pH at 37°C). Final pH of GSH/ SeO_3_^2–^ was ~ 7.0. Aliquot (100 µL) of the stock solution at 1 (A), 13 (B) and 40 min (C) after the preparation was added into the 900 µL of the 100 mmol L^–1^ sodium phosphate, 100 µmol L^–1^ DTPA, pH 7.4, 37°C buffer (final 5 mmol L^–1^ GSH and 5 µmol L^–1^ SeO_3_^2–^) and UV–Vis spectra were measured at 30 s intervals. (A) Time-dependent reduction of 100 µmol L^–1 •^cPTIO by the mixture of GSH/SeO_3_^2–^ (final 5000/5 in µmol L^–1^, stock solution incubated 1 min, red) and subsequent adding the 100 µmol L^–1 •^cPTIO four times at 65 (blue), 115 (green), 200 (pink) and 250 min (cyan). (B) Time-dependent reduction of 100 µmol L^–1 •^cPTIO by the mixture of GSH/SeO_3_^2–^ (final 5000/5 in µmol L^–1^, stock solution incubated 13 min, red) and adding the 100 µmol L^–1 •^cPTIO four times at 35 (blue), 100 (green), 210 (pink) and 255 min (cyan). (C) Time-dependent effect of 100 µmol L^–1 •^cPTIO with the mixture of GSH/SeO_3_^2–^ (final 5000/5 in µmol L^–1^, stock solution incubated 40 min, red) and adding the 100 µmol L^–1 •^cPTIO four times at 35 (blue), 95 (green), 170 (pink) and 230 min (cyan). Inserts: Ordinal (time) dependence of parameter ‘b’ calculated from the fitted time-dependent data (A, B, C) using equation of exponential decay: $f={ABS}_{0}+a\times {e}^{-b\times t}$, where ABS_0_ is ABS at initial time, *t* is time (min), ‘*a*’ (arbitrary units) and ‘*b*’ (min^–1^) are estimated parameters

Next, the stability and the potency of the active products of GSH/SeO_3_^2−^ stock solution were studied. When the stock solution of 50 mmol L^−1^ GSH with 50 µmol L^−1^ SeO_3_^2−^ was prepared by dissolving GSH powder in 100 µL of 50 µmol L^−1^ SeO_3_^2−^ in phosphate buffer with pH 8.5 (final pH ~ 7.0), products of GSH/SeO_3_^2−^ interaction were similarly effective in reducing ^•^cPTIO after incubating the stock solution for 1, 13 or 40 min at 37°C (Fig. 6). According to parameter “b” of exponential decay, catalytic properties of the incubated stock solutions were similar. This fact suggests that the active product(s) in GSH/SeO_3_^2−^ stock solution are stable. When GSH/SeO_3_^2−^ stock solutions were kept at 23°C or at − 20°C for 24 h, ^•^cPTIO was still fully reduced, but the rates of the reduction partially decreased indicating relative stability of the active products (Fig. S13A and B, respectively). The results may indicate that active products may include GSSeSG and/or GSSeH formed by Eqs. (1) and (2).

The rate of the ^•^cPTIO reduction and stability of GSH/SeO_3_^2−^ active species was significantly pH-dependent within the studied range of pH 6.5–8.5 (Fig. S14). The lowest rate and stability of the species were observed at pH 6.5. Both, rate and stability significantly increased with the increasing pH, being the highest ones observed at pH 8.5. When the final pH of incubated GSH/SeO_3_^2−^ mixture was ~ 4.5, rates of the ^•^cPTIO reduction decreased with incubation time, but notably it increased after incubation in measured UV–Vis cuvette (pH 7.4, 37°C) (Fig. S15). The results suggest that the active substances could include GSSe^–^, HSe^–^ and/or Se^2–^, as described by Eqs. (1) and (2).

It was of interest to know whether the phosphate buffer is specific for the ^•^cPTIO reduction. Therefore, 100 mmol L^−1^ Tris–HCl buffer (pH 7.4 at 37°C) was used as reaction medium. The repeated addition of GSH/SeO_3_^2−^ (5000/5 in µmol L^−1^) into the Tris–HCl buffer effectively reduced 100 µmol L^−1 •^cPTIO (Fig. S15D). Rate and extent of the ^•^cPTIO reduction were approximately similar to those ones observed in the phosphate buffer (Fig. S14B), and they slowly decreased in time.

To test whether GSH/SeO_3_^2−^ active species can reduce other radicals, stable radical TEMPO was used. GSH (10 mmol L^−1^) had only a minor effect on radical reduction. However, SeO_3_^2−^ together with GSH time-dependently reduced TEMPO (Fig. S16).

### Comparison of the Mixtures of GSH and Cys with Increasing Concentration of SeO_3_^2−^ to Reduce ^•^cPTIO

**Fig. 7:**
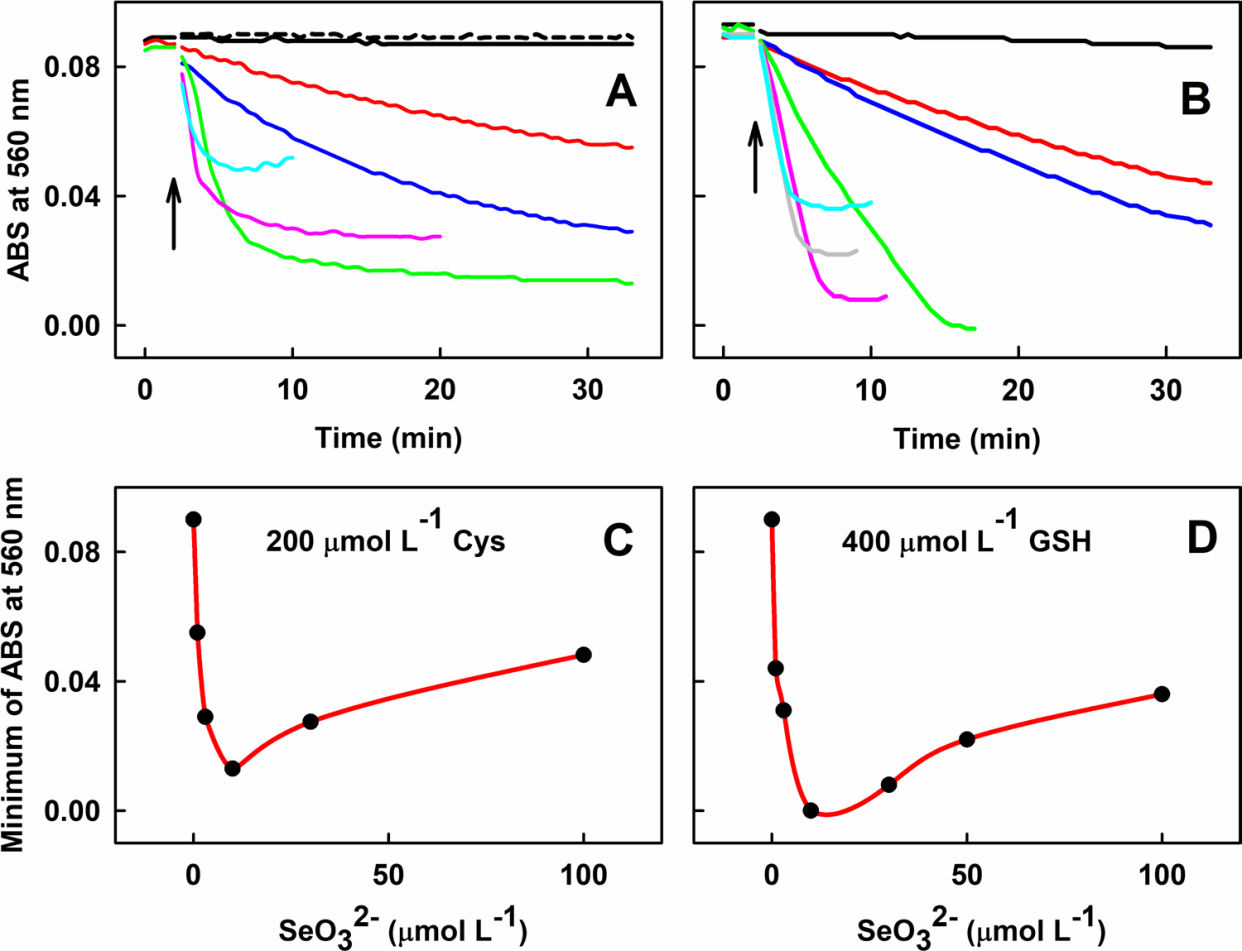
Time-dependent reduction of ^•^cPTIO measured as ABS at 560 nm of UV–Vis spectra during the interaction of SeO_3_^2−^ with Cys or with GSH. (A) 100 µmol L^–1 •^cPTIO either with 50 µmol L^–1^ SeO_3_^2–^ (dash black) or with 200 µmol L^–1^ Cys (black). Time-dependent effect of Cys/^•^cPTIO (200/100 in µmol L^–1^) with 1 (red), 3 (blue), 10 (green), 30 (pink) and 100 (cyan) µmol L^–1^ SeO_3_^2–^. (B) 100 µmol L^–1 •^cPTIO with 400 µmol L^–1^ GSH (black). Time-dependent effect of GSH/^•^cPTIO (400/100 in µmol L^–1^) with 1 (red), 3 (blue), 10 (green), 30 (pink), 50 (gray) and 100 (cyan) µmol L^–1^ SeO_3_^2–^. Arrows mark the addition of SeO_3_^2−^ with Cys or with GSH into.^•^cPTIO. Minimum of ABS at 560 nm values found in the range of 0–30 min; (C) Data taken from (A), (D) data taken from (B)

The effects of GSH (400 µmol L^–1^) or Cys (200 µmol L^–1^) at increasing concentrations of SeO_3_^2−^ were compared (Fig. 7). In the control experiments, 200 µmol L^–1^ Cys did not reduce the ^•^cPTIO radical. In the presence of thiols, SeO_3_^2–^ had a biphasic concentration-dependent effect on the extent of ^•^cPTIO reduction. The presence of SeO_3_^2–^ (1–10 µmol L^–1^) in the Cys solution significantly increased the ^•^cPTIO reduction in a concentration-dependent manner (Fig. 7A and C). Even faster ^•^cPTIO reduction rates were obtained when 400 µmol L^–1^ Cys was used (Fig. S17). At higher SeO_3_^2−^ concentrations (30–100 µmol L^–1^), the reduction rate increased within the first seconds, but the total extent of the ^•^cPTIO reduction then decreased in latter time intervals (Fig. 7A and C; Fig. S18). A similar biphasic concentration-dependent effect of SeO_3_^2–^ on ^•^cPTIO reduction was observed when it interacted with 400 µmol L^–1^ GSH (Fig. 7B and D). In control experiments, 400 µmol L^–1^ GSH did not reduce ^•^cPTIO. However, the presence of SeO_3_^2–^ (1–10 µmol L^–1^) in the GSH solution significantly increased the ^•^cPTIO reduction in a concentration-dependent manner. At higher SeO_3_^2−^ concentrations (30–100 µmol L^–1^), the reduction rate was high in the first seconds, but the total extent of the ^•^cPTIO reduction later decreased (Fig. 7B and D).

### Comparison of the Mixtures of Different Thiols with SeO_3_^2−^ or with SeMet to Reduce ^•^cPTIO

**Fig. 8:**
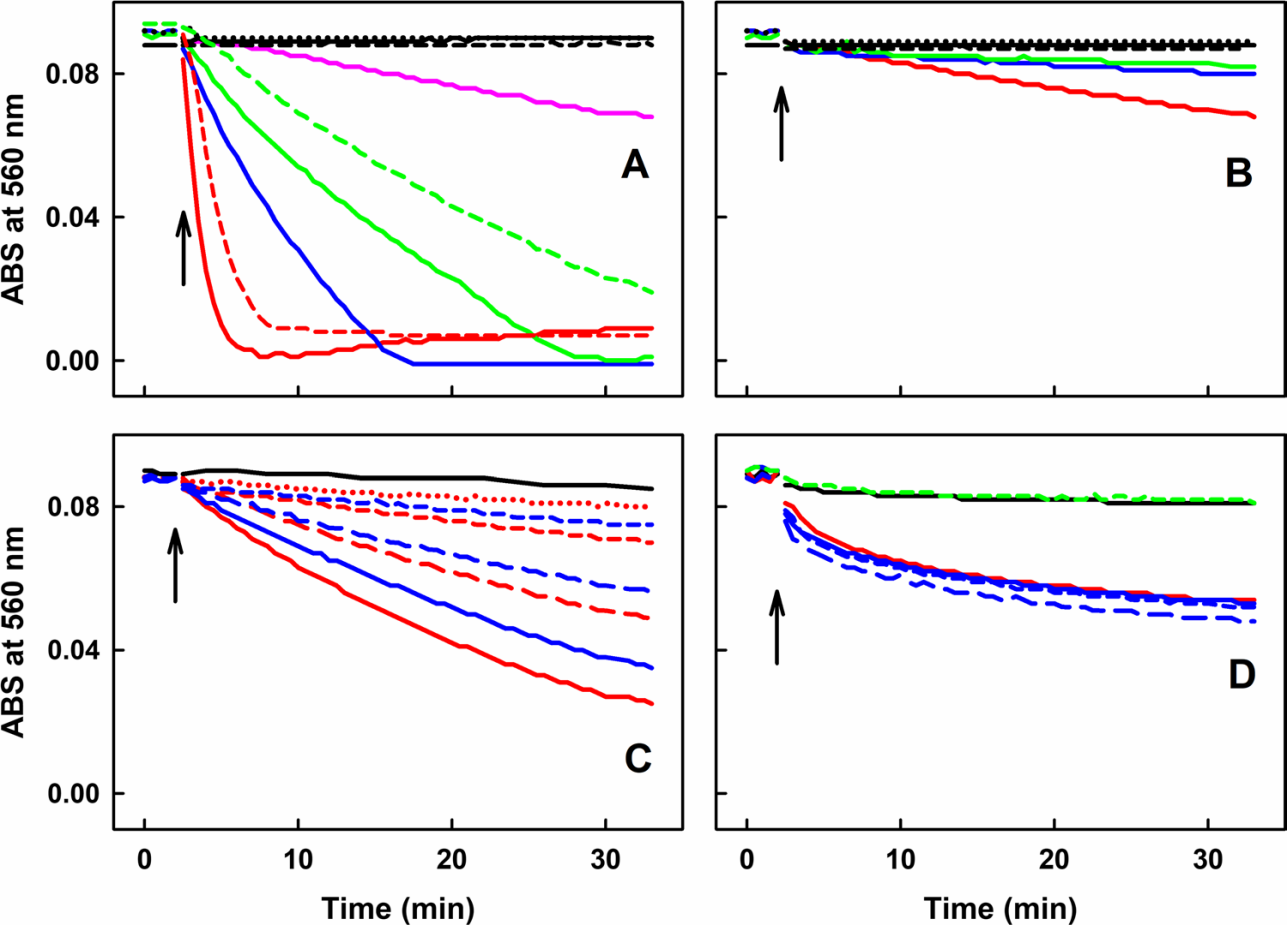
Time-dependent reduction of ^•^cPTIO measured as ABS at 560 nm of UV–Vis spectra during the interaction of SeO_3_^2−^ or SeMet with the thiol-based compounds. (A) SeO_3_^2−^/^•^cPTIO (10/100 in µmol L^−1^) with 400 µmol L^−1^ of the compounds: Cys (red), HCys (blue), GSH (green), NAC (pink), cystine (black), GSSG (dash black) and MET (dotted black), measured at 37°C. SeO_3_^2−^/^•^cPTIO (10/100 in µmol L^−1^) with 400 µmol L^−1^ of Cys (dash red) and GSH (dash green) measured at 23°C. (B) SeMet/^•^cPTIO (200/100 in µmol L^−1^) with 400 µmol L^−1^ of the compounds: Cys (red), HCys (blue), GSH (green), cystine (black), GSSG (dash black) and MET (dotted black). (C) GSH/^•^cPTIO (5000/100 in µmol L^–1^) with 30 (short dash blue), 100 (long dash blue) and 200 (blue) µmol L^–1^ SeMet. GSH/^•^cPTIO (10 000/100 in µmol L^–1^) with 10 (dotted red), 30 (short dash red), 100 (long dash red) and 200 (red) µmol L^–1^ SeMet. (D) Trolox/^•^cPTIO (10/100 in µmol L^–1^) without (dash green) and with 10 µmol L^−1^ SeO_3_^2−^ (black). Trolox/^•^cPTIO (100/100 in µmol L^–1^) without (red) and with 10 (short dash blue), 30 (long dash blue) and 100 (blue) µmol L^−1^ SeO_3_^2−^. Arrows mark the addition of SeO_3_^2−^ and compounds into ^•^cPTIO

The effects of different thiol-based compounds at a final concentration of 400 µmol L^–1^ interacting with 10 µmol L^–1^ SeO_3_^2−^ on the reduction of 100 µmol L^–1 •^cPTIO was compared (Fig. 8A). At a constant concentration of 10 µmol L^–1^ SeO_3_^2−^, the order of potency to reduce ^•^cPTIO was Cys > HCys > GSH > NAC, whereas compounds with no free thiol group (cystine, GSSG and Met) had no effect. The same order of the potency was observed when the compounds at 200 µmol L^–1^ concentration were used (Fig. S19). The rate of the SeO_3_^2−^/Cys or SeO_3_^2−^/GSH mixtures to reduce ^•^cPTIO decreased at 23°C in comparison to 37°C (Fig. 8A).

The effect of 200 µmol L^–1^ SeMet (another form of Se) interacting with the thiol-based compounds (400 µmol L^–1^) on reduction of ^•^cPTIO was also examined for comparison (Fig. 8B). In this case, the order of potency to reduce ^•^cPTIO was as follows: Cys > HCys ~ GSH > NAC, whereas cystine, GSSG and Met had still no effect. The order of the potency was the same as for SeO_3_^2−^, but the effect of SeMet was significantly lower than that of SeO_3_^2−^. However, in the presence of 5–10 mmol L^−1^ GSH, SeMet (10–200 µmol L^−1^) in a concentration-dependent manner significantly reduced ^•^cPTIO (Fig. 8C).

Furthermore, it was of interest to know whether SeO_3_^2−^ or GSH can potentiate the reducing effect of Trolox, a water soluble derivative of α-tocopherol. At 10 or 100 µmol L^−1^ concentrations, Trolox had a minor effect on ^•^cPTIO reduction and its effect was not potentiated by the presence of 10, 30 or 100 µmol L^−1^ SeO_3_^2−^ (Fig. 8D). Similarly, 400 µmol L^−1^ or 5 mmol L^−1^ GSH did not potentiate the ^•^cPTIO reduction in the presence of 30 and 100 µmol L^−1^ or 5 µmol L^−1^ Trolox, respectively (Fig. S20).

### No Spin Adducts of BMPO in the Presence of GSH/SeO_3_^2–^ Were Observed

**Fig. 9:**
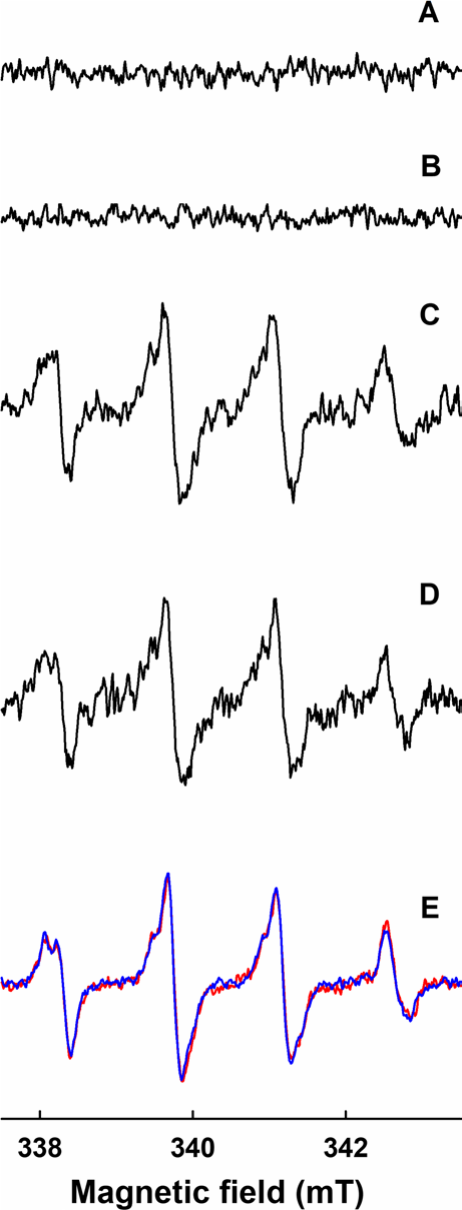
Representative EPR spectra of 30 mmol L^−1^ BMPO in the presence of (A) GSH/SeO_3_^2–^ (400/10 µmol L^−1^), (B) 4 mmol L^−1^ ferricyanide, (C) GSH/SeO_3_^2–^/ferricyanide (400/10/4000 µmol L^−1^), (D) GSH/ferricyanide (400/4000 in µM), (E) GSH/SeO_3_^2–^/ferricyanide (2000/10/4000 µmol L^−1^) as red line and GSH/ferricyanide (2000/4000 µmol L^−1^) as blue line. Samples were measured 5 times for 2 min at 24°C

Since SOD significantly reduced the formation of Se-containing colloidal particles (Fig. 5), indicating that the superoxide radical was formed during the GSH/SeO_3_^2–^ interaction, we aimed to detect superoxide formation using BMPO spin trap EPR spectroscopy. In control spin trap experiments, BMPO did not create EPR signal in the buffer solution during a 15-min long observation. Moreover, no spin adducts of BMPO were observed in the presence of different concentrations and molar ratios of GSH/SeO_3_^2–^ (1000/1000, 1000/20, 1000/5, 500/40, 400/10, 100/10 µmol L^–1^) incubated either for 10 s or 60–600 s at 24 or 37°C (Fig. 9). In order to rule out that GSH/SeO_3_^2–^ could also reduce BMPO-adducts and thus diminished EPR signal, an oxidant ferricyanide was used [51]. After addition of ferricyanide into the samples containing BMPO/GSH/SeO_3_^2–^, EPR spectra were seen. However, similar spectra were observed in the samples containing only BMPO/GSH. The results showed that ferricyanide oxidized GSH to GS^•^ which is trapped by BMPO forming BMPO-GS^•^ radical as it was published [60]. Therefore, the capture of superoxide or other radicals by the BMPO spin trap cannot be confirmed in this study. However, it does not mean that superoxide anion or other radicals could not be formed.

### Thiols Interacting with SeO_3_^2−^ Induce pDNA Cleavage

**Fig. 10:**
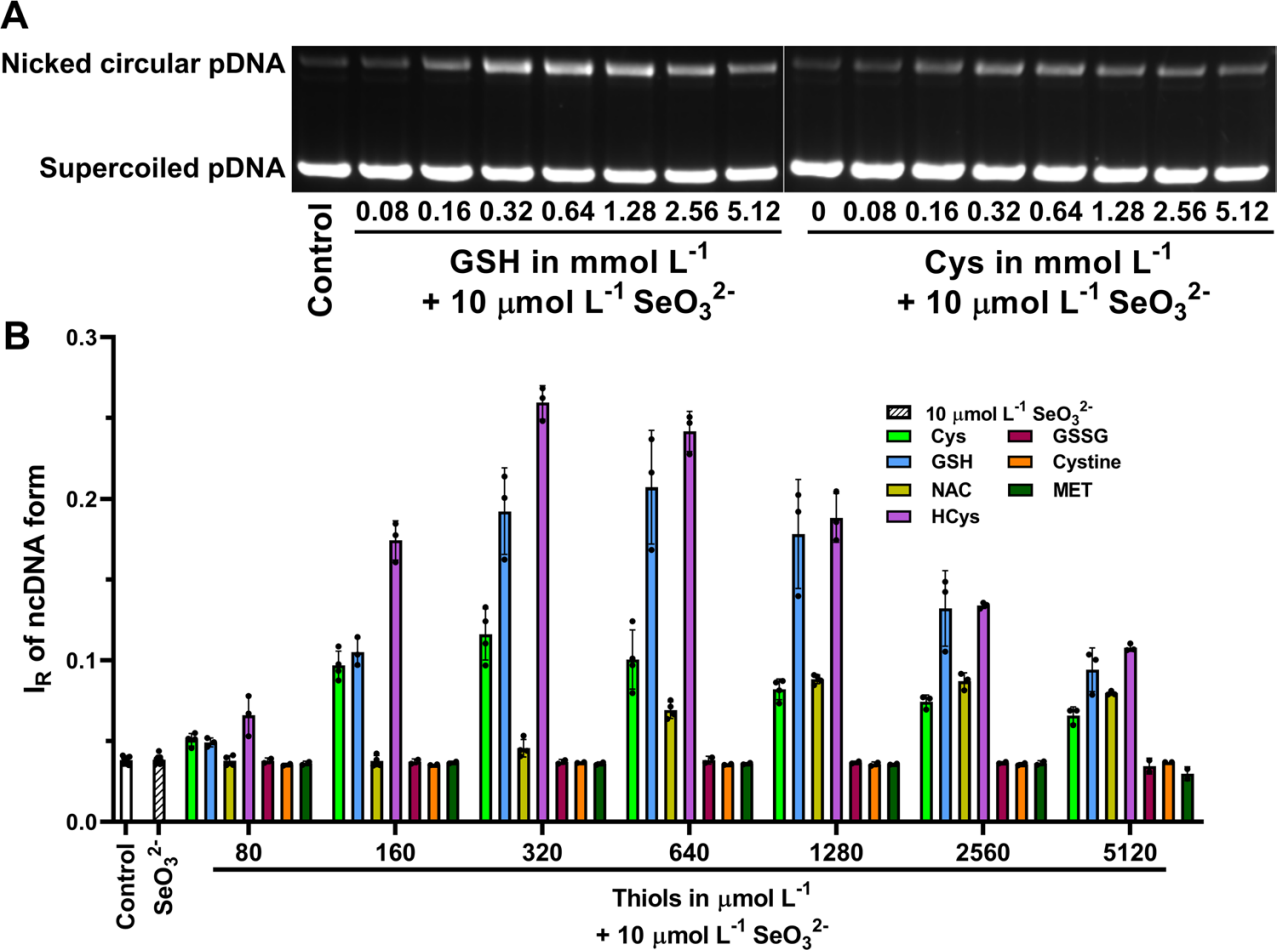
The pDNA (4 361 bp) cleavage potency of SeO_3_^2−^ in the presence of thiols. Representative gels indicating the effects of increasing concentrations of GSH or Cys (0–5.12 mmol L^−1^) on pDNA cleavage by 10 µmol L^−1^ SeO_3_^2−^ (A). The bands at the bottom and top correspond to the circular supercoiled and nicked forms of pDNA, respectively. The final concentration of pDNA was 0.2 µg in 20 µL in a 25 mmol L^−1^ sodium phosphate buffer and 50 µmol L^−1^ DTPA at 37°C. The graphical representation of the pDNA cleavage by 10 µmol L^−1^ SeO_3_^2−^ in the presence of increasing concentrations of thiols (B). Control is without any treatment. I_R_ of ncDNA form represents the relative intensity of the nicked circular pDNA. Data represent values from individual samples (*n* ≥ 3 for Cys, GSH, NAC, HCys and *n* = 2 for the rest of thiols) in a 25 mmol L^−1^ sodium phosphate buffer and 50 µmol L^−1^ DTPA at 37°C. Horizontal black marks indicate means ± SD

**Fig. 11:**
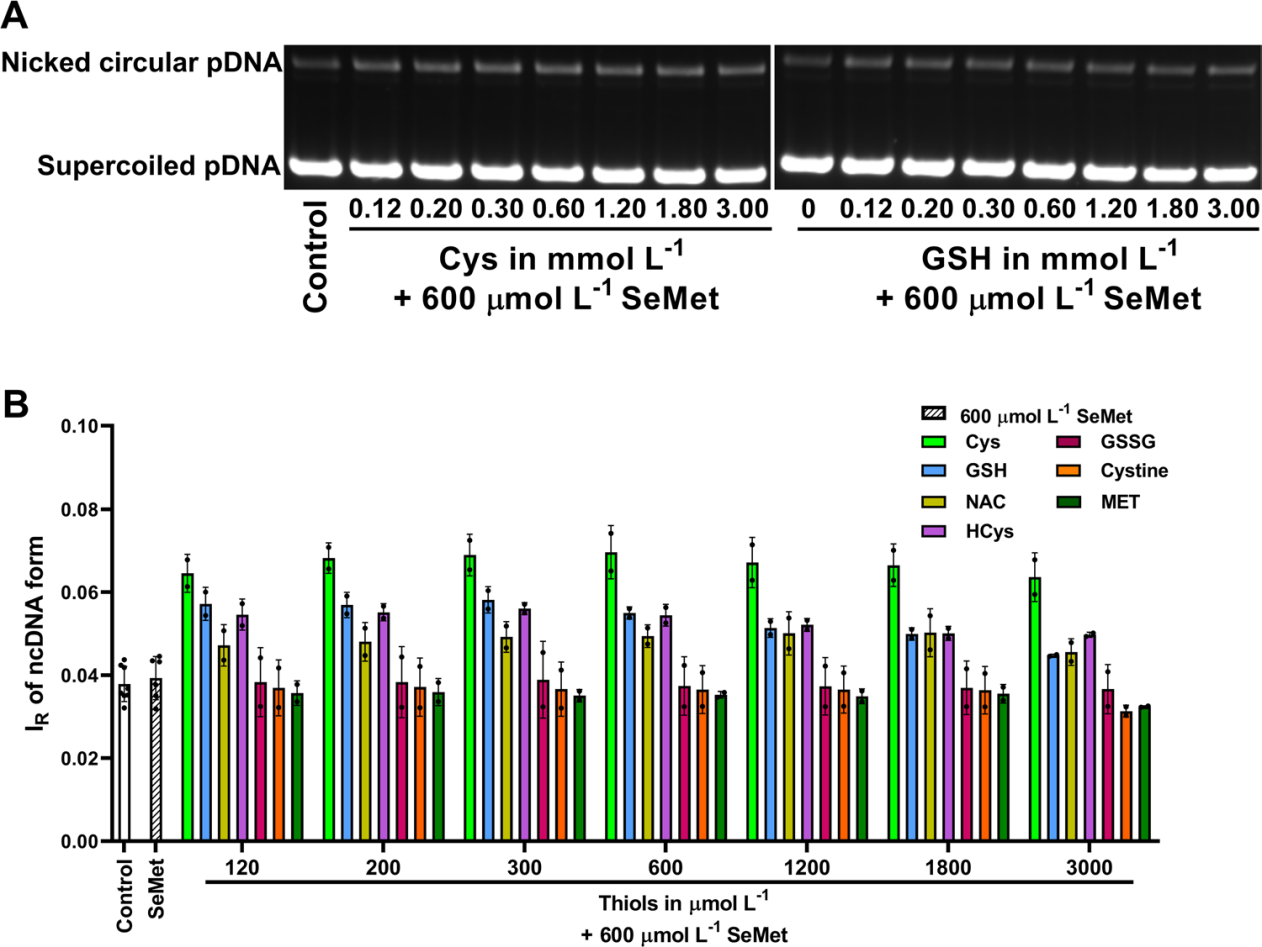
The pDNA (4 361 bp) cleavage by SeMet in the presence of thiols. The pDNA gels representing the effects of increasing concentrations of Cys or GSH (0–3 mmol L^−1^) on pDNA cleavage by 600 µmol L^−1^ SeMet (A). The bands at the bottom and top correspond to the circular supercoiled and nicked forms of pDNA, respectively. The final concentration of pDNA was 0.2 µg in 20 µL in a 25 mmol L^−1^ sodium phosphate buffer and 50 µmol L^−1^ DTPA at 37°C. The graphical representation of the pDNA cleavage by 600 µmol L^−1^ SeMet in the presence of increasing concentrations of thiols (B). Control is without any treatment. I_R_ of ncDNA form represents the relative intensity of the nicked circular pDNA. Data represent values from individual samples (*n* = 2 for all thiols) in a 25 mmol L^−1^ sodium phosphate buffer and 50 µmol L^−1^ DTPA at 37°C. Horizontal black marks indicate means ± SD

Since SeO_3_^2−^ can induce DNA cleavage in cultured cells [45–47] and can damage pDNA in the presence of H_2_S/HS^−^ [38], we wanted to find out whether the active species produced by the interaction of SeO_3_^2−^ with a panel of thiols can cause cleavage of pDNA. By using pDNA cleavage assay, we can monitor any activity leading to the disruption of sugar-phosphate DNA backbone. In control experiments, Cys, GSH, NAC, HCys, GSSG, cystine and MET alone at 0.64 and 5.12 mmol L^−1^ did not cleave pDNA (Fig. S21). However, HCys, GSH, Cys, NAC in the presence of 10 µmol L^−1^ SeO_3_^2−^ induced pDNA cleavage, whereas GSSG, cystine and MET had virtually no effect (Fig. 10). The cleavage was dependent on thiol concentrations in a bell-shaped manner. Moreover, the resulting profiles also showed different sensitivity to thiol concentrations, which means that the maximal cleavage effect was reached at different SeO_3_^2−^/thiol molar ratios. When considering maximal pDNA damage level of the SeO_3_^2−^/thiol mixture, the cleavage potency followed the order: HCys > GSH > Cys > NAC. Contrary to SeO_3_^2−^, the effect of thiols as Cys, GSH, NAC and HCys on pDNA cleavage in the presence of 600 µmol L^−1^ SeMet was minor, whereas GSSG, cystine and MET had no effect (Fig. 11). Interestingly, the most intense pro-cleavage potency was noticed for Cys at all concentrations.

### Effect of SOD on pDNA Cleavage Induced by Thiols/SeO_3_^2−^

**Fig. 12:**
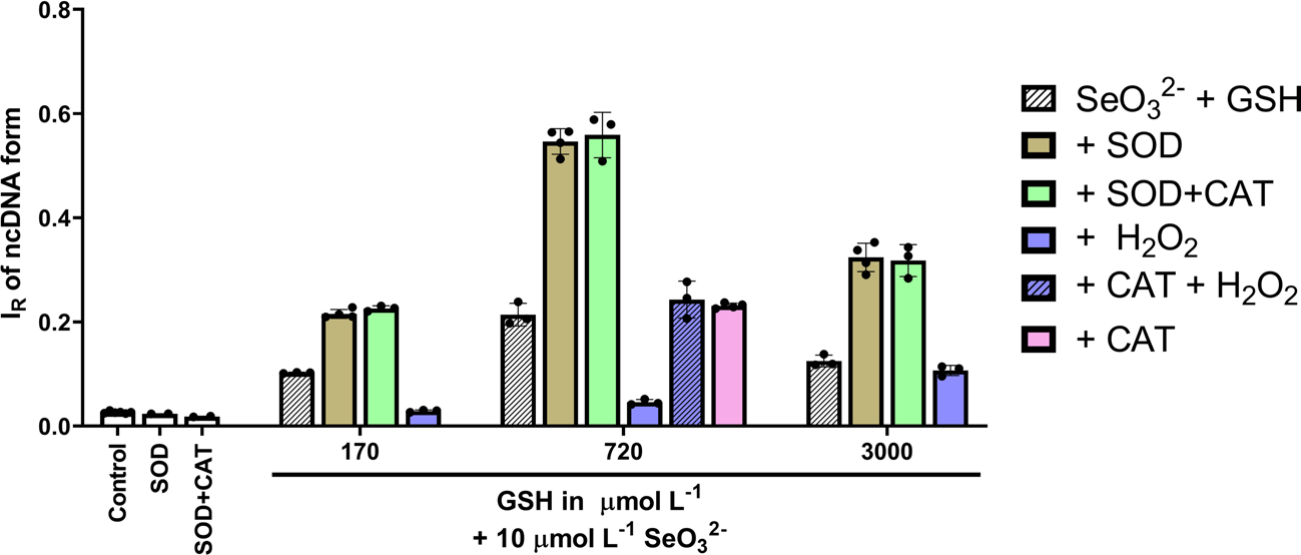
The participation of ROS in the pDNA cleavage potency of the GSH/SeO_3_^2−^ mixture. Effect of the SOD (200 U mL^–1^, 4 Units per sample) and SOD + CAT (200 + 200 U mL^–1^) was tested. pDNA cleavage by 10 µmol L^−1^ SeO_3_^2−^ in the presence of 170, 720 and 3000 µmol L^−1^ GSH and modulated by SOD (200 U mL^–1^), SOD + CAT (200 + 200 U mL^–1^), H_2_O_2_ (0.5 mmol L^–1^), CAT(200 U mL^–1^) + H_2_O_2_ (0.5 mmol L^–1^), CAT (200 U mL.^–1^). Control is without any treatment. I_R_ of ncDNA form represents the relative intensity of the nicked circular pDNA. Horizontal black marks indicate means ± SD, *n* ≥ 3

Since ^•^O_2_^–^ is produced during interaction of GSH with SeO_3_^2−^, we studied the participation of ^•^O_2_^–^ on pDNA cleavage in the presence of GSH/SeO_3_^2−^ (Fig. 12). In control experiments, SOD alone or SOD with CAT did not cleave pDNA. GSH/SeO_3_^2−^ in the molar ratios (170/10, 720/10 and 3000/10 in µmol L^–1^) cleaved pDNA in a bell shape manner similarly as showed in Fig. 10. SOD (200 U mL^–1^) potentiated the pDNA cleavage at all GSH/SeO_3_^2−^ molar ratios.

SOD accelerates the reaction of ^•^O_2_^–^ with itself to form H_2_O_2_ and O_2_ (2^•^O_2_^–^ + 2H^+^  → H_2_O_2_ + O_2_). To exclude a possibility that the increase of pDNA cleavage by SOD was not due to the reduction of H_2_O_2_ in the system and so producing hydroxyl radical (^•^OH), which can cleavage pDNA, CAT was used. CAT (200 U mL^–1^) did not influence pDNA cleavage that was accelerated by SOD. It also did not change the pDNA damage induced by GSH/SeO_3_^2−^ per se*.* This indicates that production of H_2_O_2_ by SOD was not responsible for the increase of pDNA cleavage. Notably, H_2_O_2_ (0.5 mmol L^−1^) inhibited GSH/SeO_3_^2−^-induced cleavage of pDNA, which was restored back by CAT (200 U mL^–1^).

### Time-Dependent Effect of GSH/SeO_3_^2−^ on Rat APW-Ps

**Fig. 13:**
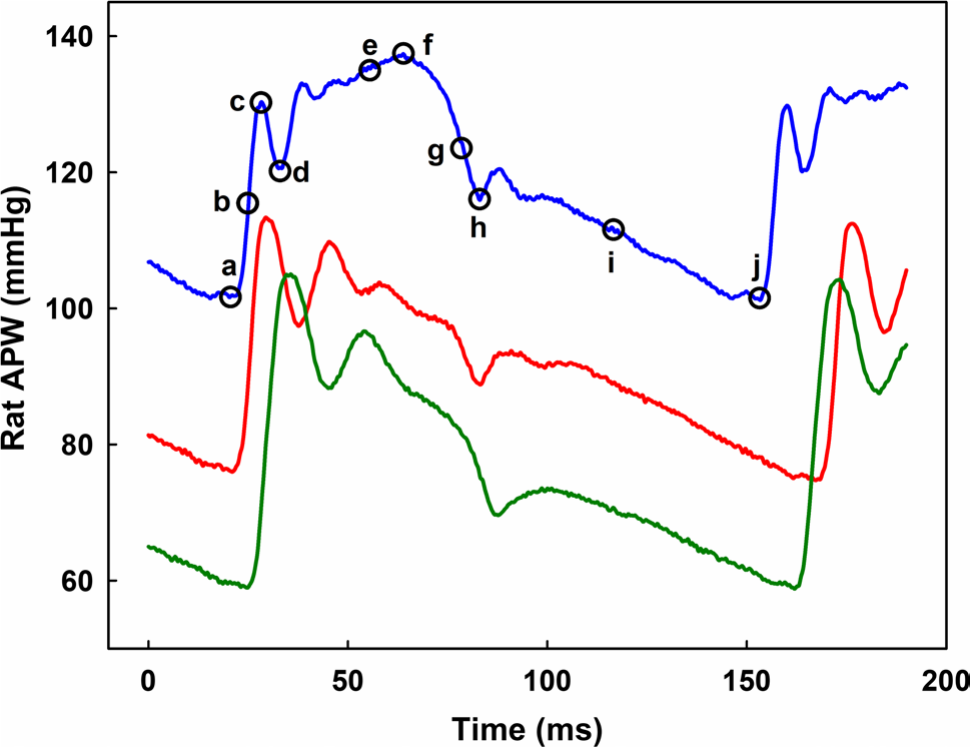
The left common carotid artery pulse waveform (APW) 30 s before (red), 30 s after (blue) and 2 min after (dark green) IV administration of the GSH/SeO_3_^2−^ mixture (75/12.5 in µmol kg^−1^). The mixture was incubated 30 s at 23 ± 1°C before administration. The APW (blue) with marked ten points a–j (black circles). The letters indicate: Diastolic BP (a, j); d*P*/d*t*_max_ (b); Systolic BP (c or f); First maximum BP 1Max (c); Anacrotic notch (d); (e) is BP at the middle of the time interval between dicrotic BP (h) and 1Max BP (c); Maximal-systolic BP (f); d*P*/d*t*_min_ (g); Dicrotic notch (h). (i) is BP at the middle of the time interval between (j) and dicrotic BP (h); d*P*/d*t*_d_ is in point i; Diastolic BP (j). The definition of the 35 APW-HPs calculated from these 10 points is described in Table S1

Since ROS play a crucial role in cardiovascular system [48–50] and products of the GSH/SeO_3_^2−^ mixture have reducing properties, effect of GSH/SeO_3_^2−^ mixture on rat blood pressure (BP) and other hemodynamic parameters was studied. Example of APWs, recorded at high resolution, in different conditions is shown in Fig. 13. Control APW (Fig. 13, red), showed detailed pulse waveform. The shape of the APW changed significantly after IV administration of the GSH/SeO_3_^2−^ mixture. At first, 30 s after the administration (blue), BP transiently increased, but after two min, it decreased (dark green). The APW (blue) is marked with ten points a–j (black circles), from which 35 APW-Ps were calculated, as defined in our previous studies [53, 54] and as it is described in Table S1.

**Fig. 14:**
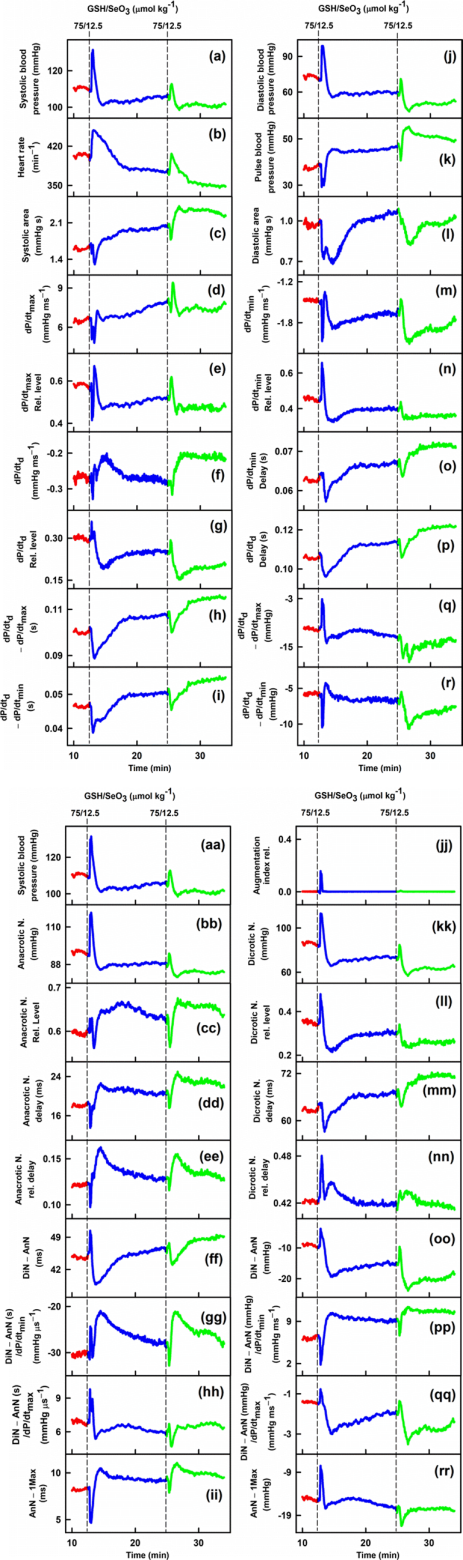
Time-dependent changes in rat 35 APW-Ps after two rounds of IV administration of the GSH/SeO_3_^2−^ mixture incubated 30 s at 23 ± 1°C. Theoretical initial concentration of GSH/SeO_3_^2−^ was calculated according to the rat weight as 75/12.5 µmol kg.^−1^. Vertical black dashed lines show the start of the mixture administration for 15 s. Definitions, units and abbreviations of APW-Ps evaluated from the APW are as explained previously [53, 54]

**Fig. 15:**
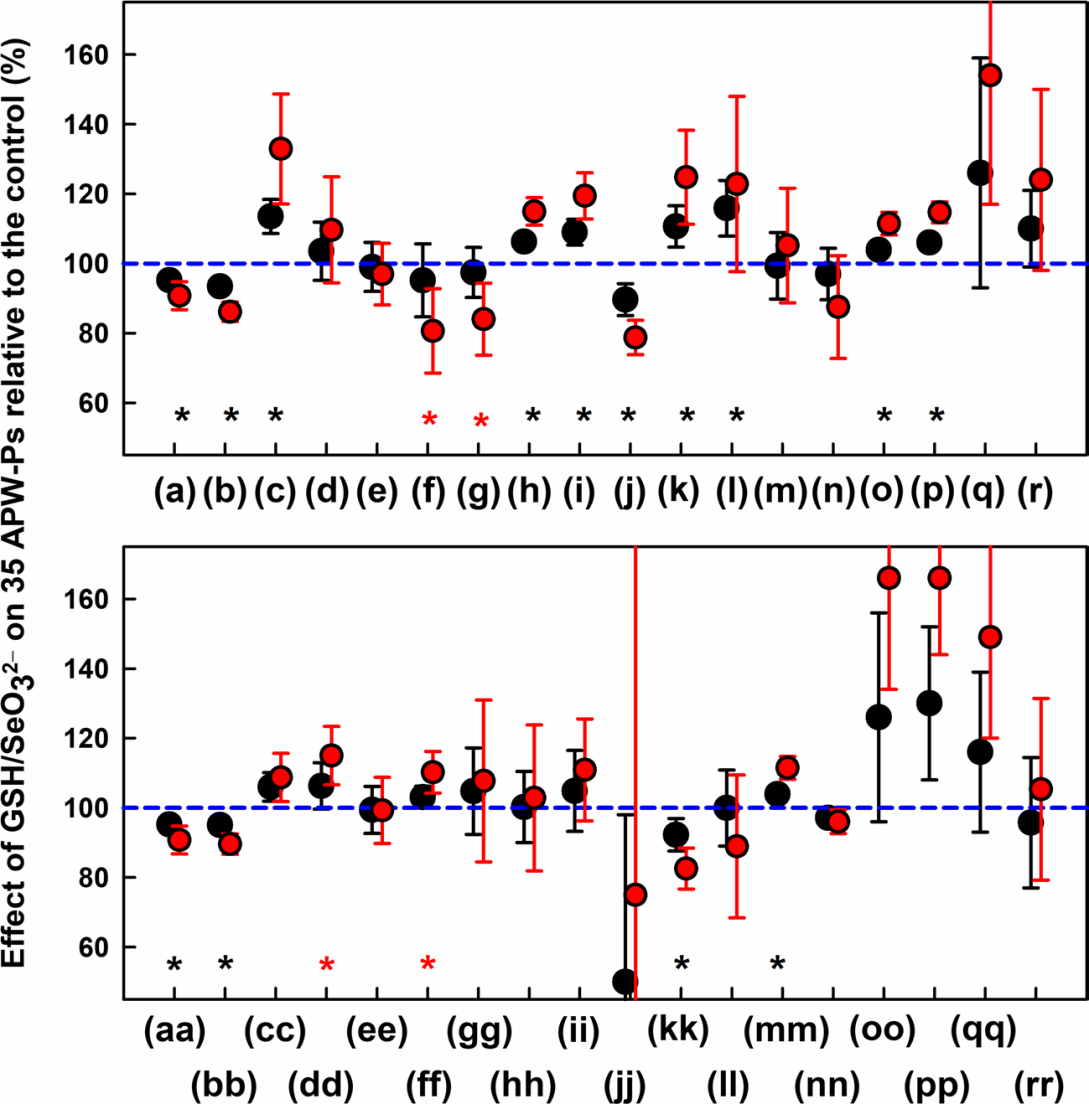
Relative changes in 35 APW-Ps in the 7th min after IV administration of the GSH/SeO_3_^2−^ mixture. Data was acquired in the 7th min after the first (black) or the second (red) IV administration of GSH/SeO_3_^2−^ mixture**,** respectively. The mixture was incubated for 30 s at 23 ± 1°C before IV administrated for 15 s. Theoretical initial concentration of GSH/SeO_3_^2−^ was calculated according to the rat weight as 75/12.5 µmol kg^−1^. The values are means ± SD, *n* = 8–10. In both administrations, the control values (100%) of APW-Ps were taken before the first administration. Mark* indicates significant difference vs. control (*P* < 0.001, paired *t*-test) after the first administration. Mark  indicates significant difference vs. control (*P* < 0.001, paired *t*-test) after the second administration

In control experiments, effects exerted by 12.5 µmol kg^−1^ SeO_3_^2−^ or 75 µmol kg^−1^ GSH alone on 35 APW-Ps were minor in comparison to the effects of their mixture (Fig. S22). However, IV administrations of the GSH/SeO_3_^2−^ mixture, incubated for 30 s, influenced all 35 rat APW-Ps. Systolic and diastolic BP as the well-known hemodynamic parameters responded to the administration in three phases. After the first administration, systolic and diastolic BP transiently increased for ≤ 1 min, then swiftly decreased for ~ 2 min and later they lasted mostly decreased during the remaining 10–12 min. This pattern was also reproducible after the GSH/SeO_3_^2−^ mixture (incubated for 30 s) was administrated again (Fig. 14). However, time-dependent effect was not uniform and varied for different APW-Ps indicating complex influence of the (inter) product(s) on APW. To better compare the effect of the GSH/SeO_3_^2−^ mixture, we took APW-Ps values according to systolic BP timing. All APW-Ps were evaluated at time of transient maximum and minimum of systolic BP, and also in the 7th min after mixture administration. The relative APW-Ps calculated as a percentage of the control are presented in Fig. S23. It is seen that the GSH/SeO_3_^2−^ mixture affected 35 APW-Ps in different way. Notably, after the second administration of the mixture, the long-term average effect on the most of the APW-Ps seems to be additive (Fig. 15). This means, if the APW-P was significantly changed after the first administration, this course also remained after the second administration. The mixture significantly decreased systolic and diastolic BP (a,j), heart rate (b), dP/dt_d_ (f), dP/dt_d_ relative level (g) and anacrotic and dicrotic notches (bb, kk) in additive manner. Contrary to this, it increased systolic and diastolic areas (c, l), parameters (h,i), pulse BP (k), dP/dt_min_ and dP/dt_d_ delay (o, p), anacrotic and dicrotic notch delay (dd, mm) and DiN – AnN (ff). Notably, most of the APW-Ps listed above were already significantly changed after the first administration. Similar effects of the GSH/SeO_3_^2−^ mixture incubated for 90 s were observed (Figs. S24, S25).

A comparison of the transient increase and decrease in systolic BP between the first and the second administration of the mixture is shown in Fig. S26. The second administration induced significantly lower increase of the systolic BP than the first one; however, it significantly deepened following decrease of systolic BP.

### Time-Dependent Vasoactive Effect of GSH/SeO_3_^2−^ on Isolated Arteries

**Fig. 16:**
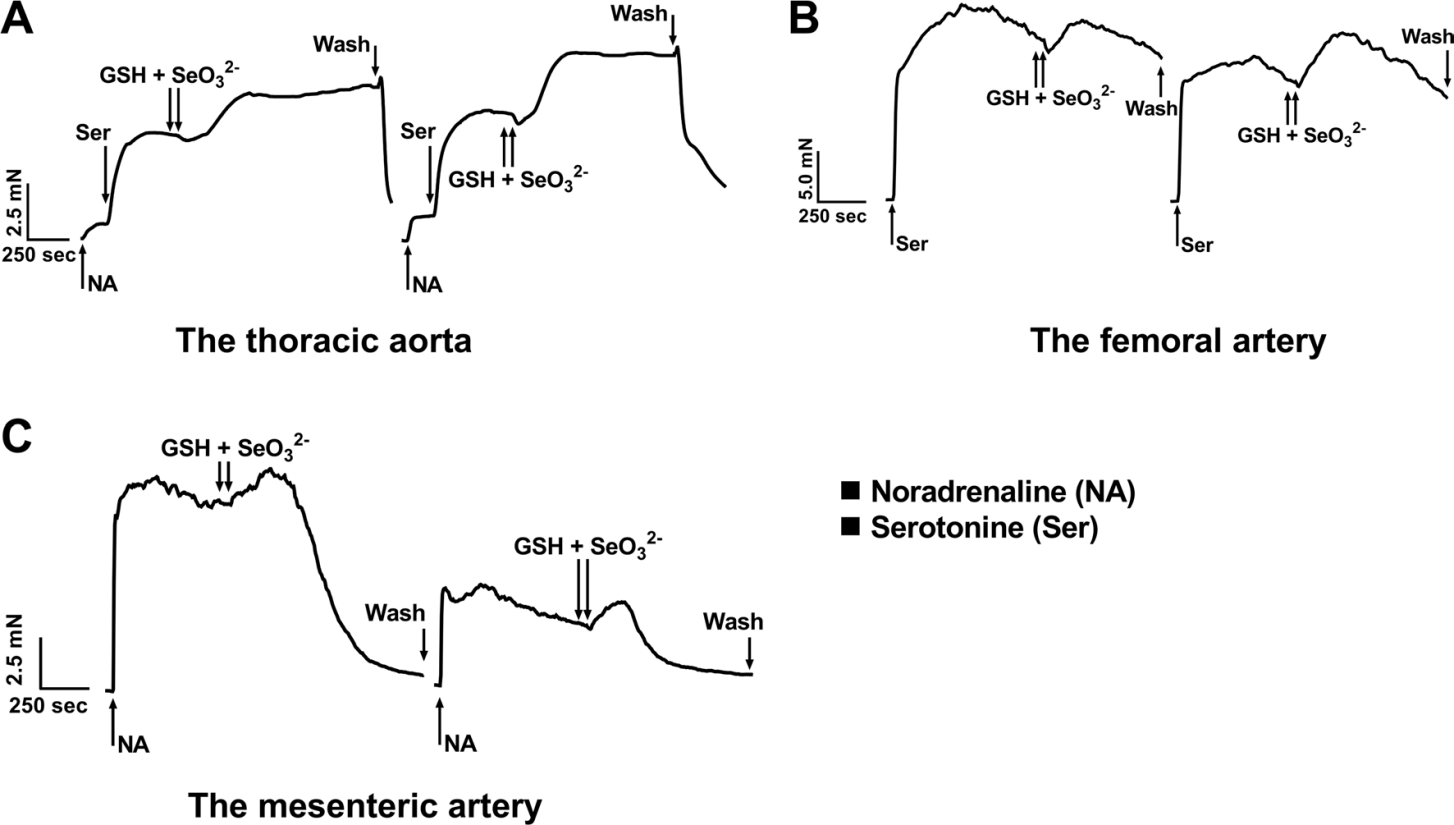
The records of time-dependent changes of tension of isolated thoracic aorta (A), the femoral (B) and the mesenteric arteries **(C)**. NA (1 µmol L^−1^) and Ser (10 µmol L^−1^) increased the tension of isolated thoracic aorta (A), Ser (1 µmol L^−1^) increased the tension of isolated femoral artery (B) and NA (10 µmol L^−1^) increased the tension of isolated mesenteric artery (C). The subsequent application of 500 µmol L^−1^ GSH and 40 µmol L^−1^ SeO_3_^2−^ evoked an additional change of the vascular tension. Then the working solution was washed out from the organ chamber and the addition of the compounds was repeated

To understand how arteries contributed to the changes of time-dependent systolic and diastolic BP (Figs. 14 and 15), the vasoactive effect of GSH/SeO_3_^2−^ on isolated thoracic aorta, femoral and mesenteric arteries was studied. The original traces of the vasoactive responses induced by the GSH/SeO_3_^2−^ mixture in three types of arteries are shown in Fig. 16A–C.

**Fig. 17:**
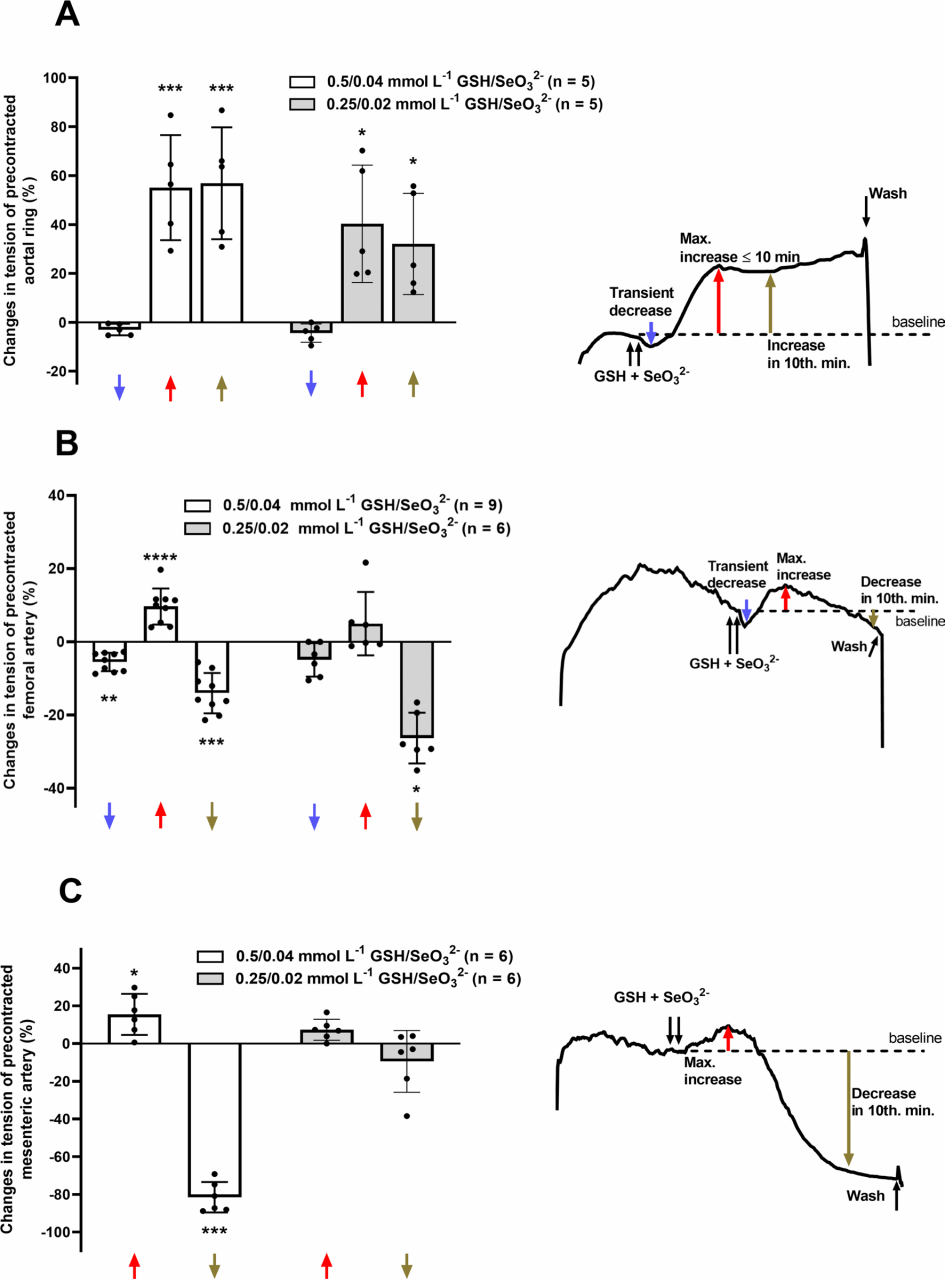
Relative effect of GSH/SeO_3_^2−^ on precontracted thoracic aorta (A), the femoral (B) and the mesenteric (C) arteries. The arteries were precontracted by (A) NA (1 µmol L^−1^) and Ser (10 µmol L^−1^), (B) Ser (1 µmol L^−1^) and (C) NA (10 µmol L^−1^). White bars indicate GSH 500 µmol L^−1^ + SeO_3_^2−^ 40 µmol L^−1^ and grey bars GSH 250 µmol L^−1^ + SeO_3_^2−^ 20 µmol L^−1^. The blue arrow represents transient decrease and the red arrow maximal increase in artery tension. The dark yellow arrow represents change in tension in the 10th min. Specifically for (A), the increase of tension in the 10th min can be equal to the maximal increase variable if the tension is monotonic. The changes in tension are expressed relatively (%) to the tension of precontracted artery measured just before application, which was considered as a control. Values are means ± SD. Statistics: one-way ANOVA with RM followed by Dunnett’s multiple comparisons test: *P < 0.05 vs. control, **P < 0.01 vs. control, ***P < 0.001 vs. control, ****P < 0.001 vs. control

In thoracic aorta, noradrenaline (NA, 1 µmol L^−1^) and a subsequent application of serotonin (Ser, 10 µmol L^−1^) increased the tension of the arterial wall. Application of GSH/SeO_3_^2−^ had a mild relaxant response followed by a significant long contraction for more than 10 min. However, in some cases this contraction spontaneously and slowly weakened. This vasoactive effect was observed at two concentrations (GSH 250 µmol L^−1^ + SeO_3_^2−^ 20 µmol L^−1^ and GSH 500 µmol L^−1^ + SeO_3_^2−^ 40 µmol L^−1^) (Fig. 17A). This character of the responses induced by GSH/SeO_3_^2−^ was reproducible, since after the reperfusion of the medium from the organ bath, its repeated application evoked a similar vasoactive effect (Fig. 16A). Contraction of the thoracic aorta measured in the 10th min was observed after every administration of GSH/SeO_3_^2−^.

In the femoral artery, serotonin (Ser, 10 µmol L^−1^) increased arterial tension. Application of GSH/ SeO_3_^2−^ led to the negligible relaxation followed by transiently increased tension with subsequent decrease. It is suggested that the spontaneous decrease of the tension might be due to specific property of the femoral artery (Figs. 16B, 17B). A similar nature of the GSH/SeO_3_^2−^-induced responses was observed after washing out the solution and reapplying GSH/SeO_3_^2−^ (Fig. 16B).

In mesenteric artery, noradrenaline (NA, 10 µmol L^−1^) increased the tension of the artery. Application of GSH/SeO_3_^2−^ transiently increased the tension with subsequent significant decrease, which was prolonged for more than 10 min (Figs. 16C and 17C). Notably, after application of 500 µmol L^−1^ GSH + 40 µmol L^−1^ SeO_3_^2−^ (*n* = 6), the artery nearly completely relaxed and after wash-out of the solution, it was not possible to increase arterial tension by NA again (*n* = 3) or the tension was increased only partially (Figs. 16C and 17C, n = 3). This was not the case of 250 µmol L^−1^ GSH + 20 µmol L^−1^ SeO_3_^2−^, when the application of NA after reperfusion produced the tension as strong as before.

## Discussion

### Reducing Properties of the Thiol/SeO_3_^2−^ Mixtures

In this study, we provide evidence that the products of the thiol/SeO_3_^2−^ interaction have numerous effects in vitro, ex vivo and in vivo*,* which may be relevant to biological and physiological role of SeO_3_^2–^. In in vitro study, we have used model of reduction of the ^•^cPTIO stable radical to compare the properties of different SeO_3_^2−^/thiol mixtures. We confirmed that SeO_3_^2−^ is not an antioxidant by itself, but it has significant reducing properties in the presence of Cys, GSH or HCys, but not in the presence of cystine, GSSG or MET. The reducing properties strongly depended on thiol/SeO_3_^2−^ molar ratio. Additionally, similar reduction properties of SeO_3_^2−^ were also observed upon its interaction with H_2_S/HS^−^ [38].

The reducing properties of the thiol/SeO_3_^2−^ active species were more significant at high thiol/SeO_3_^2−^ molar ratios and they decreased significantly with decreasing molar ratios. This confirms the formation of different active species with diverse biological activities at different times and molar ratios [2, 5, 15, 18, 61]. The intracellular concentration of GSH (0.5–10 mmol L^−1^), Cys (∼200 μmol L^−1^) or HCys (∼10 μmol L^−1^) [57] is high enough to interact with 0.3–10 μmol L^−1^ SeO_3_^2−^ and to produce biologically active species. Therefore, it is suggested that the thiol/SeO_3_^2−^ interaction and the derived production of reducing species from this interaction is physiologically relevant, and it may contribute to the explanation of numerous antioxidant effects of SeO_3_^2−^ both ex vivo and in vivo that have been reported in previous studies [1, 6, 10, 12, 26].

It has been demonstrated that SeO_3_^2−^ in the presence of GSH was a reductive catalyst for oxidized cytochrome *c* and methemoglobin [62–64]. Besides, selenopersulfide anion (RSSe^−^), an intermediate of SeO_3_^2−^ reduction by thiols, has been suggested as the potential underlying catalytically active species. However, the effects of extensive and different molar thiols/SeO_3_^2−^ ratios have not been yet studied, to the best of our knowledge. Herein, in the presence of an excess of thiols, similar reduction rates and extent of the ^•^cPTIO reduction was observed after adding of 100 µmol L^−1 •^cPTIO 6-times in a row into GSH/SeO_3_^2−^, Cys/SeO_3_^2−^, HCys/SeO_3_^2−^ or D-Cys/SeO_3_^2−^, indicating that thiols/SeO_3_^2−^ have noteworthy catalytic reducing properties. Similar results were observed in the sodium phosphate and Tris–HCl buffers.

SeO_3_^2–^ is known to react spontaneously with GSH to initially produce GS-Se-SG, and in the presence of an excess of GSH, GSSeSG is further reduced to GSSeH. GSSeH either spontaneously dismutates into Se^(0)^ and GSH, or is further reduced by GSH to yield H_2_Se (Eqs. (1)–(14)). And H_2_Se is readily oxidized by O_2_ into Se^(0)^ [17, 58, 61]. Therefore, one of the leading species after reaction of SeO_3_^2−^ with thiols is H_2_Se (HSe^–^/Se^2–^), which reacts with Ag forming Ag_2_Se precipitate [58, 59]. Since we found that Ag at molar ratios Ag/SeO_3_^2−^  ≥ 2 totally blocked the ^•^cPTIO reduction (Fig. 4), but not at lower < 2 ratios, and that Na_2_Se reduced ^•^cPTIO (Fig. 4C,D), it is suggested that H_2_Se is mainly involved in the reduction of ^•^cPTIO. However, other negatively charged species, as for example, RSSe^−^, cannot be excluded, as they could also have the ability to precipitate with Ag.

Reactions of SeO_3_^2−^ with GSH have been studied and relevant ones to our results are summarized from [19, 20, 58]:

1 $${{{\text{SeO}}}_{3}}^{2-}+4{\text{GSH}}+2{{\text{H}}}^{+}\to \mathrm{GSSeSG }+\mathrm{ GSSG }+ 3{{\text{H}}}_{2}{\text{O}}$$

2 $$\mathrm{GSSeSG }+\mathrm{ GSH }\to \mathrm{ GSSeH }+\mathrm{ GSSG}$$

3 $$\mathrm{GSSeH }\to {{\text{Se}}}^{(0)}+{\text{GSH}}$$

4 $$\mathrm{GSSeH }+\mathrm{ GSH }\to {{\text{H}}}_{2}\mathrm{Se }+\mathrm{ GSSG}$$

5 $${{\text{H}}}_{2}\mathrm{Se }+ 1/2{{\text{O}}}_{2}\to {{\text{Se}}}^{\left(0\right)}+{{\text{H}}}_{2}{\text{O}}$$

6 $${{\text{H}}}_{2}{\text{Se}}+1/2{{\text{O}}}_{2} + 2\mathrm{GSH }\to {{\text{H}}}_{2}\mathrm{Se }+\mathrm{ GSSG }+ {{\text{H}}}_{2}{\text{O}}$$

7 $$\mathrm{RSSR }+ 2{{\text{RSe}}}^{-} + 2{{\text{H}}}^{+} \leftrightarrow \mathrm{ RSeSeR }+ 2{\text{RSH}}$$

8 $${{\text{Se}}}^{(0)} + 2\mathrm{RSH }\to {{\text{H}}}_{2}\mathrm{Se }+\mathrm{ RSSR}$$

9 $${{{\text{SeO}}}_{3}}^{2-}+4\mathrm{GSH }+ 2{{\text{H}}}^{+} \to {{\text{Se}}}^{(0)}+2\mathrm{GSSG }+ 3{{\text{H}}}_{2}{\text{O}}$$

10 $$\mathrm{GSH }+\mathrm{ GSSeSG }\leftrightarrow \mathrm{ GSSG }+\mathrm{ GSSeH}$$

11 $$\mathrm{GSSeH }\leftrightarrow \mathrm{ GSH }+ {{\text{Se}}}^{(0)}$$

GSSe^–^ has been proposed to be the active electron transfer catalyst in the case of cytochrome c reduction by [63]:

12 $${{\text{GSSe}}}^{-}+{{\text{Fe}}}^{{\text{III}}}\mathrm{Cytc }{\leftrightarrow }^{\bullet }\mathrm{GSSe }+ {{\text{Fe}}}^{{\text{II}}}{\text{Cytc}}$$

13 $$\mathrm{GSH }+ 2 {{\text{GSSe}}}^{\bullet } \to {{\text{GSSe}}}^{-} + {{\text{H}}}^{+} +\mathrm{ GSSeSG}$$

Additionally, ^•^O_2_^−^ is produced when SeO_3_^2−^ reacts with GSH [14, 17, 65, 66] and the following stoichiometry has been proposed [17]:

14 $${3{{\text{SeO}}}_{3}}^{2-}+6\mathrm{GSH }+ 4{{\text{H}}}^{+} \to 3\mathrm{GSSeSG }+ {2}^{\bullet }{{{\text{O}}}_{2}}^{-}+ 5{{\text{H}}}_{2}{\text{O}}$$

There may be several possibilities that would explain the reduction of the ^•^cPTIO radical by the GSH/SeO_3_^2−^ mixture. By analogy with the reactions at a high excess of GSH (or Cys, or HCys), as proposed by Prütz [19] and Cupp-Sutton [58], we may suggest a direct interaction SeO_3_^2−^  + GSH + ^•^cPTIO that would lead to ^•^cPTIO reduction and GSSeSG production.

In our previous work, we observed that Na_2_Se stock solution added to ^•^cPTIO solution did not reduce it, but the reduction happened when ^•^cPTIO solution contained GSH [67]. As consequence, we found that H_2_Se/HSe^–^ is significantly oxidized in our buffer solution, therefore we rather added ^•^cPTIO solution directly to a small amount of Na_2_Se powder and measured UV–Vis spectra immediately. Using this approach, ^•^cPTIO was reduced by Na_2_Se solution in about 4 s, thus suggesting that H_2_Se/HSe^–^ is involved in the catalytic ^•^cPTIO reduction that takes place in the presence of SeO_3_^2−^ and an excess of thiols. As a second main possibility that would explain the observed ^•^cPTIO reduction by GSH/SeO_3_^2−^ in the presence of GSH, the following equations are suggested:

1 $${{{\text{SeO}}}_{3}}^{2-}+4\mathrm{GSH }+ 2{{\text{H}}}^{+} \to \mathrm{ GSSeSG }+\mathrm{ GSSG }+ 3{{\text{H}}}_{2}{\text{O}}$$

2 $$\mathrm{GSSeSG }+\mathrm{ GSH }\to \mathrm{ GSSeH }+\mathrm{ GSSG}$$

4 $$\mathrm{GSSeH }+\mathrm{ GSH }\to {{\text{H}}}_{2}\mathrm{Se }+\mathrm{ GSSG}$$

15 $${{\text{H}}}_{2}\mathrm{Se }+ {2}^{\bullet }\mathrm{cPTIO }\to {{\text{Se}}}^{(0)}+ 2{\text{cPTIO}}-{\text{H}}$$

16 $${{\text{Se}}}^{(0)}+2\mathrm{ GSH }\to {{\text{H}}}_{2}\mathrm{Se }+\mathrm{ GSSG}$$

15 $${{\text{H}}}_{2}\mathrm{Se }+ {2}^{\bullet }\mathrm{cPTIO }\to {{\text{Se}}}^{(0)}+2{\text{cPTIO}}-{\text{H}}$$

17 $${{\text{Se}}}^{(0)}+\mathrm{GSH }\to \mathrm{ GSSeH}$$

4 $$\mathrm{GSSeH }+\mathrm{ GSH }\to {{\text{H}}}_{2}\mathrm{Se }+\mathrm{ GSSG}$$

15 $${{\text{H}}}_{2}\mathrm{Se }+ {2}^{\bullet }\mathrm{cPTIO }\to {{\text{Se}}}^{(0)}+ 2{\text{cPTIO}}-{\text{H}}$$

The third possibility is similar to the proposed catalytic reduction of oxidized cytochrome c by GSSe^–^ (Eqs. (12) and (13)) [63]:

1 $${{{\text{SeO}}}_{3}}^{2-}+4\mathrm{GSH }+ 2{{\text{H}}}^{+} \to \mathrm{ GSSeSG }+\mathrm{ GSSG }+ 3{{\text{H}}}_{2}{\text{O}}$$

2 $$\mathrm{GSSeSG }+\mathrm{ GSH }\to \mathrm{ GSSeH }+\mathrm{ GSSG}$$

18 $${{\text{GSSe}}}^{-} + {{\text{H}}}^{+}{+}^{\bullet }{\text{cPTIO}}{\to }^{\bullet }\mathrm{GSSe }+\mathrm{ cPTIO}-{\text{H}}$$

19 $${\text{GSH}}+2 {{\text{GSSe}}}^{\bullet }\to {{\text{GSSe}}}^{-}+ {{\text{H}}}^{+}+{\text{GSSeSG}}$$

In addition, a partial reduction of ^**•**^cPTIO might be suggested as separated reported reactions that could happen consecutively: the generation of superoxide radical by SeO_3_^2−^/GSH mixture and the subsequent quenching of the superoxide by ^**•**^cPTIO radical, as follows [14, 68].

1 $${{{\text{SeO}}}_{3}}^{2-}\hspace{0.17em}+\hspace{0.17em}4{\text{GSH}}\hspace{0.17em}+\hspace{0.17em}2{{\text{H}}}^{+} \hspace{0.17em}\to \hspace{0.17em}{\text{GSSeSG}}\hspace{0.17em}+\hspace{0.17em}{\text{GSSG}}\hspace{0.17em}+\hspace{0.17em}3{{\text{H}}}_{2}{\text{O}}$$

2 $${\text{GSSeSG}}\hspace{0.17em}+\hspace{0.17em}{\text{GSH}}\hspace{0.17em}\to \hspace{0.17em}{\text{GSSeH}}\hspace{0.17em}+\hspace{0.17em}{\text{GSSG}}$$

4 $${\text{GSSeH}}\hspace{0.17em}+\hspace{0.17em}{\text{GSH}}\hspace{0.17em}\to \hspace{0.17em}{{\text{H}}}_{2}{\text{Se}}\hspace{0.17em}+\hspace{0.17em}{\text{GSSG}}$$

20 $${{\text{H}}}_{2}{\text{Se}}\hspace{0.17em}+\hspace{0.17em}2{{\text{O}}}_{2}\hspace{0.17em}\to \hspace{0.17em}{{\text{Se}}}^{(0)}\hspace{0.17em}+\hspace{0.17em}{2{{\text{O}}}_{2}}^{\bullet -}\hspace{0.17em}+\hspace{0.17em}2{{\text{H}}}^{+}$$

21 $${2}^{\bullet }{\text{cPTIO}}\hspace{0.17em}+\hspace{0.17em}{2{{\text{O}}}_{2}}^{\bullet -}\hspace{0.17em}+\hspace{0.17em}2{{\text{H}}}^{+} \hspace{0.17em}\to \hspace{0.17em}2{\text{cPTIO}}-{\text{H}}\hspace{0.17em}+\hspace{0.17em}2{{\text{O}}}_{2}$$

Additional research needs to be performed, including kinetic measurements, or the utilization of different techniques such as cyclic voltammetry, to ascertain which and to which extent of the proposed mechanisms is responsible for the experimental ^•^cPTIO radical reduction observed in this work.

The ^•^cPTIO reduction induced by 200–300 nmol L^−1^ SeO_3_^2−^ with 2 mmol L^−1^ Cys or GSH was higher than that of 100 µmol L^−1^ Trolox (without or with SeO_3_^2−^ or GSH). This observed experimental fact indicates that the effect of the thiol/SeO_3_^2−^ mixture to reduce ^•^cPTIO was 300-times more potent than the potency of Trolox. In contrast to the thiol/SeO_3_^2−^ mixtures, the activity of Trolox was not influenced by the presence of SeO_3_^2−^, confirming a specific interaction of SeO_3_^2−^ with thiols occurs.

Since colloidal Se^(0)^ and/or unspecified Se-nanoparticles were not formed in the presence of SOD in the thiol/SeO_3_^2–^ mixture, it is confirmed that ^•^O_2_^−^ is involved in the formation of these colloidal Se^(0)^ species, but the elimination of ^•^O_2_^−^ did not influence reduction of ^•^cPTIO. Therefore, it is suggested that the reactions could be described by Eqs. (1), (2), (4) and (14). The obtained results provide evidence that ^•^O_2_^−^ is important for further GSH/SeO_3_^2–^ reactions resulting to colloidal Se^(0)^ and/or unknown Se-containing nanoparticles, but not for the reduction of ^•^cPTIO.

We do not know the basics of the interactions resulting in the increase rate of the ^•^cPTIO reduction when the GSH/SeO_3_^2−^ molar ratio of 6,666/1 increased to 16,666/1 and further to 33,333/1, or when the Cys/SeO_3_^2−^ molar ratio of 10,000/1 increased to 20,000/1. Since excess of GSH can kinetically stabilize H_2_Se from oxidation (Eq. (6), [58]), it might be possible that the higher rate of the ^•^cPTIO reduction resulted from the increased stability of H_2_Se. We may speculate that, as H_2_Se is generally present in physiological media (or buffers) in HSe^–^ form, the anionic form can induce a dipole in the sulfur atom of the GSH (or of the remaining thiols tested), rendering an intermolecular van-der-Waals interaction that may stabilize the HSe^–^. This would explain the observed increased reduction rate when the molar ratio of the GSH (or thiol) in respect to selenite augments.

From the comparison of the thiol-based compounds/SeO_3_^2−^ potencies to reduce ^•^cPTIO, it is evident that the thiol group is necessary to form effective reducing species. From the comparison of SeO_3_^2−^ with SeMet to form complexes with thiols, the same order of potency was observed (Cys > HCys > GSH > NAC > cystine = GSSG = Met = no effect), but the effect of SeMet was significantly lower than the effect of SeO_3_^2−^, which may imply that the active products of the interaction are less potent. Our data suggest that SeMet interacting with high concentration of GSH can have biologically relevant effects. Data showing that SeO_3_^2−^ is more active than SeMet in terms of thiol-promoted reducing species, which may contribute to the explanation of their different toxicity or biological activities [37, 69, 70]. Interestingly, the thiols that present a hydrogen atom bound to the sulfur atom (Cys, HCys, GSH, NAC) are more potent reductors than the non-protonated ones (cystine, GSSG, Met); and among the protonated thiols, the potency is approximately inversely correlated to the size. This is aligned with the speculated potential formation of van-der-Waals interactions with HSe^–^: smaller compounds with a protonated thiol would be more accessible to these interactions that bigger compounds and that sulfides or disulfides.

It is known that free radicals contribute to the development of many lifestyle diseases, including diabetes, atherosclerosis, as well as multiple sclerosis, Alzheimer’s disease, Parkinson’s disease or cancer. In addition, they damage the collagen that builds the skin, bones, muscles, tendons and tissues of internal organs. As a result, oxidative stress accelerates also the aging process [3, 11, 12, 35–37, 71, 72]. Therefore, our findings that selenite/thiol interaction products have significant reducing properties can lead to further application studies that may contribute to their use in medical practice.

### *In vitro *Cleavage of pDNA Induced by Thiol/SeO_3_^2−^ Mixtures and Participation of ROS

It was reported that SeO_3_^2−^ induces DNA single- and/or double-strands breaks in isolated cells [45–47]. Interestingly, in our study neither thiols nor SeO_3_^2−^ cleaved pDNA. This means that SeO_3_^2−^-induced pDNA breaks need some prerequisite available under in vivo conditions in a cell. In contrast, a mixture of SeO_3_^2−^ and thiols became highly capable to damage DNA: it increased the pDNA cleavage with a bell-shaped concentration dependence, indicating that thiol-containing compounds might be responsible and prerequisite factors for SeO_3_^2−−^mediated DNA strand breakage in vivo. These concentration dependencies are different from those found in the case of the ^•^cPTIO reduction, in which the reduction potency increased with the increasing thiol/SeO_3_^2−^ ratio, and the biphasic effect of SeO_3_^2−^ concentration was observed at constant Cys or GSH. The order of potency to cleavage pDNA in the presence of 10 µmol L^−1^ SeO_3_^2−^, HCys > GSH > Cys > NAC > cystine = GSSG = Met = no effect, was different than the order of potency to reduce ^•^cPTIO (Cys > HCys > GSH > NAC > cystine = GSSG = Met = no effect). Additionally, the maximal effects produced by the diverse thiol/SeO_3_^2−^ ratios evaluated were different in the ^•^cPTIO reduction and pDNA cleavage studies. These results suggest the possibility that different mechanisms are involved in these two effects evaluated. In agreement with our study, cleavage of plasmid DNA by SeO_3_^2–^ in the presence of GSH was reported [73].

Similarly, as found for reducing activities, the thiols/SeO_3_^2−^ mixture was more active than the thiols/SeMet mixture in terms of pDNA cleavage. This fact may explain our previous in vivo findings that demonstrated that SeMet is far less effective than SeO_3_^2−^ in mediating the toxic and mutagenic effects and DNA double-strand breakage, when both Se compounds were applied at the equimolar concentrations [74]. Based on the data presented herein, we may additionally propose that SeMet was less effective than SeO_3_^2−^ in producing thiol-assisted species that are responsible for the mentioned biological effects in vivo.

SeO_3_^2−^ oxidizes the majority of the tested thiols in vitro*,* thereby generating ^•^O_2_^–^ and other ROS, which might be associated with the observed SeO_3_^2−^-mediated toxicity and free radical stress in vivo [5, 13, 15, 16, 21]. Depending on the physiological/experimental conditions, Se can act as an antioxidant or a prooxidant [75]. The effect of SOD to potentiate the thiols/SeO_3_^2−^-induced cleavage of pDNA indicates that the ^•^O_2_^–^ produced from GSH/SeO_3_^2−^ reactions partially prevented pDNA cleavage. It is suggested that ^•^O_2_^–^ can interact with unknown radical(s) produced by the GSH/SeO_3_^2–^ interaction, radicals that have the ability to cleave pDNA. Interestingly, H_2_O_2_ inhibited the pDNA cleavage, which might be explained by GSH oxidation. This effect was partially overridden at higher GSH concentration and thus due to the higher availability of GSH in interaction with SeO_3_^2−^. ROS are mostly connected with negative effect in living organisms [36, 50, 76]. Notably, in our experimental assay, ^•^O_2_^–^ had positive effect on pDNA cleavage, but the specific chemical interactions underlying this effect are not known.

The direct in vitro effect of sodium selenide (Na_2_Se) on plasmid DNA was also studied [73]. It was found that hydrogen selenide (H_2_Se/HSe^–^/Se^2–^) induced DNA phosphodiester-bond breaks in the presence of O_2_. Hydroxyl radicals produced during the reaction of hydrogen selenide with dissolved O_2_, but not superoxide anions or hydrogen peroxide, were suggested to be responsible for the effect. Based on this, we can propose that H_2_Se produced during thiols/Se_3_^2–^ interaction (Eq. (4)) plays an important role in pDNA cleavage and products of H_2_Se oxidation, possibly hydroxyl radicals, are responsible for pDNA damage observed in our study. However, it cannot be excluded the involvement of other reactive species generated during thiols/Se_3_^2–^ interaction.

The concentrations of GSH, Cys and HCys vary in different organism compartments and can change under pathological conditions [77, 78]. Based on our results, we suggest that the reducing effect and pDNA damaging properties of thiols/SeO_3_^2−^ depend on the concentrations of GSH, Cys and HCys in situ. The products of interactions of SeO_3_^2−^ with GSH, Cys and HCys had pronounced reducing and pDNA damaging properties. The state of the two central thiol/disulfide redox couples in human plasma, Cys/cystine and GSH/GSSG is implicated in many biological processes and diseases, including enzyme catalysis, gene expression, and pathway signaling [79, 80]. It is assumed that the properties of the thiol/ SeO_3_^2−^ mixture detected in vitro can be applied in a living organism, depending not only on the concentration of thiols in situ, but also on the changes in the thiol/disulfide ratio and thus can influence the biological effects of SeO_3_^2−^.

### *In vivo* Effects of the GSH/ SeO_3_^2−^ Mixture on Hemodynamic Parameters

It was hypothesized that it might be possible to characterize the cardiovascular system through the analysis of the detailed shape of APW [81–84]. In this study, we used 35 APW-Ps derived from APW to characterize changes in cardiovascular system after IV application of the GSH/SeO_3_^2−^ mixture. These changes might create specific patterns related to cardiovascular system as we described in our previous studies [54, 85, 86].

The effects of the GSH/SeO_3_^2−^ mixture on systolic/diastolic BP can be divided into the three phases: transient BP increase, then swift BP decrease and the long-term BP reduction. Since the systolic BP transient increase was lower and the transient decrease was deeper after the second administration of the GSH/SeO_3_^2−^ versus the first one, it is evident that the transient decrease BP is not a subsequent physiological response of the cardiovascular system to the transient increase BP. Therefore, it is suggested that unknown active species produced at the beginning of the GSH/SeO_3_^2−^ interaction and/or produced subsequently after interaction with blood components transiently increase BP and affect other APW-Ps. This was followed by the hemodynamic response to another active species produced which decreased transiently BP and changed other APW-Ps. These active products might have a short circulating time due to their quick interaction with the biological components of the cardiovascular system and/or rapid removal in kidneys. However, it is suggested that the long-term lowering effect on systolic and diastolic BP and the changes of several other APW-Ps resulted from the relative strong binding of unknown active species to receptor(s)/protein(s) regulating cardiovascular system. In our previous work, we found that phthalic selenoanhydride (1–2 µmol L^–1^) prepared in physiological buffer releases various reactive Se species including H_2_Se and that it transiently decreases rat BP [18, 53]. This fact supports the hypothesis that Se derivatives that reduce BP in animal models can be potentially therapeutic agents against hypertension. Therefore, the recognition of the active species and mechanisms of action in cardiovascular system is a challenge for future research works with selenocompounds.

### *Ex vivo *Vasoactive Effect of GSH/SeO_3_^2−^ on Isolated Arteries

In our previous work, we found that various reactive Se species, including H_2_Se, released from phthalic selenoanhydride were able to relax precontracted mesenteric and femoral arteries, whereas it showed a moderate vasorelaxation effect on thoracic aorta [18, 53]. Notably, similar results were found for the GSH/SeO_3_^2−^ mixture, with the exception of the thoracic aorta.

In our ex vivo study, the vasoactive effect of GSH/SeO_3_^2−^ on isolated precontracted arteries was investigated to know which part of the arterial tree contributed to the observed changes of time-dependent systolic and diastolic BP and other APW-Ps. Interestingly, each part of the tree responded significantly differently to GSH/SeO_3_^2−^. Like in the case of the effects of GSH/SeO_3_^2−^ on APW-Ps, the analysis of the observed time-dependent vasoactive effects on arteries confirms that the various active species might be produced at different time from the reaction of SeO_3_^2−^ with an excess of GSH.

Based on the results, it is suggested that the contraction of thoracic aorta and the transient short contraction of femoral and mesenteric arteries are responsible for the transient increase of systolic and diastolic BP and for the transient changes of other 23 APW-Ps. On the other hand, the significantly prolonged relaxation of the mesenteric artery mainly contributed to the prolonged decrease of the systolic and diastolic BP and changes of other APW-Ps. To explain the persistence of the relaxing effect of GSH/SeO_3_^2−^ on mesenteric tension even after the washing out the solution and reapplying NA, it is assumed that the (inter)product(s) of GSH/SeO_3_^2−^ reaction bound relatively tightly to the relevant cellular receptors causing this vessel relaxation, respectively, they significantly affect the physiological character of the relaxation-constrictor properties of the arteries. However, the nature of the active products is still unknown and it is a challenge to be unveiled in future studies. These active products may be potentially suitable for treating hypertension.

## Conclusion

We present evidence that the products of the thiol/SeO_3_^2−^ interaction have numerous effects in vivo, ex vivo and in vitro. These reactive products have significant reducing properties, damage pDNA, decrease rat blood pressure and the tension of mesenteric artery, and modulate numerous rat hemodynamic parameters. The results may explain some of the biological effects of SeO_3_^2–^.

It has mostly been assumed that SeO_3_^2−^ is not an antioxidant by itself, but it is antioxidant when it incorporates into selenoproteins. In our work, we confirmed that SeO_3_^2−^ itself is not an antioxidant, but it has significant reducing properties when interacting with biological thiols, such as GSH, Cys or HCys, that can be found in the cells in significant concentrations. The physiological concentrations of these thiols and supplemented SeO_3_^2−^ in the organism are similar to those used in our study, suggesting that the observed properties of the thiol/SeO_3_^2−^ interaction products could be biologically relevant. This study may contribute to the understanding of many antioxidant effects of SeO_3_^2−^ reported in ex vivo and in vivo. It is known that free radicals contribute to the development of many lifestyle diseases. Therefore, reducing properties of selenite/thiols products can be used in further studies leading to the treatment of pathological conditions caused by oxidative stress.

We found that neither thiols nor SeO_3_^2−^ alone cleaved pDNA in vitro, but SeO_3_^2−^ significantly cleaves pDNA when it interacts with biological thiols such as GSH, Cys or HCys. Additionally, SOD potentiated the pDNA cleavage induced by GSH/SeO_3_^2−^, which indicates that the ^•^O_2_^–^ produced from GSH/SeO_3_^2−^ reaction partially inhibited the pDNA cleavage. The observed reducing and pDNA damaging effects were significantly lower when SeMet was used instead of SeO_3_^2−^, which may contribute to understanding why SeMet is less toxic and exerts less mutagenic effects and DNA cleavage than SeO_3_^2−^. It is suggested that selenide (H_2_Se/HSe^–^) is mainly involved in the thiol/SeO_3_^2−^ reducing effects and hydroxyl radicals generating during H_2_Se oxidation may be responsible for the pDNA damage. However, other reactive (inter)products of thiol/SeO_3_^2−^ interaction may be involved. Product(s) of GSH/SeO_3_^2−^ reaction transiently increased BP and changed several hemodynamic parameters, but they had long-term lowering effects on systolic and diastolic BP, and modulating several other hemodynamic parameters. These observed effects may result from the relative strong binding of unknown active products to receptor(s)/protein(s) regulating the cardiovascular system. Besides, GSH/SeO_3_^2−^ significantly relaxed the segments of isolated resistant mesenteric artery. This fact is suggested to be responsible for the prolonged decrease of the systolic and diastolic BP. As the substances that decrease BP in animal models can be potentially therapeutic agents for the treatment of hypertension, however, the identification of the active products and mechanisms of action in cardiovascular system is an interesting challenge for future research works.

## Supplementary Information

Below is the link to the electronic supplementary material.

Supplementary file1 (PDF 4725 KB)

## Data Availability

All findings and conclusions are based on the presented figures in the main text or in the Supplementary Materials. Original source files can be sent from the corresponding author, Dr. Anton Misak, upon request.
