# Supplementary material for: Products of Selenite/Thiols Interaction Have Reducing Properties, Cleave Plasmid DNA and Decrease Rat Blood Pressure and Tension of Rat Mesenteric Artery
Source: Biol Trace Elem Res. 2024 Apr 27;203(2):903–29. doi: 10.1007/s12011-024-04196-3 (PMC11750908; doi:10.1007/s12011-024-04196-3)
Supplement: Supplementary file 1 — Supplementary file1 (PDF 4725 KB) [file 12011_2024_4196_MOESM1_ESM.pdf]

Biological Trace Element Research

Supplementary Information
to

Products of Selenite/Thiols Interaction Have Reducing Properties, Cleave Plasmid DNA and Decrease Rat Blood Pressure and Tension of Rat Mesenteric Artery

Marian Grman [1], Peter Balis [2], Andrea Berenyiova [2], Helena Svajdlenkova [3,8], Lenka Tomasova [1], Sona Cacanyiova [2], Zuzana Rostakova [4], Iveta Waczulikova [5], Miroslav Chovanec [6], Enrique Domínguez-Álvarez [7], Karol Ondrias [1], Anton Misak [1]

[1] Institute of Clinical and Translational Research, Biomedical Research Center, Slovak Academy of Sciences, Dubravska cesta 9, 845 05 Bratislava, Slovak Republic; anton.misak@savba.sk (A.M.); marian.grman@savba.sk (M.G.); lenka.tomasova@savba.sk (L.T.); karol.ondrias@savba.sk (K.O.)
[2] Institute of Normal and Pathological Physiology, Centre of Experimental Medicine, Slovak Academy of Sciences, Sienkiewiczova 1, 813 71 Bratislava, Slovak Republic; peter.balis@savba.sk (P.B.); andrea.berenyiova@savba.sk (A.B.); sona.cacanyiova@savba.sk (S.C.)
[3] Polymer Institute, Slovak Academy of Sciences, Dubravska cesta 9, 845 41, Bratislava, Slovak Republic; helena.svajdlenkova@savba.sk (H.S.)
[4] Institute of Measurement Science, Slovak Academy of Sciences, Dubravska cesta 9, 841 04 Bratislava, Slovak republic; zuzana.rostakova@savba.sk (Z.R.)
[5] Faculty of Mathematics, Physics and Informatics, Comenius University, Mlynska dolina F1, 842 48 Bratislava, Slovak Republic; Iveta.Waczulikova@fmph.uniba.sk (I.W.)
[6] Cancer Research Institute, Biomedical Research Center, Slovak Academy of Sciences, Dubravska cesta 9, 845 05 Bratislava, Slovak Republic; miroslav.chovanec@savba.sk (M.C.)
[7] Instituto de Química Orgánica General (IQOG), CSIC, Juan de la Cierva 3, 28006 Madrid, Spain; e.dominguez-alvarez@iqog.csic.es (E.D-Á.)
[8] Department of Nuclear Chemistry, Faculty of Natural Science, Comenius University in Bratislava, Ilkovičova 6, 842 15 Bratislava, Slovak Republic; helena.svajdlenkova@uniba.sk (H.S.)

Correspondence: anton.misak@savba.sk

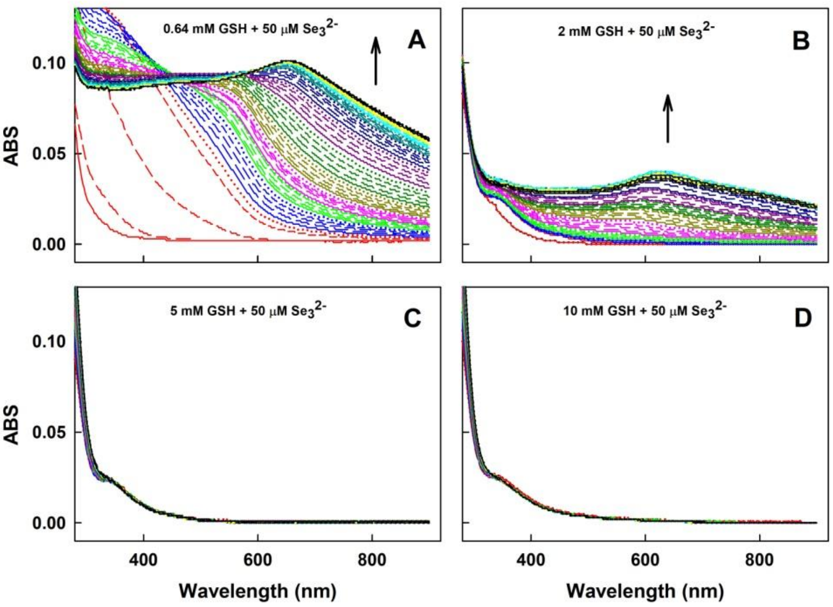

Fig. S1 Representative time-resolved UV-Vis spectra of the interaction of GSH with SeO_3^{2-}. UV-Vis spectra of SeO_3^{2-} (50 µmol L^{-1}) with 640 µmol L^{-1} **(A)**, 2 mmol L^{-1} **(B)**, 5 mmol L^{-1} **(C)** and 10 mmol L^{-1} GSH **(D)**. The spectra were measured every 30 s for 30 min in 100 mmol L^{-1} sodium phosphate, 100 µmol L^{-1} DTPA (pH 7.4 at 37°C). The solid red line indicates the first spectrum measured 30 s after addition of SeO_3^{2-}/GSH, which is followed each 30 s by: long dash red, medium dash red, short dash red, dotted red, solid blue line, long dash blue, medium dash blue, *etc*. Arrows indicate the direction of ABS changes

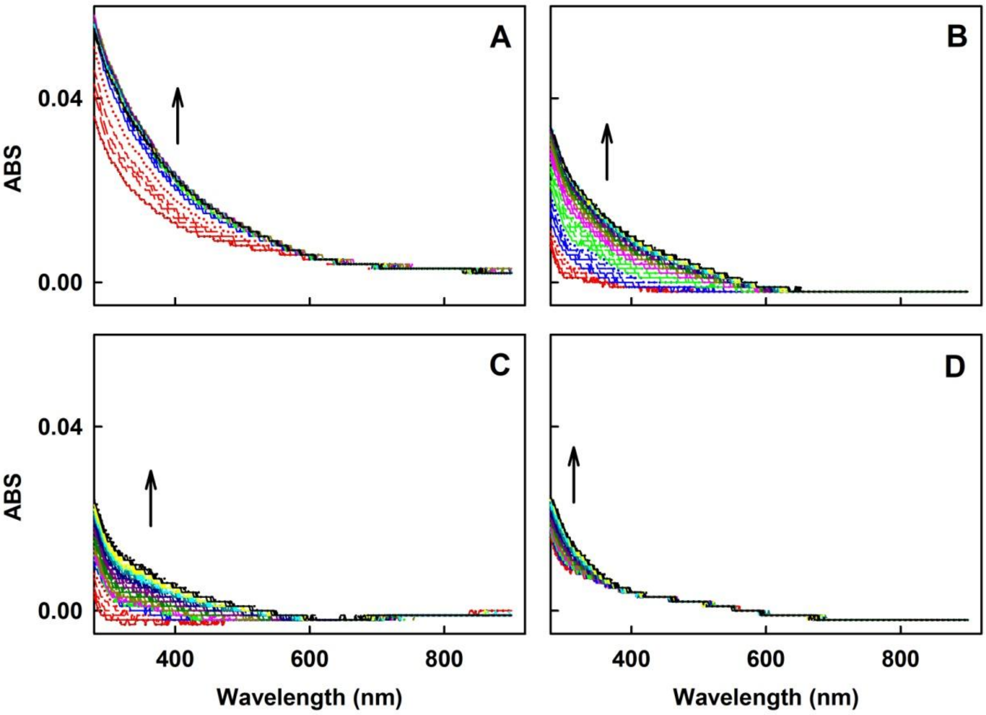

Fig. S2 Representative time-resolved UV-Vis spectra of the interaction of thiol-based compounds with 10 µmol L^{-1} SeO_3^{2-}. UV–Vis spectra of SeO_3^{2-} (10 µmol L^{-1}) with 200 µmol L^{-1} Cys **(A)**, GSH **(B)**, HCys **(C)** and NAC **(D)** were measured every 30 s for 30 min in 100 mmol L^{-1} sodium phosphate, 100 µmol L^{-1} DTPA, pH 7.4, 37°C. The solid red line indicates the first spectrum measured 30 s after addition of SeO_3^{2-}/compounds, which is followed each 30 s by: long dash red, medium dash red, short dash red, dotted red, solid blue line, long dash blue, medium dash blue, *etc*. Arrows indicate direction of ABS changes

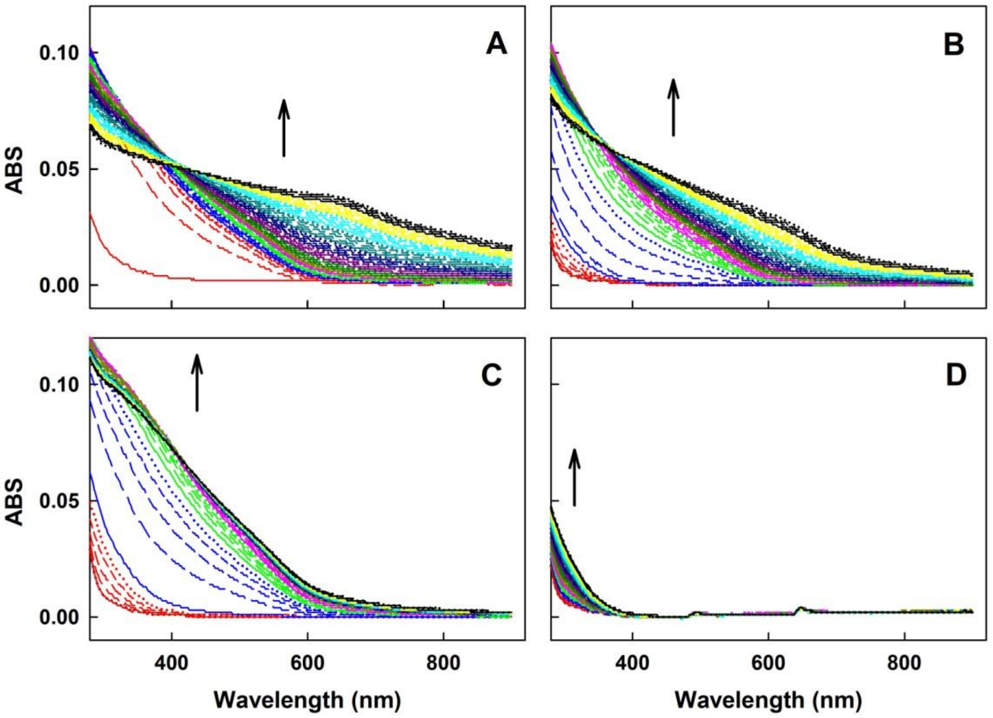

Fig. S3 Representative time-resolved UV-Vis spectra of the interaction of thiol-based compounds with 30 µmol L^{-1} SeO_3^{2-}. UV-Vis spectra of SeO_3^{2-} (30 µmol L^{-1}) with 200 µmol L^{-1} Cys **(A)**, GSH **(B)**, HCys **(C)** and NAC **(D)** were measured every 30 s for 30 min in 100 mmol L^{-1} sodium phosphate, 100 µmol L^{-1} DTPA (pH 7.4 at 37°C). The solid red line indicates the first spectrum measured 30 s after addition of SeO_3^{2-}/compounds, which is followed each 30 s by: long dash red, medium dash red, short dash red, dotted red, solid blue line, long dash blue, medium dash blue, *etc*. Arrows indicate the direction of ABS changes

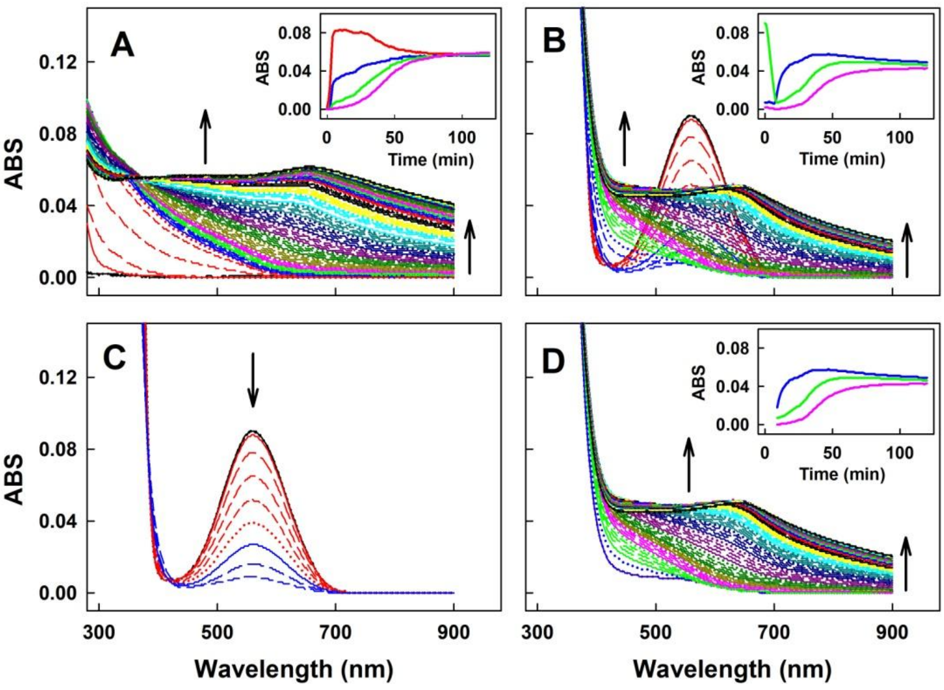

Fig. S4 Representative time-resolved UV-Vis spectra of the interaction of SeO_3^{2-} with GSH in the presence of $^{\bullet}$cPTIO. UV-Vis spectra of GSH (400 µmol L^{-1}) with SeO_3^{2-} (30 µmol L^{-1}) without **(A)** and with **(B)** $^{\bullet}$cPTIO (100 µmol L^{-1}) were measured every

1 min for 120 min in 100 mmol L^{-1} sodium phosphate, 100 µmol L^{-1} DTPA (pH 7.4 at 37°C). Control UV-Vis spectrum of 100 µmol L^{-1} •cPTIO (black, **B**). The solid red line indicates the first spectrum of the mixture, which is followed each 1 min by: long dash red, medium dash red, short dash red, dotted red, solid blue line, long dash blue, medium dash blue, *etc*. Inserts: Time dependency of ABS at 300 (red), 420 (blue), 560 (green) and 700 (pink) nm. The first 8 time-resolved UV-Vis spectra **(C)** and the rest of the spectra **(D)** taken from **(B)**. Arrows indicate the direction of ABS changes

Time dependent UV-Vis spectra of SeO_3^{2-} interacting with GSH without and with •cPTIO

To look for active products of thiol/SeO_3^{2-} interaction reducing •cPTIO we studied changes of UV-Vis spectra of the interaction between GSH/SeO_3^{2-} and •cPTIO. The addition of 30 µmol L^{-1} SeO_3^{2-} into the 400 µmol L^{-1} GSH without (Fig. S4A) and with 100 µmol L^{-1} •cPTIO (Fig. S4B) changed the UV-VIS spectra in time. The time-dependent changes of GSH/SeO_3^{2-} ABS at 300, 420, 560 and 700 nm were different, indicating complex reactions between GSH and SeO_3^{2-} (Fig. S4A and B, Inserts). The time-dependent changes of GSH/SeO_3^{2-} interactions were fast for ~ 0-4 min, then gradually increased (~ 4-70 min) and later (~ 70-120 min) showed no changes in ABS, suggesting no further chemical reactions (Fig. S4A, Insert).

The time-dependent changes of spectra of the GSH/SeO_3^{2-}/•cPTIO mixture showed the fast time-dependent (~ 0-4 min) reduction of •cPTIO (Fig. S4C), followed by fast and slow changes of spectra, respectively (Fig. S4D).

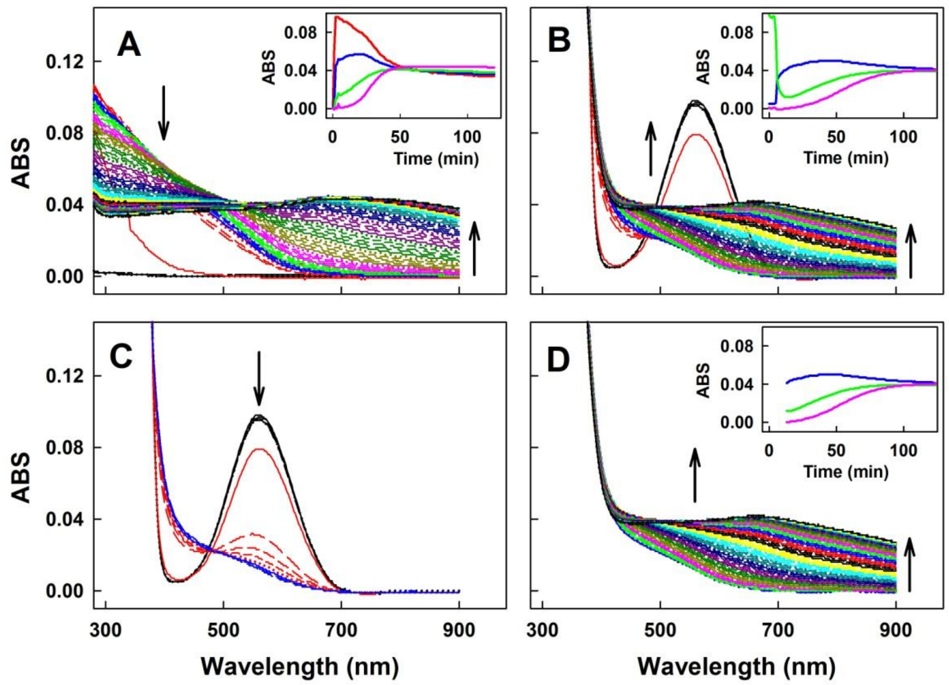

Fig. S5 Representative time-resolved UV-Vis spectra of the interaction of SeO_3^{2-} with Cys in the presence of •cPTIO. UV-Vis spectra of Cys (400 µmol L^{-1}) with SeO_3^{2-} (30 µmol L^{-1}) without **(A)** and with **(B)** •cPTIO (100 µmol L^{-1}) were measured every 1 min for 120 min in 100 mmol L^{-1} sodium phosphate, 100 µmol L^{-1} DTPA (pH 7.4 at 37°C). Control UV-Vis spectrum of 100 µmol L^{-1} •cPTIO (black, **B**). The solid red line indicates the first spectrum of the mixture, which is followed each 1 min by: long dash red, medium dash red, short dash red, dotted red, solid blue line, long dash blue, medium dash blue, *etc*. Inserts: Time-dependences of ABS at 300 (red), 420 (blue), 560 (green) and 700 (pink) nm. The first 8-time-resolved UV-Vis spectra **(C)** and the rest of the spectra **(D)** taken from **(B)**. Arrows indicate the direction of ABS changes

Time dependent UV-Vis spectra of SeO_3^{2-} interacting with Cys without and with •cPTIO:

Similar results, as observed in the case of GSH/SeO_3^{2-} interaction (Fig. S4), were found during interaction of SeO_3^{2-} with Cys without or with •cPTIO. The addition of 30 µmol L^{-1} SeO_3^{2-} into the 400 µmol L^{-1} Cys without (Fig. S5A) and with 100 µmol L^{-1} •cPTIO (Fig. S5B) changed the UV-VIS spectra in time. The time-dependent changes of Cys/SeO_3^{2-} ABS at 300, 420, 560 and 700 nm were different, indicating complex reactions between

Cys and SeO_3^{2-} (Fig. S5A and B, Inserts). The time-dependent changes of spectra of the Cys/SeO_3^{2-}/$^{\bullet}$cPTIO mixtures showed fast time-dependent (~ 0-2 min) reduction of $^{\bullet}$cPTIO (Fig. S5C), the second and the third phases showed fast and slow changes in spectra, respectively (Fig. S5D, Insert).

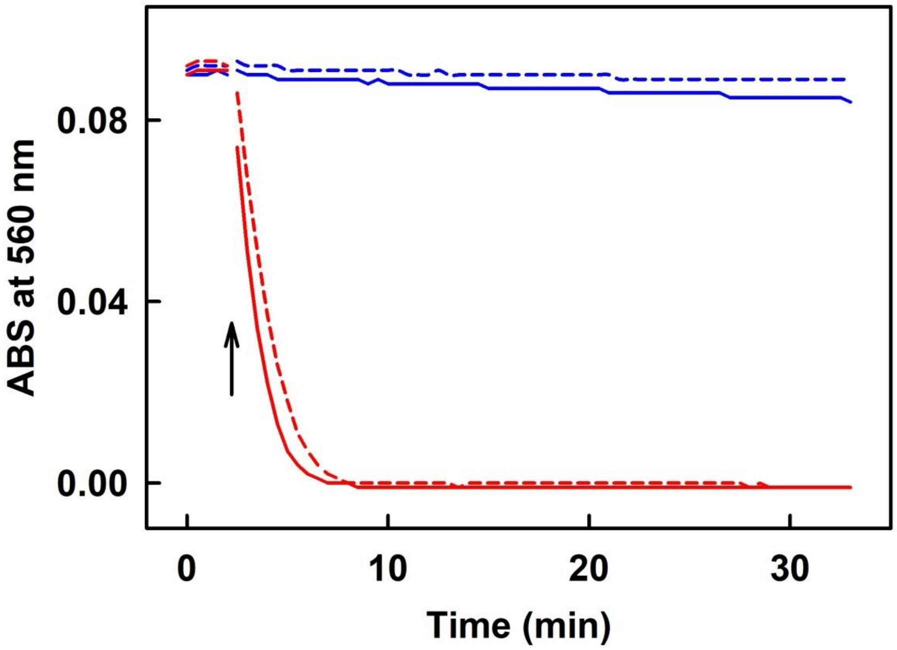

Fig. S6 Time-dependent reduction of $^{\bullet}$cPTIO measured as ABS at 560 nm of UV–Vis spectra during the interaction of SeO_4^{2-} or $SeCl_4$ with GSH. SeO_4^{2-}/$^{\bullet}$cPTIO (3/100 in µmol L^{-1}) in the presence of 5 (dash blue) and 10 mmol L^{-1} (blue) GSH. $SeCl_4$/$^{\bullet}$cPTIO (3/100 in µmol L^{-1}) in the presence of 5 (dash red) and 10 mmol L^{-1} (red) GSH. The samples were measured every 30 s for 30 min in 100 mM sodium phosphate, 100 µM DTPA, pH 7.4, 37°C. Arrow marks the addition of GSH into the SeO_4^{2-}/$^{\bullet}$cPTIO or $SeCl_4$/$^{\bullet}$cPTIO mixtures

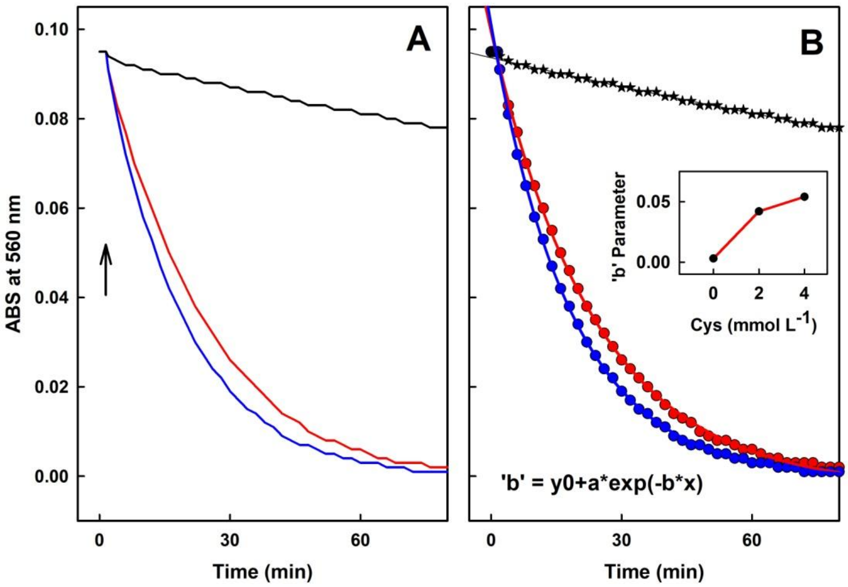

Fig. S7 Evaluation of the rate of $^{\bullet}$cPTIO reduction measured as ABS at 560 nm and induced by different SeO_3^{2-}/Cys molar ratios. **(A)** The UV–Vis spectra during the interaction of 4 mmol L^{-1} Cys with 100 µmol L^{-1} $^{\bullet}$cPTIO (black) and the SeO_3^{2-}/$^{\bullet}$cPTIO (0.2/100 in µmol L^{-1}) with 2 (red) or 4 mmol L^{-1} (blue) Cys. The samples were measured every 30 s for 30 min in 100 mM sodium phosphate, 100 µM DTPA, pH 7.4, 37°C. Arrow marks the addition of compounds into $^{\bullet}$cPTIO. **(B)** Lines: fitted time-dependent data (circles from **A**) using equation of exponential decay: $f = ABS_0 + a*\exp(-b*t)$, where t is time. Insert: Ordinal (concentration) dependence of parameter 'b' calculated from the fitted time-dependent data using equation of exponential decay: $f = ABS_0 + a*\exp(-b*t)$

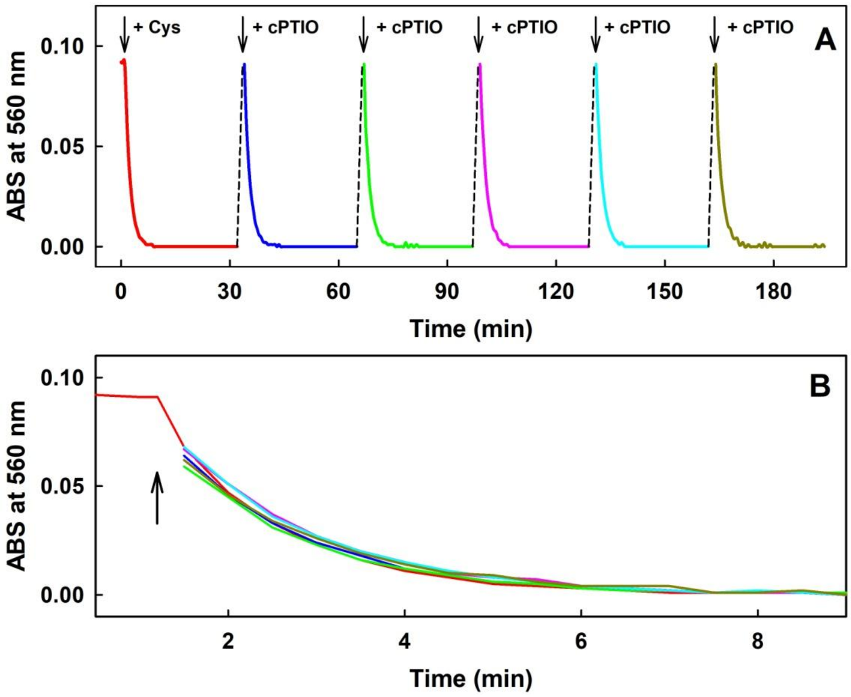

Fig. S8 Repeated reduction of $^{\bullet}$cPTIO measured as ABS at 560 nm of UV-Vis spectra during the interaction of SeO_3^{2-} with L-Cys. **(A)** Reduction of 100 µmol L^{-1} $^{\bullet}$cPTIO with the mixture of L-Cys/SeO_3^{2-} (10 000/3 in µmol L^{-1}) 1 (red) and after adding of 100 µmol L^{-1} $^{\bullet}$cPTIO 5-times in a row (blue, green, pink, cyan and dark yellow). **(B)** Details of the rate of decrease of ABS at 560 nm after 6-times addition of $^{\bullet}$cPTIO into the L-Cys/SeO_3^{2-} (10 000/3 in µmol L^{-1}) mixture. Data are from **(A)**. Additions of L-Cys and $^{\bullet}$cPTIO are marked by arrows

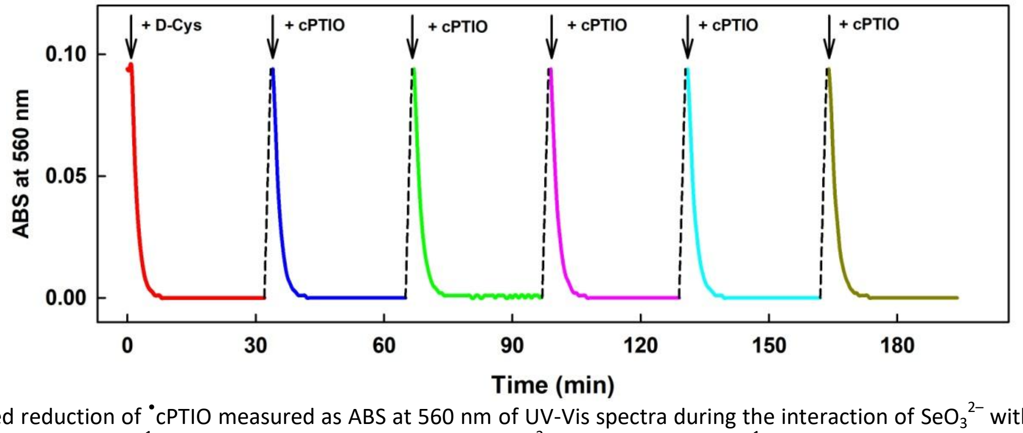

Fig. S9 Repeated reduction of $^{\bullet}$cPTIO measured as ABS at 560 nm of UV-Vis spectra during the interaction of SeO_3^{2-} with D-Cys. Reduction of 100 µmol L^{-1} $^{\bullet}$cPTIO with the mixture of D-Cys/SeO_3^{2-} (10000/3 in µmol L^{-1}, red) and after adding the 100 µmol L^{-1} $^{\bullet}$cPTIO five times in a row (blue, green, pink, cyan and dark yellow; marked by arrows)

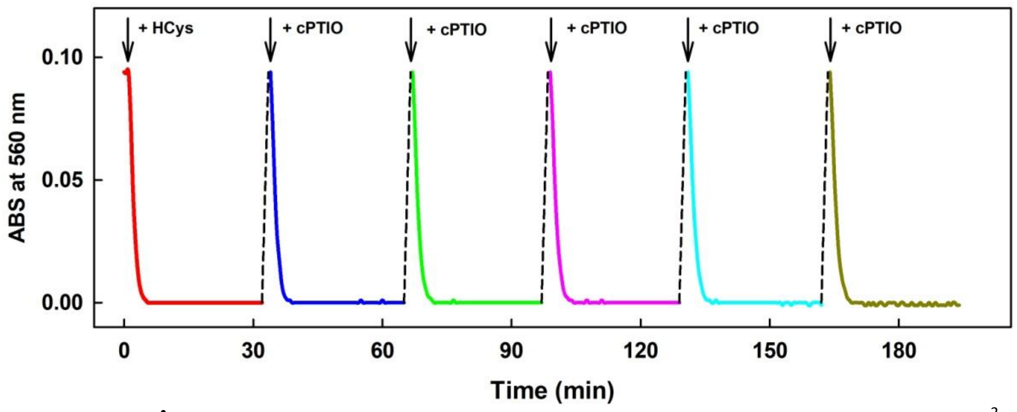

Fig. S10 Repeated reduction of $^{\bullet}$cPTIO measured as ABS at 560 nm of UV-Vis spectra during the interaction of SeO_3^{2-} with HCys. Reduction of 100 µmol L^{-1} $^{\bullet}$cPTIO with the mixture of HCys/SeO_3^{2-} (10000/3 in µmol L^{-1}, red) and after adding the 100 µmol L^{-1} $^{\bullet}$cPTIO five times in a row (blue, green, pink, cyan and dark yellow, marked by arrows)

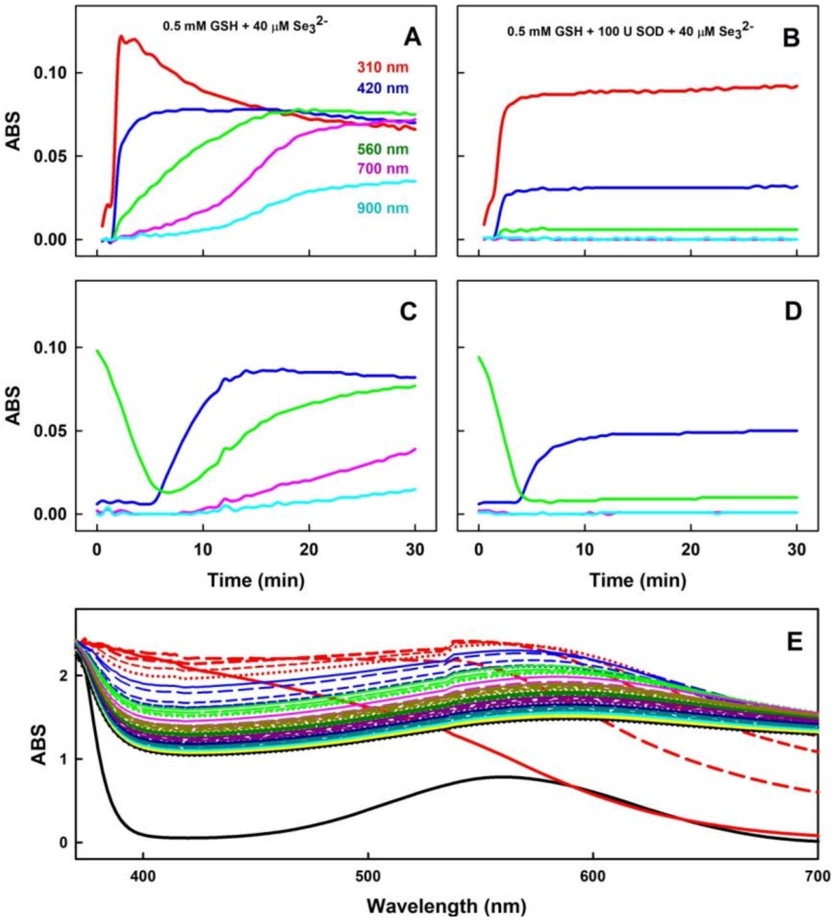

Fig. S11 Time-resolved UV-Vis spectra of GSH/SeO_3^{2-}/$^{\bullet}$cPTIO without and with SOD and spectra of Na_2Se/$^{\bullet}$cPTIO. Time-dependent ABS at 310 nm (red), 420 (blue), 560 (green), 700 (pink) and 900 nm (cyan) of GSH/SeO_3^{2-} (500/40 in µmol L^{-1}) without **(A)** and with 100 Units SOD **(B)**; of $^{\bullet}$cPTIO/GSH/SeO_3^{2-} (100/500/40 in µmol L^{-1}) without **(C)** and with 100 Units SOD **(D)**. Data are taken from Fig. 5. **(E)** UV-Vis spectrum of 850 µmol L^{-1} $^{\bullet}$cPTIO in the phosphate buffer (black) and the spectra after the $^{\bullet}$cPTIO solution was added to powder Na_2Se (≤ 0.2 mg). The first spectrum was measured 4 s after the addition (red), followed by 12 s repetition, long dash red, medium dash red, short dash red, dotted red, solid blue line, long dash blue, medium dash blue, *etc*. Buffer: 100 mmol L^{-1} sodium phosphate, 100 µmol L^{-1} DTPA, pH 7.4, 37°C

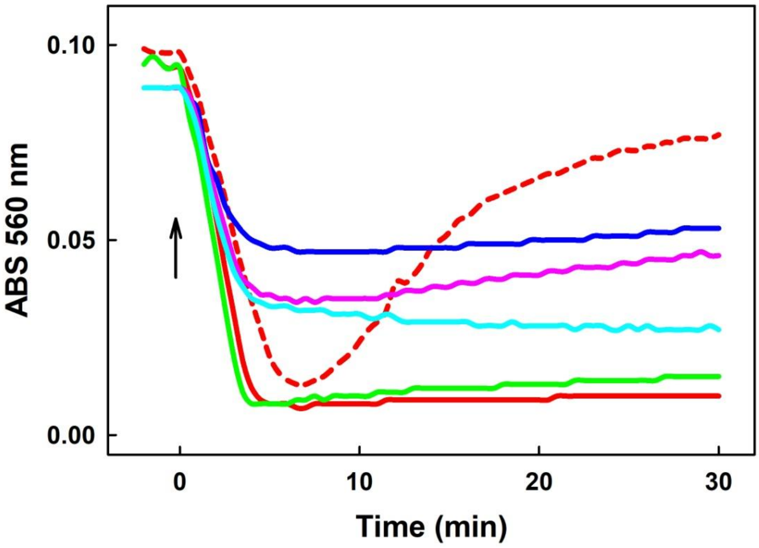

Fig. S12 Time-dependent reduction of 100 µmol L^{-1} $^{\bullet}$cPTIO measured as ABS at 560 nm of UV-Vis spectra during the interaction of SeO_3^{2-} with GSH in the presence of SOD of different activity. The mixture of $^{\bullet}$cPTIO/GSH/SeO_3^{2-} (100/500/40 in µmol L^{-1}) without (dash red) and with 25 (green) and 100 Units SOD (red). The mixture of $^{\bullet}$cPTIO/GSH/SeO_3^{2-} (100/400/100 in µmol L^{-1}) with 50 (pink) and 100 Units SOD (cyan). The mixture of $^{\bullet}$cPTIO/GSH/SeO_3^{2-} (100/400/200 in µmol L^{-1}) with 50 Units SOD (blue). Arrow marks addition of SeO_3^{2-}

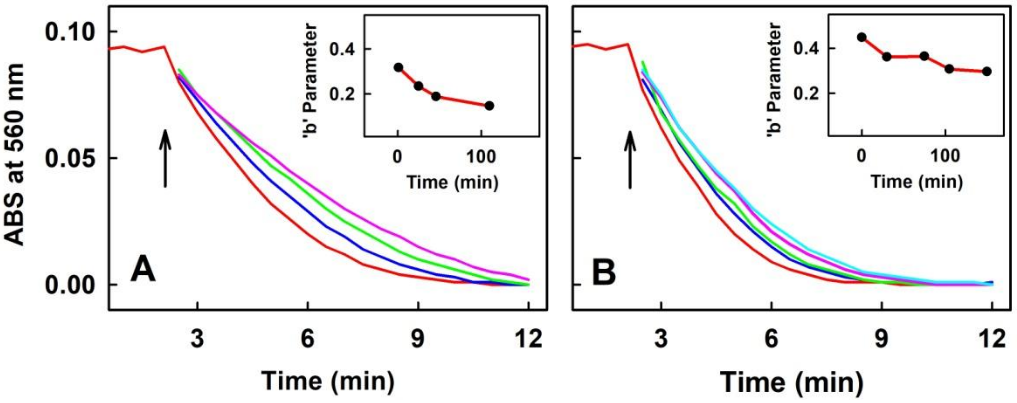

Fig. S13 Evaluation of the rate of $^{\bullet}$cPTIO reduction induced by the mixture of SeO_3^{2-} with GSH stored at different temperature conditions. Stock solution containing 50 mmol L^{-1} GSH and 50 µmol L^{-1} SeO_3^{2-} was prepared by dissolving GSH (1.54 mg) in 100 µL of 50 µmol L^{-1} SeO_3^{2-} in the 100 mmol L^{-1} phosphate buffer (8.5 pH at 37°C). Final pH of GSH/ SeO_3^{2-} was ~ 7.0. Stock solution was either kept at 23 °C **(A)** or frozen at –20°C **(B)** for 24 h. Aliquot (100 µL) of the incubated stock solution was added into the 900 µL of the 100 mmol L^{-1} sodium phosphate, 100 µmol L^{-1} DTPA, 7.4 pH, 37°C buffer (final concentrations 5 mmol L^{-1} GSH and 5 µmol L^{-1} SeO_3^{2-}) and UV–Vis spectra were measured at 30 s intervals. **(A)** Time-dependent reduction of 100 µmol L^{-1} $^{\bullet}$cPTIO with the mixture of GSH/SeO_3^{2-} (final 5000/5 in µmol L^{-1}, stock solution kept 24 h at 23 °C, red) and subsequent addition of the 100 µmol L^{-1} $^{\bullet}$cPTIO three times at 25 (blue), 46 (green) and 110 min (pink). **(B)** Time-dependent reduction of 100 µmol L^{-1} $^{\bullet}$cPTIO with the mixture of GSH/SeO_3^{2-} (final 5000/5 in µmol L^{-1}, stock solution incubated 24 h at –20 °C, red) and subsequent addition of the 100 µmol L^{-1} $^{\bullet}$cPTIO four times at 30 (blue), 75 (green), 105 (pink) and 150 min (cyan). Inserts: Ordinal (time) dependence of parameter 'b' calculated from the fitted time-dependent data **(A)** or **(B)** using equation of exponential decay: $f = Abs_0 + a \times e^{-b \times t}$, where t is time. The fitted parameter '*b*' is in units of min^{-1}

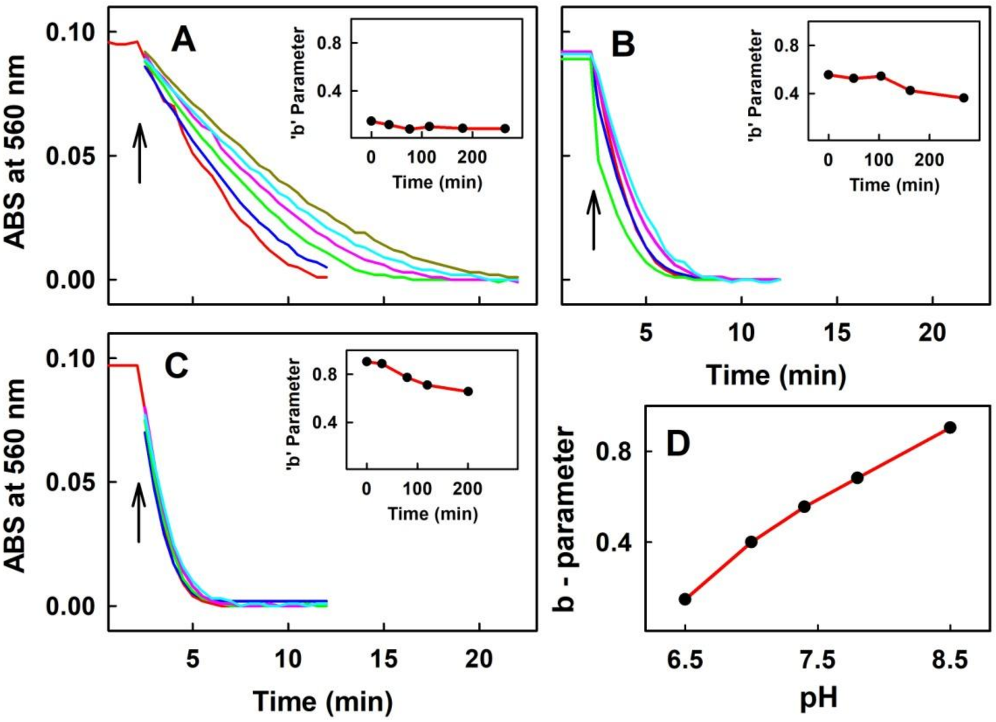

Fig. S14 Evaluation of the rate of $^{\bullet}$cPTIO reduction measured as ABS at 560 nm of UV-Vis spectra during the interaction of SeO_3^{2-} with GSH under different pH conditions. Time-dependent effect of 100 µmol L^{-1} $^{\bullet}$cPTIO with the mixture of GSH/SeO_3^{2-} (5 000/5 in µmol L^{-1}, red) and after adding of 100 µmol L^{-1} $^{\bullet}$cPTIO 5-times in a row (blue, green, pink, cyan and dark yellow). Buffers used: 100 mmol L^{-1} sodium phosphate, 100 µmol L^{-1} DTPA, pH 6.5 **(A)**, 7.4 **(B)** and 8.5 **(C)** at 37°C. Inserts: Ordinal (time) dependence of parameter 'b' calculated from the fitted time-dependent data showed at A, B and C. (D) pH-dependence of parameter 'b' calculated from the fitted time-dependent data of the first $^{\bullet}$cPTIO addition. The data for pH 6.5, 7.4 and 8.5 were taken from **(A)**, **(B)** and **(C)**. Arrows mark the addition of SeO_3^{2-} and GSH into $^{\bullet}$cPTIO. The fitted parameter '*b*' is in units of min^{-1}

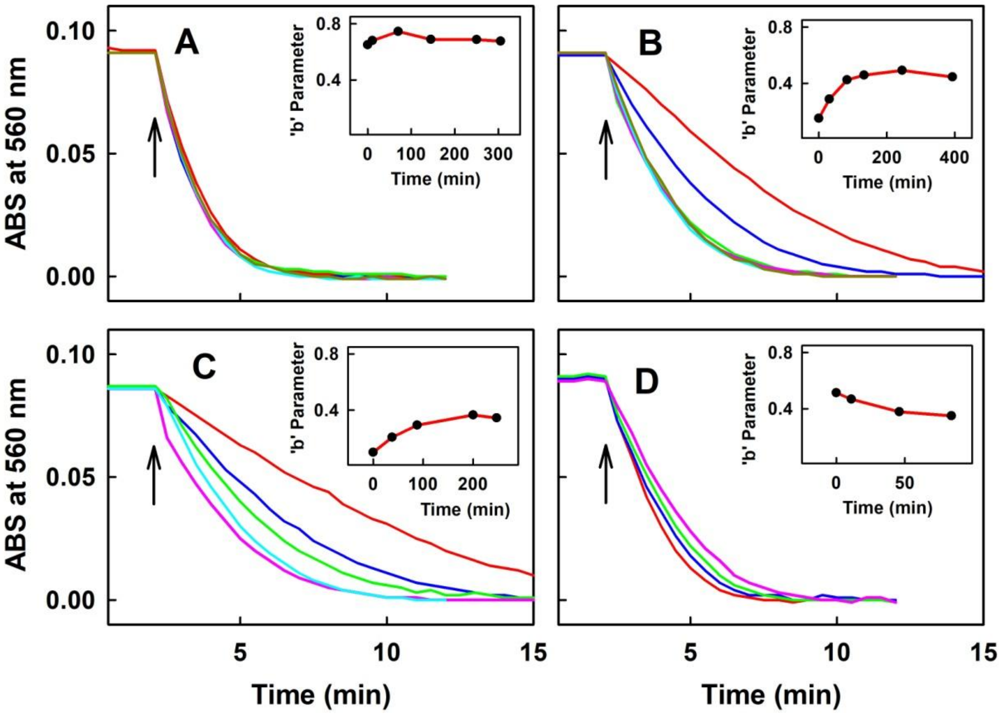

Fig. S15 Evaluation of the rate of •cPTIO reduction induced by the mixture of SeO_3^{2-} with GSH prepared at lower pH. Stock solution containing 100 mmol L^{-1} GSH and 50 µmol L^{-1} SeO_3^{2-} was prepared by dissolving GSH in 100 µL of 50 µmol L^{-1} SeO_3^{2-} in the phosphate buffer (pH 7.4 at 23 °C). Final pH of GSH/ SeO_3^{2-} was ~ 4.5. Aliquot (100 µL) of the stock solution incubated for 2 **(A)**, 27 **(B)** and 86 **(C)** min at 23 °C was added into the 900 µL of sodium phosphate buffer (100 mmol L^{-1} sodium phosphate, 100 µmol L^{-1} DTPA, pH 7.4 at 37°C) (final concentrations: 10 mmol L^{-1} GSH and 5 µmol L^{-1} SeO_3^{2-}) and UV-Vis spectra were measured at 30 s intervals. **(A)** Time-dependent effect of 100 µmol L^{-1} •cPTIO (time 0 min) with the mixture of GSH/SeO_3^{2-} (final 10 000/5 in µmol L^{-1}, red, incubation for 2 min) and after adding of 100 µmol L^{-1} •cPTIO 5-times at 11 (blue), 70 (green), 145 (pink), 250 (cyan) and 305 (dark yellow) min after the first •cPTIO addition. **(B)** Time-dependent effect of 100 µmol L^{-1} •cPTIO with the GSH/SeO_3^{2-} mixture (final 10 000/5 in µmol L^{-1}, red, incubation for 27 min) and after adding of 100 µmol L^{-1} •cPTIO 5-times at 31 (blue), 83 (green), 133 (pink), 245 (cyan) and 293 (dark yellow) min. **(C)** Time-dependent effect of 100 µmol L^{-1} •cPTIO with the GSH/SeO_3^{2-} mixture (final 10 000/5 in µmol L^{-1}, red, incubation for 86 min) and after adding of 100 µmol L^{-1} •cPTIO 4-times at 38 (blue), 88 (green), 200 (pink) and 247 (cyan) min. **(D)** GSH and SeO_3^{2-} was added into 100 µmol L^{-1} •cPTIO in 100 mmol L^{-1} Tris-HCl buffer (pH 7.4 at 37°C) (final concentrations: 5 mmol L^{-1} GSH and 5 µmol L^{-1} SeO_3^{2-}) and UV-Vis spectra were measured at 30 s intervals (red). •cPTIO (100 µmol L^{-1}) was added again into the GSH/SeO_3^{2-} mixture 3-times at 11 (blue), 46 (green) and 84 (pink) min. Inserts: Ordinal (time) dependence of parameter 'b' calculated from the fitted time-dependent data **(A)**, **(B)**, **(C)** or **(D)** using equation of exponential decay: $f = Abs_0 + a \times e^{-b \times t}$, where t is time. Arrows mark the addition of SeO_3^{2-} and GSH into •cPTIO. The fitted parameter '*b*' is in units of min^{-1}

Stability and potency of incubated GSH/SeO_3^{2-} stock solution was studied at final ~ pH 4.5

Stock solution of 100 mmol L^{-1} GSH with 50 µmol L^{-1} SeO_3^{2-} (final pH ~ 4.5 at 23 °C) was prepared and after 2, 27 and 86 min of incubation, 100 µL of the stock solution was added into 900 µL of 100 µmol L^{-1} •cPTIO in phosphate buffer (pH 7.4 at 37°C) (Fig. S15). The addition of the 2 min-incubated stock solution into •cPTIO and subsequent next 5-time •cPTIO additions reduced •cPTIO solution at the same rate (Fig. S15A). However, after the addition of the 27 min-incubated stock solution, the rate of •cPTIO reduction was slow, but it increased after further •cPTIO additions, when the solution was incubated at 37°C for 31 and 83 min. Further incubation did not increase the •cPTIO reduction (Fig. S15B). Incubation of the stock solution for 86 min caused the formation of species, which were even less active in the •cPTIO reduction than those formed after 27 min incubation (Fig. S15C). Similarly, as in the previous case (Fig. S15B), the rate of the •cPTIO reduction increased in time during the incubation of measured solution at 37°C, but the final rates were lower than in samples, in which the stock solution was incubated for 2 or 27 min (Fig.s S15A and B).

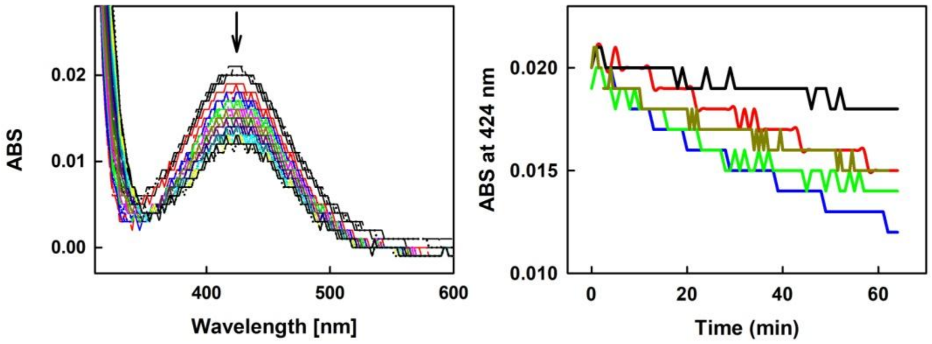

Fig. S16 Representative time-resolved UV-Vis spectra of the interaction of GSH/SeO_3^{2-} with TEMPO. **(A)** Spectra of TEMPO (2 mmol L^{-1}) alone (black) and after addition of GSH (10 mmol L^{-1}) with 50 µmol L^{-1} SeO_3^{2-}. The spectra were measured every 1 min for 60 min in 100 mmol L^{-1} sodium phosphate, 100 µmol L^{-1} DTPA (pH 7.4 at 37°C). The solid red line indicates the first spectrum measured 30 s after the addition of SeO_3^{2-}/GSH into the TEMPO solution, which is followed each 1 min by: long dash red, medium dash red, short dash red, dotted red, solid blue line, long dash blue, medium dash blue, *etc*. **(B)** Time-dependent reduction of TEMPO (2 mmol L^{-1}) measured as ABS at 424 nm of the UV-Vis spectra during the interaction of GSH (10 mmol L^{-1}) with 0 (black), 20 (dark yellow), 30 (red), 50 (blue) and 100 (green) µmol L^{-1} SeO_3^{2-}

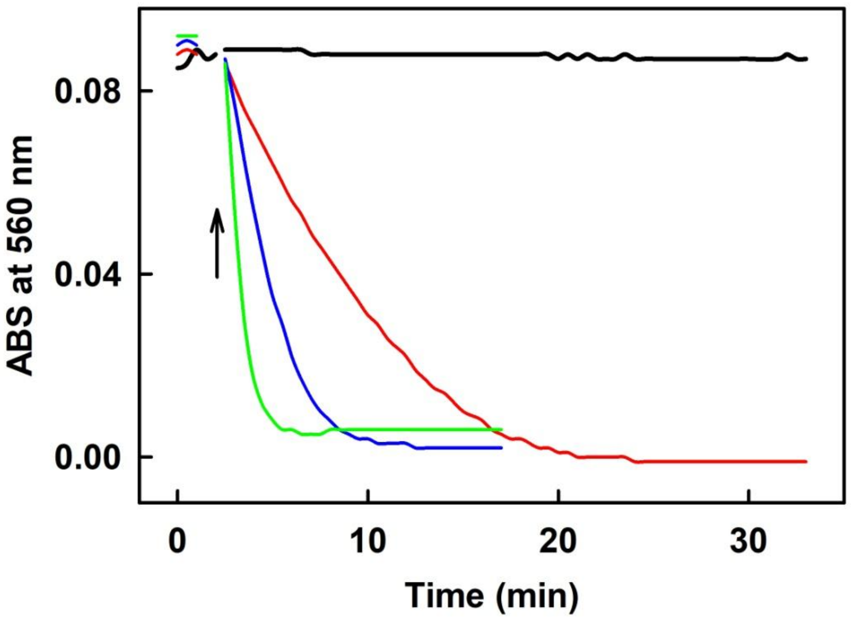

Fig. S17 Reduction of $^{\bullet}$cPTIO measured as ABS at 560 nm of UV–Vis spectra during the interaction of 400 µmol L^{-1} Cys with different concentrations of SeO_3^{2-}. The 100 µmol L^{-1} $^{\bullet}$cPTIO with 400 µmol L^{-1} Cys (black). The mixture of Cys/$^{\bullet}$cPTIO (400/100 in µmol L^{-1}) with 1 (red), 3 (blue) and 10 µmol L^{-1} (green) SeO_3^{2-}. The samples were measured every 30 s for 30 min in 100 mM sodium phosphate, 100 µM DTPA, pH 7.4, 37°C. Arrow marks the addition of compounds into $^{\bullet}$cPTIO

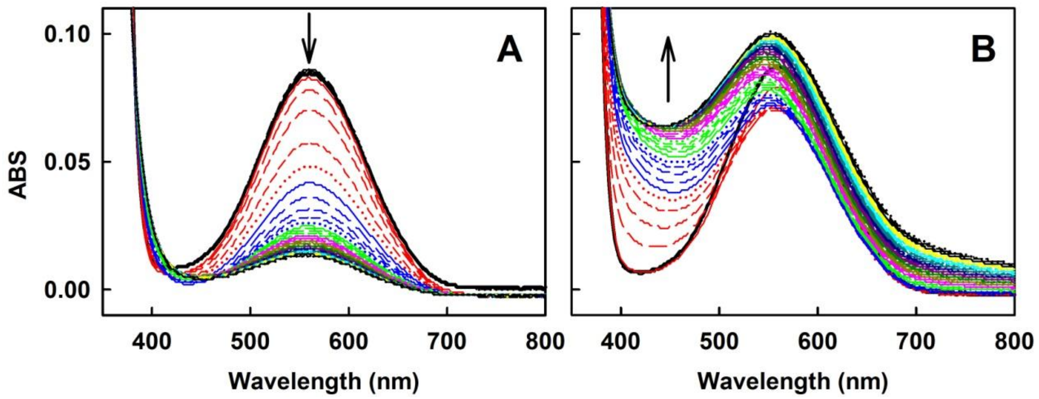

Fig. S18 Reduction of $^{\bullet}$cPTIO measured as ABS at 560 nm of UV–Vis spectra during the interaction of 200 µmol L^{-1} Cys with different concentrations of SeO_3^{2-}. Time-resolved UV-Vis spectra of the interaction of Cys/$^{\bullet}$cPTIO (200/100 in µmol L^{-1}) in the presence of 10 **(A)** and 100 µmol L^{-1} **(B)** SeO_3^{2-}. The spectra were measured every 30 s for 30 min in 100 mmol L^{-1} sodium phosphate, 100 µmol L^{-1} DTPA, pH 7.4, 37°C. The black line indicates the spectrum of $^{\bullet}$cPTIO. The solid red line indicates the first spectrum measured 30 s after the addition of SeO_3^{2-} to the Cys/$^{\bullet}$cPTIO solution, which is followed each 30 s by: long dash red, medium dash red, short dash red, dotted red, solid blue line, long dash blue, medium dash blue, *etc*. Arrows indicate direction of ABS changes

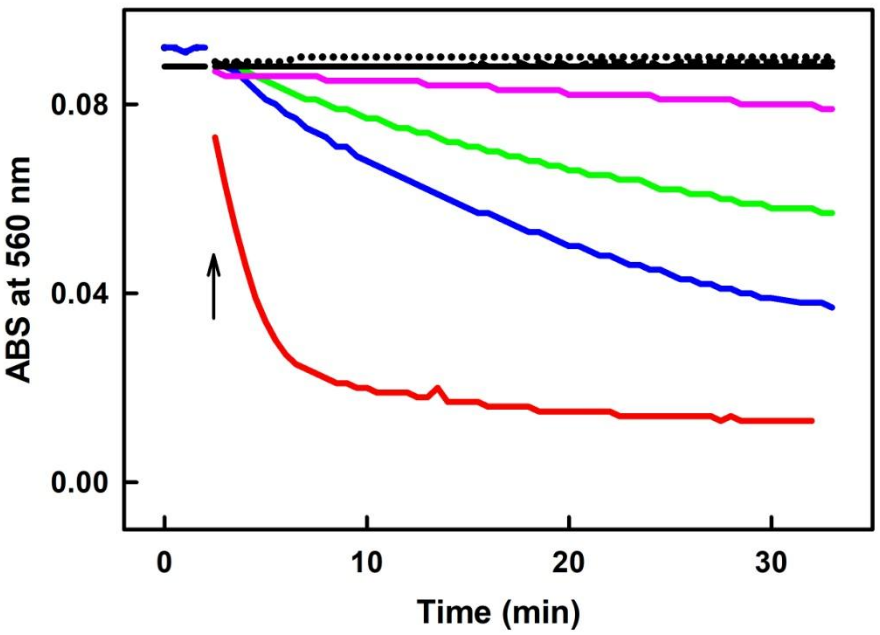

Fig. S19 Time-dependent reduction of •cPTIO measured as ABS at 560 nm of UV-Vis spectra during the interaction of SeO_3^{2-} with thiol-based compounds. SeO_3^{2-}/•cPTIO (10/100 in µmol L^{-1}) with 200 µmol L^{-1} of the thiol-based compounds: Cys (red), HCys (blue), GSH (green), NAC (pink), cystine (black), GSSG (dash black) and MET (dotted black). Arrow marks the addition of compounds into •cPTIO

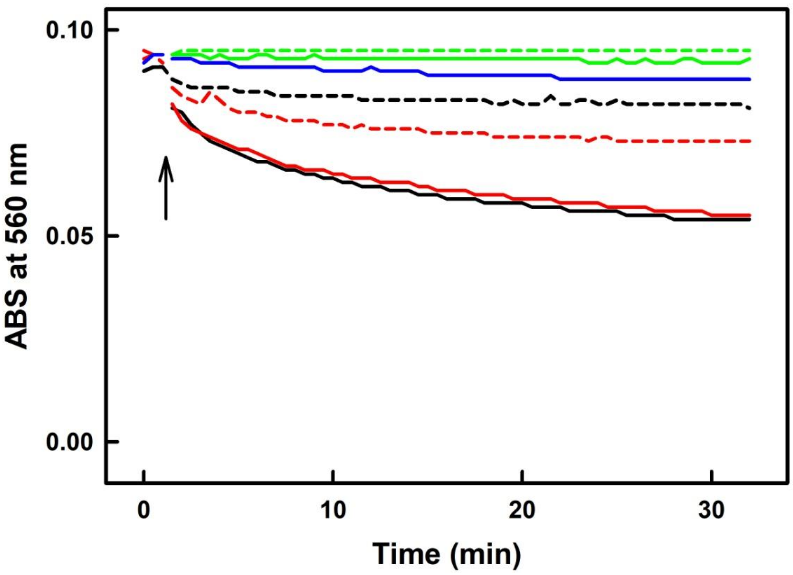

Fig. S20 Time-dependent reduction of •cPTIO measured as ABS at 560 nm of UV-Vis spectra during the interaction of GSH and Trolox. •cPTIO (100 µmol L^{-1}) with 10 (dash black) or 100 µmol L^{-1} (black) Trolox, or with 400 (dash green) or 5 000 µmol L^{-1} (green) GSH. •cPTIO /GSH (100/400 in µmol L^{-1}) with 30 (dash red) or 100 µmol L^{-1} (red) Trolox. •cPTIO /GSH (100/5 000 in µmol L^{-1}) with 5 µmol L^{-1} (blue) Trolox. Arrow marks the addition of compounds into •cPTIO

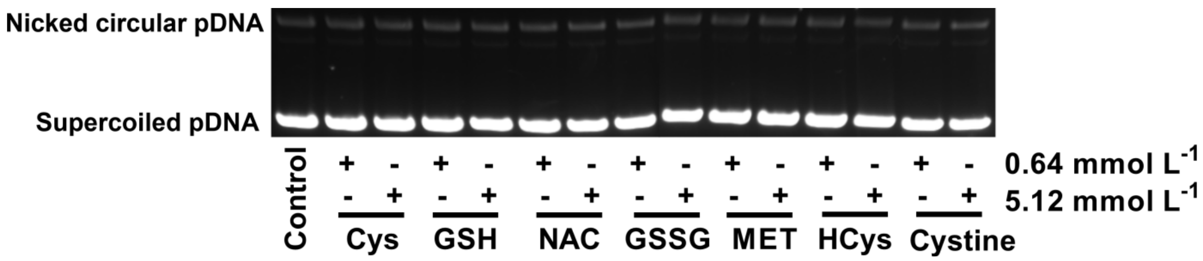

Fig. S21 The pDNA cleavage potency of the compounds used *per se* in a 25 mmol L^{-1} sodium phosphate buffer and 50 µmol L^{-1} DTPA at 37°C. The band at the bottom corresponds to the circular supercoiled form of pDNA and the top band corresponds to the nicked circular form. Control is without any treatment. The final concentration of pDNA was 0.2 µg in 20 µL. The thiols were used at concentration of 0.64 or 5.12 mmol L^{-1}

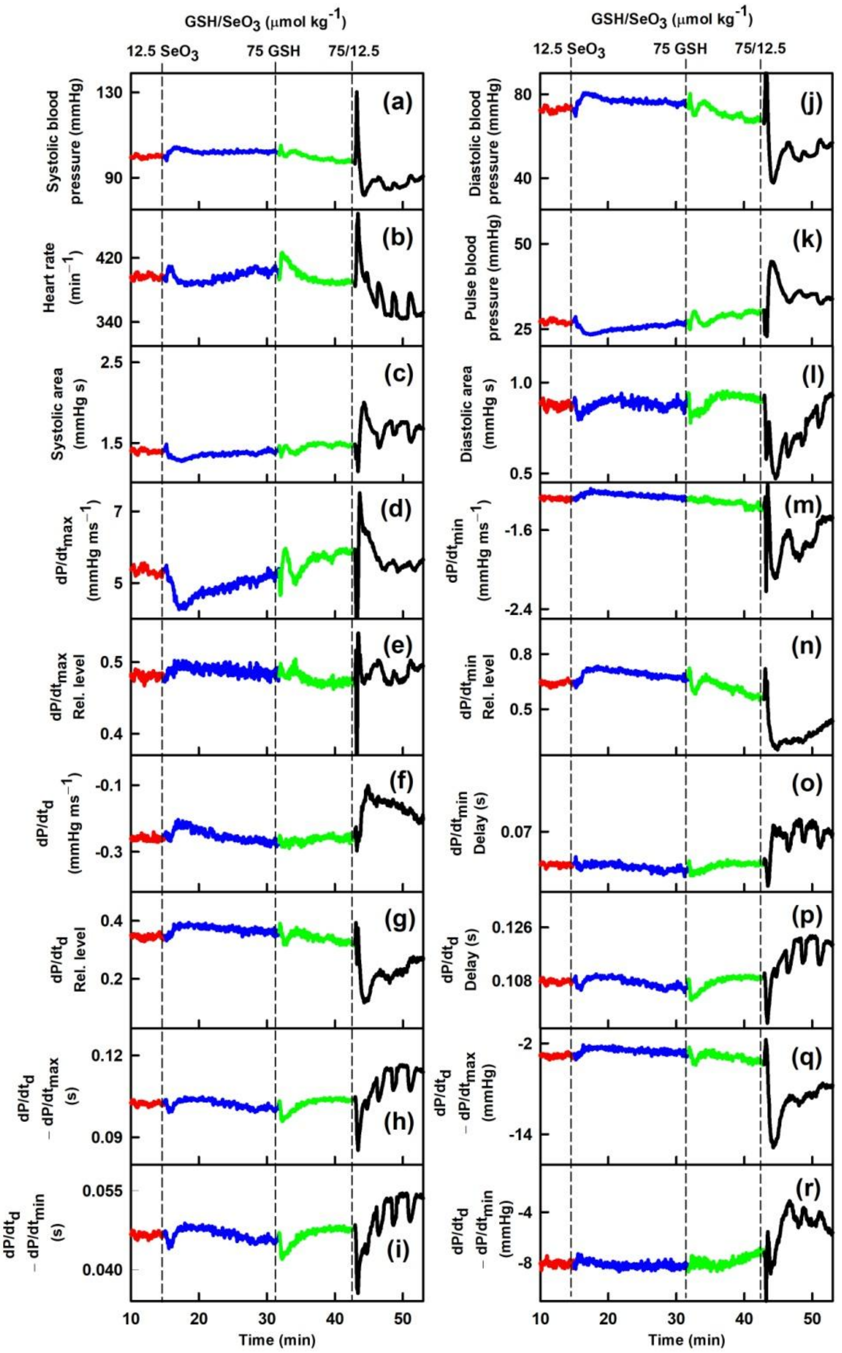

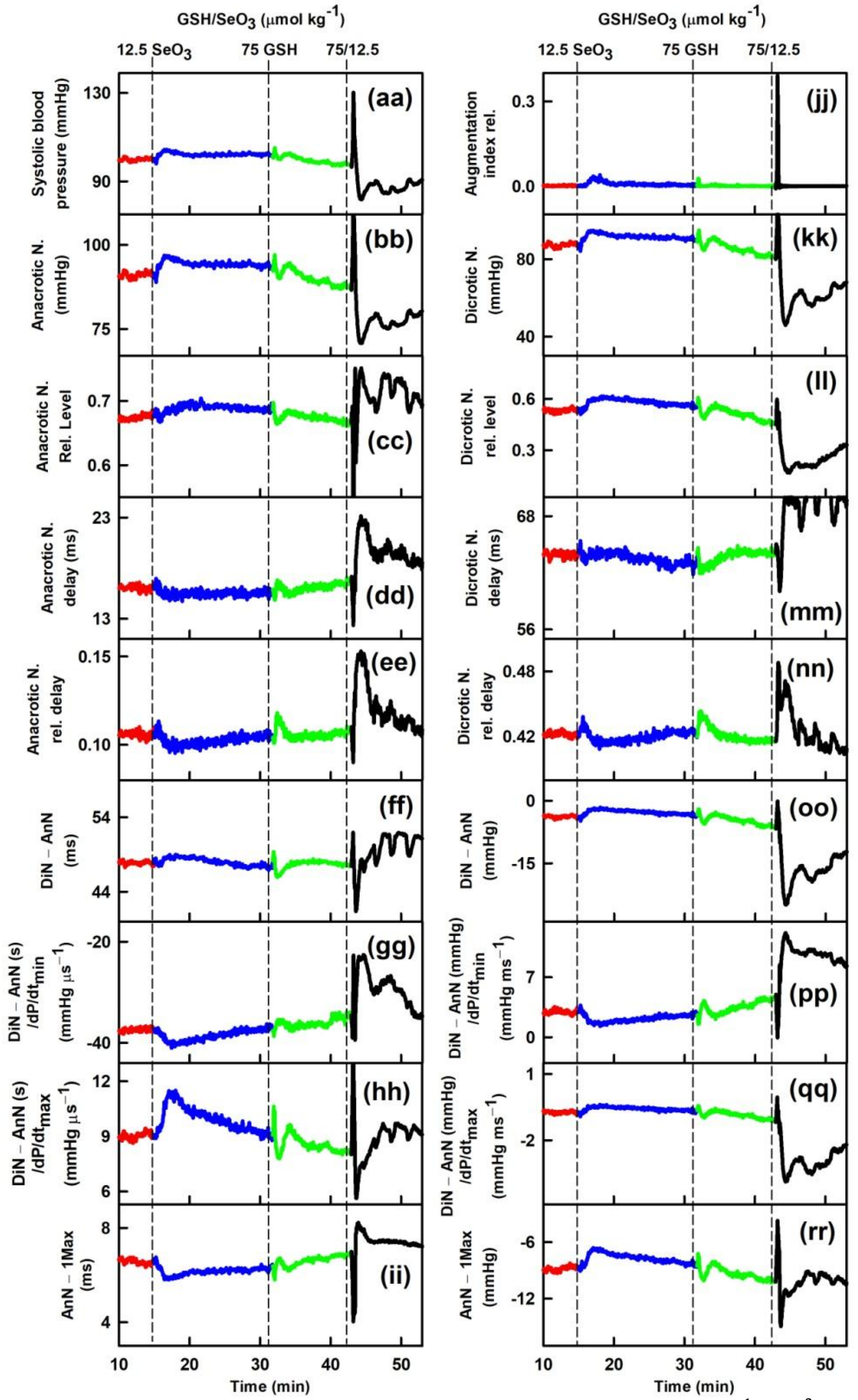

Fig. S22 Time-dependent changes in rat 35 APW-Ps after IV administration of 12.5 μmol kg^{-1} SeO_3^{2-}, 75 μmol kg^{-1} GSH and the GSH/SeO_3^{2-} (75/12.5 in μmol L^{-1}) mixture. Vertical black dashed lines show the start of compound IV administration for 15 s. Definitions, units and abbreviations of APW-Ps evaluated from the APW are as explained previously [1,2]

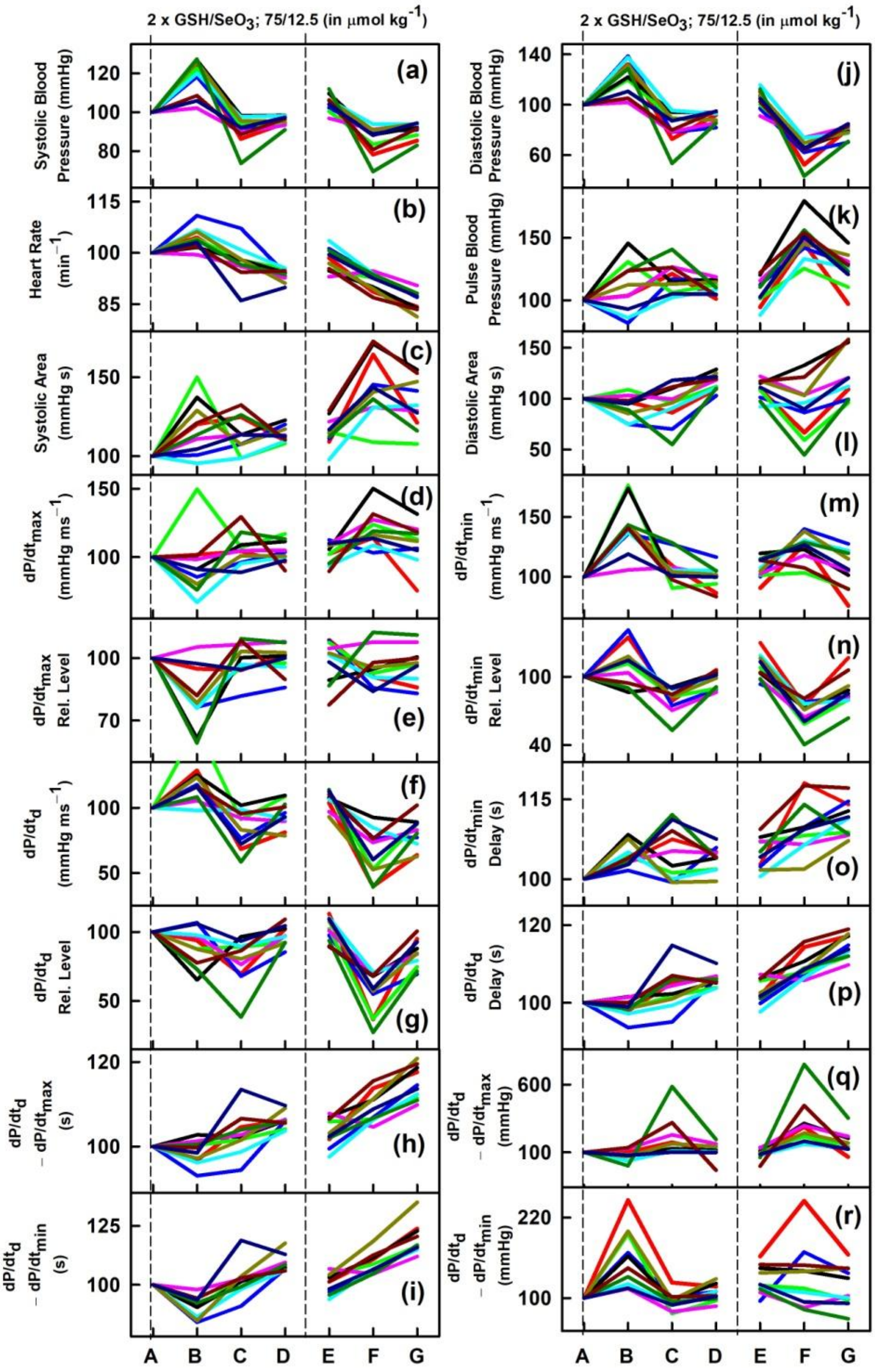

2 x GSH/SeO$_3$; 75/12.5 (in μmol kg^{-1})
Systolic Blood Pressure (mmHg)
Heart Rate (min^{-1})
Systolic Area (mmHg s)
dP/dt$_{max}$ (mmHg ms^{-1})
dP/dt$_{max}$ Rel. Level
dP/dt$_d$ (mmHg ms^{-1})
dP/dt$_d$ Rel. Level
dP/dt$_d$ – dP/dt$_{max}$ (s)
dP/dt$_d$ – dP/dt$_{min}$ (s)
Diastolic Blood Pressure (mmHg)
Pulse Blood Pressure (mmHg)
Diastolic Area (mmHg s)
dP/dt$_{min}$ (mmHg ms^{-1})
dP/dt$_{min}$ Rel. Level
dP/dt$_{min}$ Delay (s)
dP/dt$_d$ Delay (s)
dP/dt$_d$ – dP/dt$_{max}$ (mmHg)
dP/dt$_d$ – dP/dt$_{min}$ (mmHg)
(a) (b) (c) (d) (e) (f) (g) (h) (i) (j) (k) (l) (m) (n) (o) (p) (q) (r)
A B C D E F G

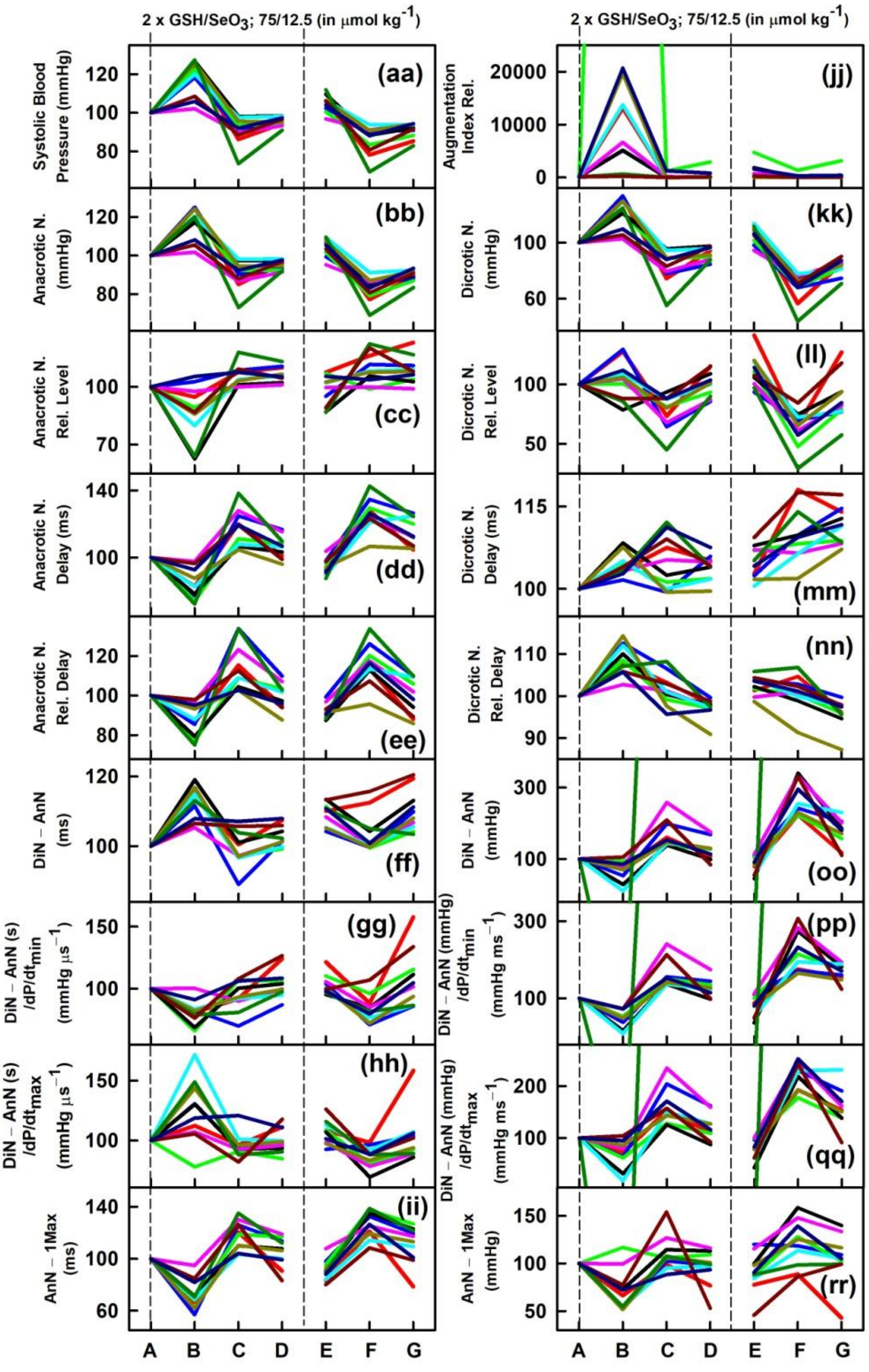

Fig. S23 Relative effect of GSH/SeO_3^{2-} (75/12.5 µmol kg^{-1}) mixture incubated 30 s at 23 ± 1 °C on 35 APW-Ps. **(A)** Value of APW-Ps before administration of the mixture (control, 100%). The first administration: **(B)** Effect of the mixture on 35 APW-Ps taken at time of maximum effect on systolic BP, i.e. at time of maximal systolic BP value (see Fig. 14; (a) or (aa), in %). **(C)** Effect of the mixture on 35 APW-Ps at time of minimum effect on systolic BP (%), i.e. at time of minimum systolic BP value. **(D)** Effect of the mixture on 35 APW-Ps acquired in 7th min after GSH/SeO_3^{2-} administration (%). The second administration: **(E)**, **(F)** and **(G)** have the same meaning as **(B)**, **(C)** and **(D)**, respectively. Number of experiments N = 10. Each rat represents different color. Definitions, units and abbreviations of APW-Ps evaluated from the APW are as explained previously [1,2]

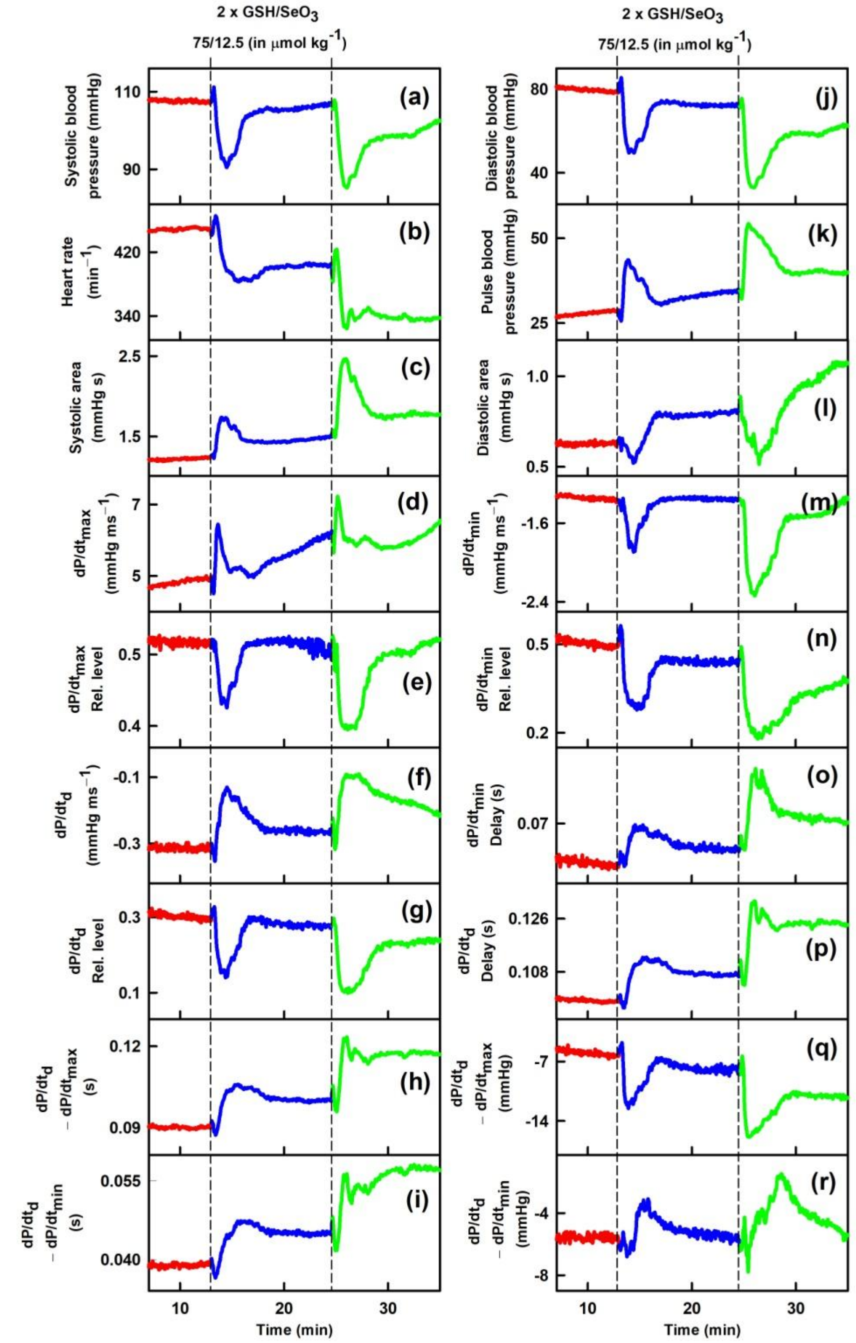

2 x GSH/SeO3
75/12.5 (in μmol kg-1)
(a)
Systolic blood pressure (mmHg)
(b)
Heart rate (min-1)
(c)
Systolic area (mmHg s)
(d)
dP/dtmax (mmHg ms-1)
(e)
dP/dtmax Rel. level
(f)
dP/dtd (mmHg ms-1)
(g)
dP/dtd Rel. level
(h)
dP/dtd – dP/dtmax (s)
(i)
dP/dtd – dP/dtmin (s)
Time (min)
(j)
Diastolic blood pressure (mmHg)
(k)
Pulse blood pressure (mmHg)
(l)
Diastolic area (mmHg s)
(m)
dP/dtmin (mmHg ms-1)
(n)
dP/dtmin Rel. level
(o)
dP/dtmin Delay (s)
(p)
dP/dtd Delay (s)
(q)
dP/dtd – dP/dtmax (mmHg)
(r)
dP/dtd – dP/dtmin (mmHg)

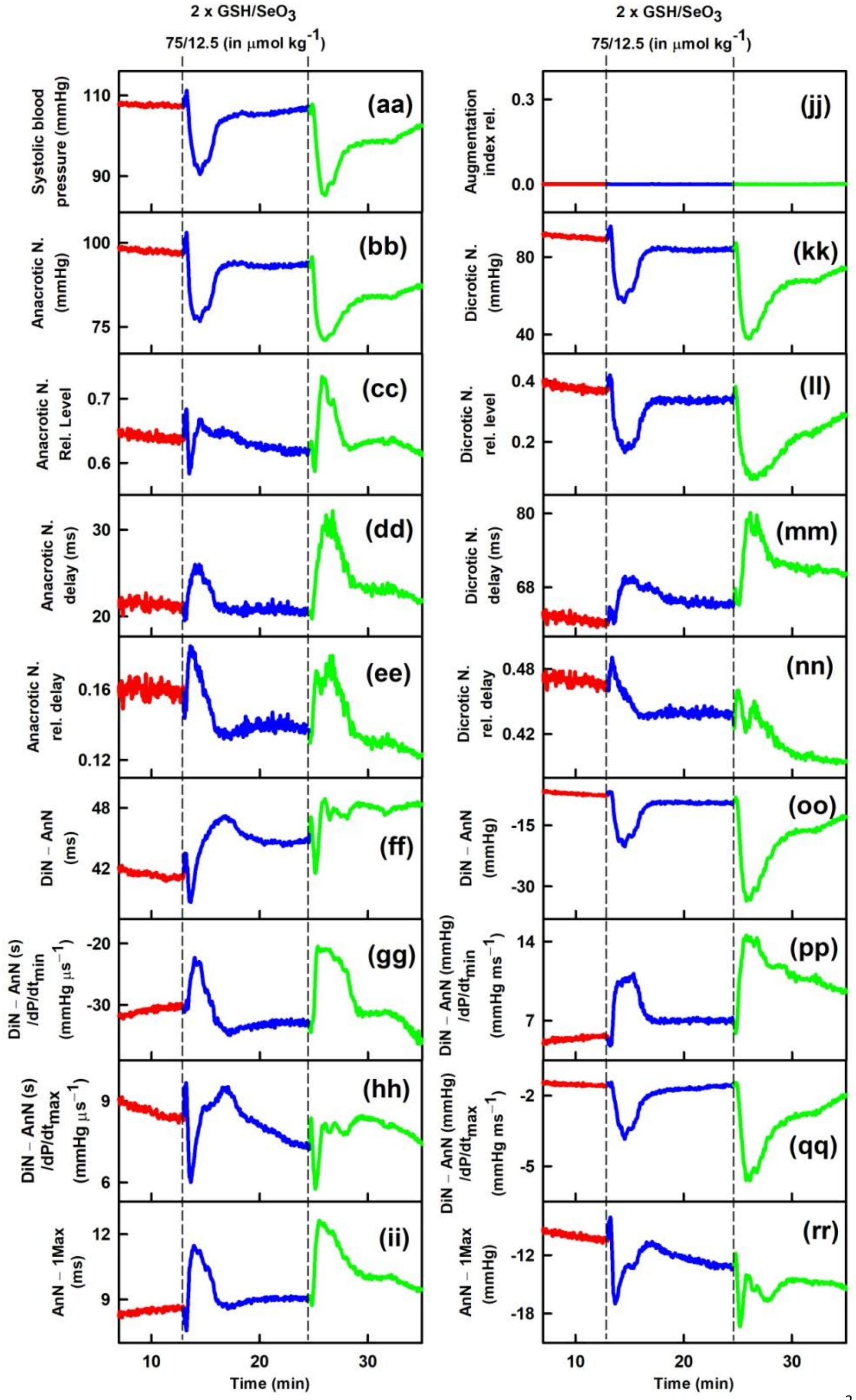

Fig. S24 Time-dependent changes in rat 35 APW-Ps after two times IV administration of the GSH/SeO_3^{2-} mixture incubated 90 s at 23 ± 1 °C. Theoretical initial concentration of GSH/SeO_3^{2-} was calculated according to the rat weight as 75/12.5 µmol kg-1. Vertical black dashed lines show the start of the mixture administration for 15 s. Definitions, units and abbreviations of APW-Ps evaluated from the APW are as explained previously [1,2]

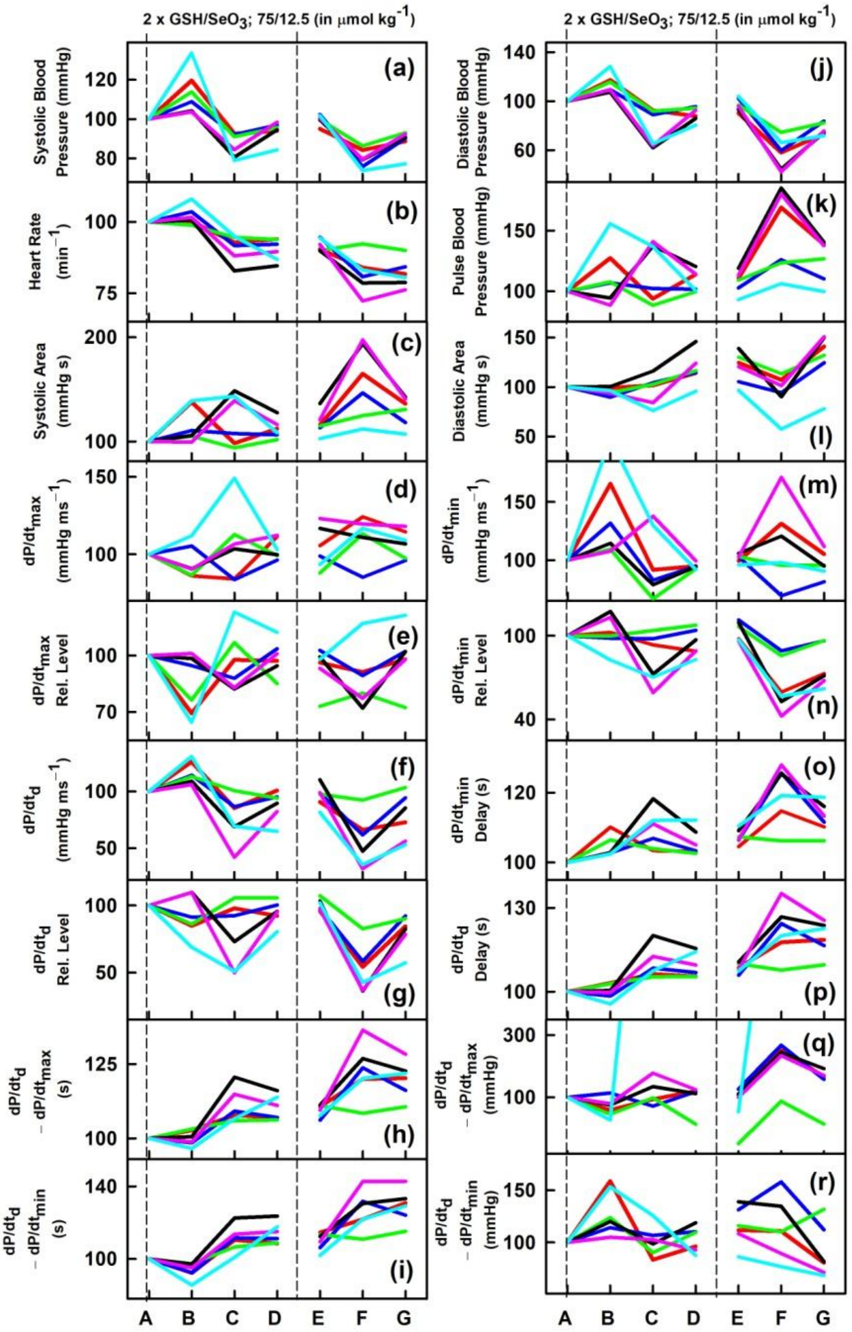
2 x GSH/SeO3; 75/12.5 (in μmol kg-1)
Systolic Blood Pressure (mmHg)
(a)
Heart Rate (min-1)
(b)
Systolic Area (mmHg s)
(c)
dP/dtmax (mmHg ms-1)
(d)
dP/dtmax Rel. Level
(e)
dP/dtd (mmHg ms-1)
(f)
dP/dtd Rel. Level
(g)
dP/dtd - dP/dtmax (s)
(h)
dP/dtd - dP/dtmin (s)
(i)
Diastolic Blood Pressure (mmHg)
(j)
Pulse Blood Pressure (mmHg)
(k)
Diastolic Area (mmHg s)
(l)
dP/dtmin (mmHg ms-1)
(m)
dP/dtmin Rel. Level
(n)
dP/dtmin Delay (s)
(o)
dP/dtd Delay (s)
(p)
dP/dtd - dP/dtmax (mmHg)
(q)
dP/dtd - dP/dtmin (mmHg)
(r)
A B C D E F G

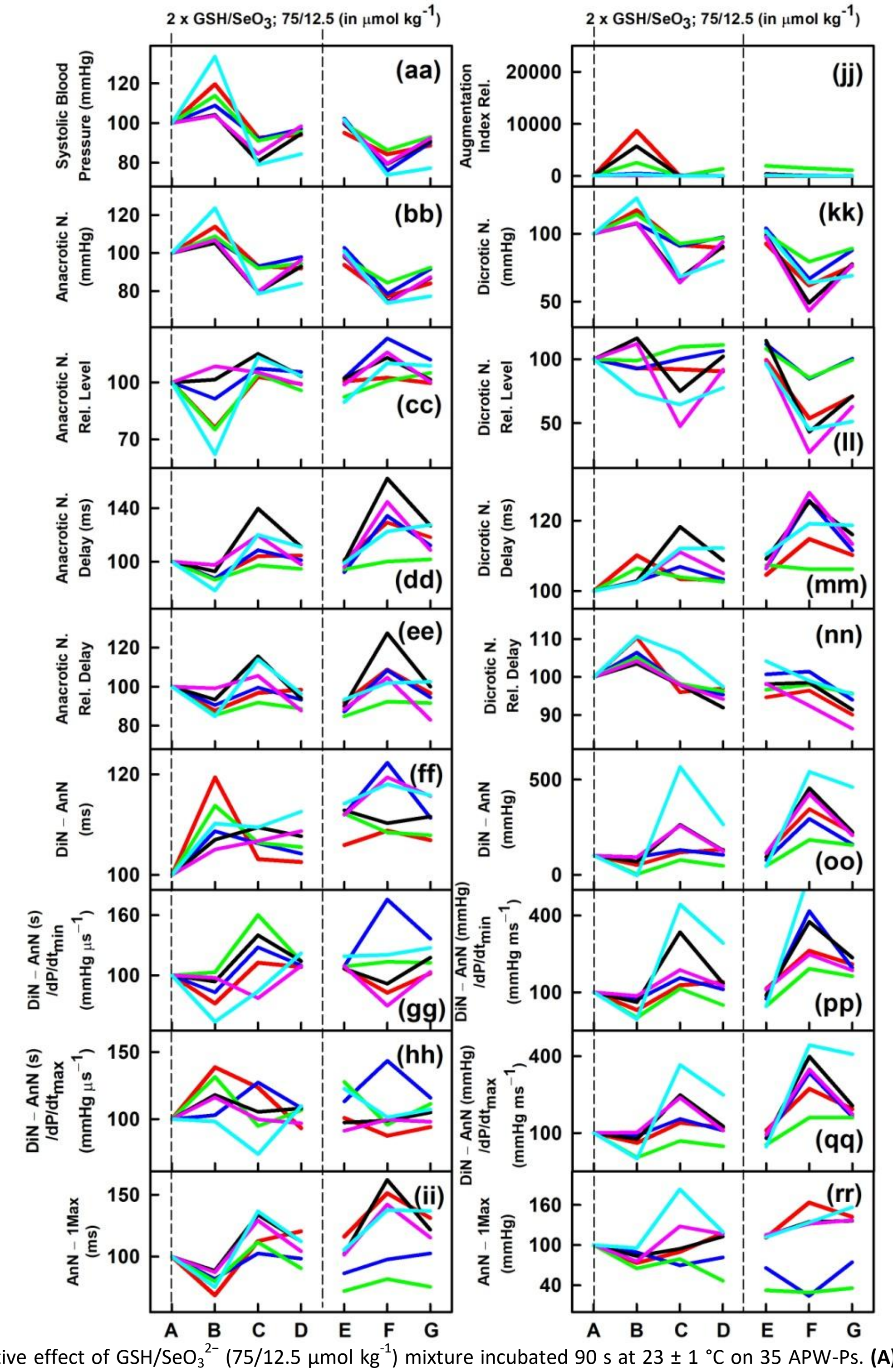

Fig. S25 Relative effect of GSH/SeO_3^{2-} (75/12.5 μmol kg^{-1}) mixture incubated 90 s at 23 ± 1 °C on 35 APW-Ps. **(A)** Value of APW-Ps before mixture administration (control, 100%). The first administration: **(B)** Effect of the mixture on 35 APW-Ps taken at time of maximum effect on systolic BP, i.e. at time of maximal systolic BP value (see Fig. S24; (a) or (aa), in %). **(C)** Effect of the mixture on 35 APW-Ps at time of minimum effect on systolic BP (%), i.e. at time of minimum systolic BP value. **(D)** Effect of the mixture on 35 APW-Ps acquired at 7th min after administration (%).The second administration: **(E)**, **(F)** and **(G)** have the same meaning as **(B)**, **(C)** and **(D)**, respectively. Number of experiments N = 6. Each rat represents different color. Definitions, units and abbreviations of APW-Ps evaluated from the APW are as explained previously [1,2]

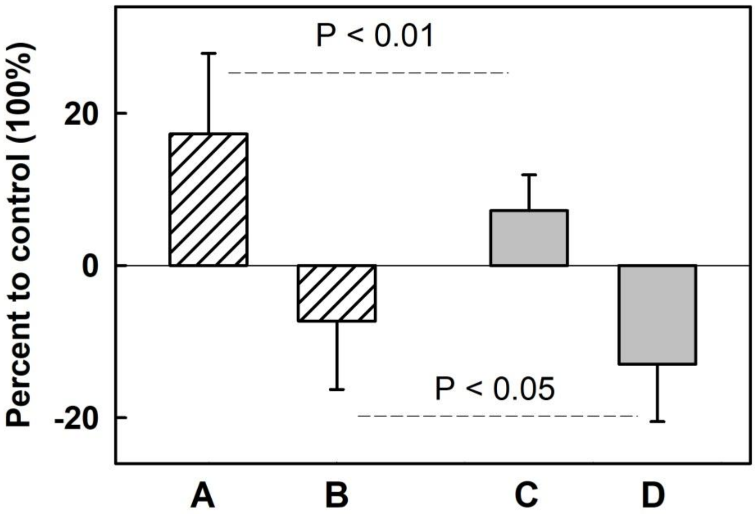

Fig. S26 Comparison of the systolic BP transient changes between the first and the second administration of GSH/SeO_3^{2-}. Effect of the first **(A,B)** and the second **(C,D)** administration of the GSH/SeO_3^{2-} (75/12.5 in µmol kg^{-1}) mixture on transient increase **(A,C)** and decrease **(B,D)** in systolic BP expressed as a percentage. Systolic BP before the first and the second administration was set to 100% and was considered as control. Changes are expressed as a difference to the control. Statistics: paired Student's t-test P=0.034, **B** vs. **D** and P = 0.003, **A** vs. **C**

Tab. S1 Description of 35 APW-Ps derived from APW

Ten points **a - j** (in bold letters) are from Fig. 13 and they mark the values of blood pressure (BP) and time that are used to define (calculate) specific APW-Ps.

(a) Systolic blood pressure in mmHg; point **c** or **f**
(b) Heart rate in min^{-1}; 60 / (**j** – **a**); (**j** – **a**) represents time interval between **a** and **j**, **a** and **j** are two reference points to diastolic BP value
(c) Systolic area in mmHg s; integral BP of **a** to **h**; **h** refers to BP at the dicrotic notch (dicrotic BP)
(d) dP/dt_{max} in mmHg ms^{-1}; maximum derivative at the point **b**; P is BP in mmHg
(e) dP/dt_{max} relative level; relative level (RL) of point **b**; (**b** – **a**) / (**c** (or **f**) – **a**) in mmHg/mmHg (dimensionless)
(f) dP/dt_d in mmHg ms^{-1}; negative derivative at the point **i**; the point **i** is the BP in the middle of the time interval between **h** and **j**
(g) dP/dt_d relative level, relative level of point **i**; (**i** – **a**) / (**c** (or **f**) – **a**) in mmHg/mmHg (dimensionless)
(h) $dP/dt_d - dP/dt_{max}$ in s; time interval between **b** and **i**, $dP/dt_d - dP/dt_{max}$ = (**i** – **b**)
(i) $dP/dt_d - dP/dt_{min}$ in s; time interval between **g** and **i**, $dP/dt_d - dP/dt_{min}$ = (**i** – **g**); dP/dt_{min} is maximum negative derivative at the point **g**
(j) Diastolic blood pressure in mmHg; the point **a** or **j**
(k) Pulse BP in mmHg; (**c** – **a**) or (**f** – **a**)
(l) Diastolic area in mmHg s; integral BP of **h** to **j**
(m) dP/dt_{min} in mmHg ms^{-1}; dP/dt_{min} is maximum negative derivative at the point **g**
(n) dP/dt_{min} relative level, relative level of point **g**; (**g** – **a**) / (**c** (or **f**) – **a**) in mmHg/mmHg (dimensionless)
(o) dP/dt_{min} delay in s; delay in s of point **g**; (**g** – **a**) time interval between **a** and **g**
(p) dP/dt_d delay in s; delay in s of point **i**; (**i** – **a**) time interval between **a** and **i**
(q) $dP/dt_d - dP/dt_{max}$ in mmHg; (**i** – **b**) BP difference between **b** and **i**
(r) $dP/dt_d - dP/dt_{min}$ in mmHg; (**i** – **g**) BP difference between **g** and **i**

(aa) Systolic blood pressure in mmHg; point **c** or **f**. Plot (aa) is the same as (a)
(bb) Anacrotic notch in mmHg; BP at the point **d**
(cc) Anacrotic notch relative level; relative level of point **d**; (**d** – **a**) / (**c** (or **f**) – **a**) in mmHg/mmHg (dimensionless)
(dd) Anacrotic notch delay in ms; delay in ms of point **d**; (**d** – **a**) time interval between **a** and **d**
(ee) Anacrotic notch relative delay; relative delay (RD) of point **d**; (**d** – **a**) / (**j** – **a**) in ms/ms (dimensionless)
(ff) [Dicrotic notch (DiN) in s] – [Anacrotic notch (AnN) in s] in s; (**h** – **d**) time interval between **d** and **h**
(gg) [(DiN – AnN) in s] / [dP/dt_{min} in mmHg μs^{-1}] in s/mmHg μs^{-1}; (**h** – **d**) / **g**
(hh) [(DiN – AnN) in s] / [dP/dt_{max} in mmHg μs^{-1}] in s/mmHg μs^{-1}; (**h** – **d**) / **b**

(ii) [AnN in ms] – [1Max (point **c** or the 1st. maximum) in ms] in ms; (**d** – **c**) time interval between **c** and **d**
(jj) Augmentation index relative; (**f** – **c**) / (**f** – **a**) in mmHg/mmHg (dimensionless)**
(kk) Dicrotic notch in mmHg; BP at the point **h**
(ll) Dicrotic notch relative level; relative level of point **h**; (**h** – **a**) / (**c** (or **f**) – **a**) in mmHg/mmHg (dimensionless)
(mm) Dicrotic notch delay in ms, delay in ms of point **h**; (**h** – **a**).time interval between **a** and **h**
(nn) Dicrotic notch relative delay; relative delay of point **h**; (**h** – **a**) / (**j** – **a**); in ms/ms (dimensionless)
(oo) [DiN in mmHg] – [AnN in mmHg] in mmHg; (**h** – **d**) BP difference between **d** and **h**
(pp) [(DiN – AnN) in mmHg] / [dP/dt_{min} in mmHg ms^{-1}] in mmHg/mmHg ms^{-1}; (**h** – **d**) / **g**
(qq) [(DiN – AnN) in mmHg] / [dP/dt_{max} in mmHg ms^{-1}] in mmHg/mmHg ms^{-1}; (**h** – **d**) / **b**
(rr) [AnN in mmHg] – [1Max (point **c** or the 1st. maximum) in mmHg] in mmHg; (**d** – **c**) BP difference between **c** and **d**
*Units in plots (gg), (hh), (pp) and (qq) are informative only
The plot of augmentation index relative (jj**) was not possible to determine in cases when the highest point at APW (Fig. 13) was "**c**" and not "**f**" and it was set to zero [1]

References:

1. Kurakova L, Misak A, Tomasova L, Cacanyiova S, Berenyiova A, Ondriasova E, Balis P, Grman M, Ondrias K (2020) Mathematical relationships of patterns of 35 rat haemodynamic parameters for conditions of hypertension resulting from decreased nitric oxide bioavailability. Exp Physiol 105:312-334. https://doi.org/10.1113/ep088148

2. Balis P, Berenyiova A, Misak A, Grman M, Rostakova Z, Waczulikova I, Cacanyiova S, Domínguez-Álvarez E, Ondrias K (2023) The Phthalic Selenoanhydride Decreases Rat Blood Pressure and Tension of Isolated Mesenteric, Femoral and Renal Arteries. Molecules 28. https://doi.org/10.3390/molecules28124826
